# Genome-Wide Mining of Wheat *DUF966* Gene Family Provides New Insights Into Salt Stress Responses

**DOI:** 10.3389/fpls.2020.569838

**Published:** 2020-08-28

**Authors:** Xiaoyi Zhou, Xiaoguo Zhu, Wenna Shao, Jinghan Song, Wenqiang Jiang, Yiqin He, Junliang Yin, Dongfang Ma, Yongli Qiao

**Affiliations:** ^1^Hubei Collaborative Innovation Center for Grain Industry/Engineering Research Center of Ecology and Agricultural Use of Wetland, Ministry of Education/College of Agriculture, Yangtze University, Jingzhou, China; ^2^Shanghai Key Laboratory of Plant Molecular Sciences, College of Life Sciences, Shanghai Normal University, Shanghai, China

**Keywords:** *TaDUF966*, phylogenetic analysis, *Triticum aestivum* L., transcriptome analysis, abiotic stresses, virus induced gene silencing

## Abstract

Domain of unknown function (DUF) proteins constitute a great deal of families of functionally uncharacterized proteins in eukaryotes. The DUF966 gene family is found in monocotyledons, dicotyledons, mosses, and other species. However, little is known about the functions of DUF966 genes in wheat (*Triticum aestivum* L.). In this study, we identified and characterized the TaDUF966 gene family members in wheat by *in silico* analysis. A total of 28 TaDUF966 proteins were identified in wheat. Phylogenetic analysis divided these proteins into two groups (Groups I and II). Proteins in each group showed a highly conserved DUF966 domain and conserved motif distribution, implying their functional conservation. Analysis of gene expression profiling data showed that some TaDUF966 genes were induced by salt stress. We further confirmed the role of TaDUF966-9B in salt stress using virus induced gene silencing (VIGS) assay. Compared with the empty vector control, the TaDUF966-9B knockdown plants exhibited severe leaf curling at 10 days post-inoculation with BSMV under salt stress, suggesting that TaDUF966 genes play a vital role in salt stress tolerance in wheat. Taken together, these results expand our knowledge of the evolution of the DUF966 gene family in wheat and promote the potential application of these genes in wheat genetic improvement.

## Introduction

Wheat (*Triticum aestivum* L.) is one of the most important grain crops in the world and the main source of food for the majority of the human population (Yin J. et al., 2018). Abiotic stresses, such as drought, salinity, and extreme temperatures, negatively impact the growth, development, quality, and yield of wheat and other crops (Zhu et al., 2019a). Domain of unknown function (DUF) is a general term used for many domains in the Pfam database that have not been confirmed. These domains have two distinct characteristics: a relatively conserved amino acid sequence and the function of this domain is unknown (Bateman et al., 2010). DUF proteins are reportedly involved in abiotic stress tolerance in plants. For example, the wheat *TaWTF1* gene, which encodes the Domain of Unknown Function 860 (DUF860) protein, contains stress- and hormone-responsive elements in its promoter region and is induced by heat stress at the seedling and flowering stages (Qin et al., 2013). In rice (*Oryza sativa* L.), the expression of *OsDUF936.3*, *OsDUF936.5*, and *OsDUF936.6* is significantly increased under salt stress conditions, and overexpression of *OsDUF936.6* in *Escherichia coli* significantly improves tolerance to salt stress (Li et al., 2017). Additionally, *OsSIDP366*, a *DUF1644* gene, is a positive regulator of drought and salt stress tolerance in rice (Guo et al., 2016). In *Ammopiptanthus mongolicus*, the *AmDUF1517* gene is potentially involved in the regulation of cold tolerance (Gu and Cheng, 2014).

Increasing studies show that genes containing DUF domains are involved in plant growth and developmental processes. Rice REL2 (ROLLED and ERECT LEAF 2 (OsREL2)) is the first DUF domain-containing protein shown to be involved in the regulation of leaf morphology (Yang et al., 2016). Additionally, rice *BEAK-SHAPED GRAIN 1* (*BSG1*), a *DUF640* gene, determines grain shape and size, probably by controlling cell division and expansion in the grain hull (Yan et al., 2013). In *Arabidopsis thaliana*, silencing of the *At2g23470* gene, which encodes a member of the DUF647 protein family, results in severe infertility (Li and Zhao, 2012). Li et al. (2016) determined the role of *DUF1313* gene family members in photoperiod sensitivity in maize (*Zea mays* L.). In the woody perennial plant *Populus deltoides*, PdDUF231A, a DUF231 family protein, is involved in xylan acetylation and cellulose biosynthesis (Yang et al., 2017). In addition to their role in plant growth, development, and abiotic stress response, DUF proteins are also involved in the biotic stress response. For example, silencing of the *OsDUF500* gene in rice increases resistance to bacterial blight strain PXO99 (Li et al., 2012). The plant DUF538 protein is predicted as a partial structural homolog of the bactericidal/permeability-increasing (BPI) protein of the mammalian innate immune system, which provides the first line of defense against different pathogens including bacteria, fungi, viruses, and parasites. Experimental evidence shows that exogenous application of the purified fused product of *Celosia* DUF538 affects bacterial growth, possibly by binding to the bacterial membrane, similar to the BPI protein (Gholizadeh and Kohnehrouz, 2013).

The DUF966 protein superfamily is found in monocotyledons, dicotyledons, mosses, and other species (Luo and Tian, 2017). Proteins in this superfamily contain one or two highly conserved DUF966 domains. In tomato (*Solanum lycopersicum*), *JWS19* (a *DUF966* gene) was transiently and rapidly induced by salt stress and was isolated from salt-treated roots (Tirajo, 2005). Although the molecular function of *DUF966* genes is not fully understood, the available evidence strongly suggests that *DUF966* family genes are involved in abiotic stress tolerance. For example, in rice, overexpression of the new stress suppressor gene, *DUF966-stress repressive gene 2* (*OsDSR2*), dramatically increased the sensitivity to salt and drought stresses and reduced sensitivity to abscisic acid (ABA) (Luo et al., 2014). In Arabidopsis, the *AtST39* gene (At3g46110) plays a positive regulatory role in salt and drought stress resistance pathways (Wang, 2014).

Although the *DUF966* genes play vital roles in plants, to the best of our knowledge, their functions remain unexplored in wheat. In this study, we conducted a comprehensive analysis of *TaDUF966* family genes in abiotic stress tolerance in wheat, based on their genomic sequence and transcriptome data. A total of 28 *TaDUF966* family genes were identified, and their conserved motifs, genomic structure, evolutionary relationship, and functional classification were investigated. Moreover, expression analysis of *TaDUF966* genes was conducted, and *cis*-acting elements in their promoter regions were examined. Our results provide valuable information for understanding the classification and putative functions of *DUF966* genes.

## Materials and Methods

### Plant Material and Stress Treatment

Common wheat (*Triticum aestivum* L.) cultivar Zhengmai was used in this study. Plants were grown at 26°C under a 16 h day/8 h dark photoperiod. To conduct salt stress treatments, plants were treated with 150 mM sodium chloride (NaCl) at the stages of seedling with one and half leaves. Leaf and root samples were collected at 2, 4, 8, 12, 24, and 96 h after the salt treatment (Jiang et al., 2019). The experiment was performed with three independent biological replicates.

### Identification of *TaDUF966* Genes and Sequence Analysis

The *TaDUF966* gene family members were identified by *in silico* analysis of the wheat reference genome assembly (RefSeq v.1.1; International Wheat Genome Sequencing Consortium [IWGSC]) (https://wheat-urgi.versailles.inra.fr/Seq-Repository/Assemblies) (He et al., 2020). To obtain the amino acid sequences of the putative TaDUF966 family proteins, BLASTp (Basic Local Alignment Search Tool protein) analysis was conducted using seven rice DUF966s (OsDSRs) (http://rice.plantbiology.msu.edu/index.shtml) (Luo and Tian, 2017) and five Arabidopsis (https://www.arabidopsis.org/index.jsp) DUF966s (AtUOFs) as query sequences against the wheat reference IWGSC v.1.1 (Jiang et al., 2020a). To ensure the reliability of TaDUF966 protein sequences, a cut-off value (E < 1 × 10^−10^) was used. The TaDUF966 family members were further screened and validated using the SMART (Letunic et al., 2004) and Pfam databases (version 32.0, PF06136) (http://pfam.xfam.org/).

### Phylogenetic Analysis, Chromosomal Locations, and Duplication Patterns

Phylogenetic analysis of TaDUF966 proteins was conducted using the LG model (Le and Gascuel, 2008) based maximum likelihood (ML) method in MEGA 7.0 (Kumar et al., 2016). The test guidance was repeated 1,000 times (Felsenstein, 1985) to calculate the support for each node. A midpoint-rooted base tree was drawn using the Interactive Tree of Life (IToL, version 3.2.317, http://itol.embl.de) (Zhu et al., 2019b). The GFF3 gene annotation file was obtained from the wheat database IWGSC v.1.1, and *TaDUF966* gene annotations were extracted. Chromosomal start and stop sites and the chromosomal location of each *TaDUF966* gene were used to draw the physical map with the MapInspect software. All possible homolog of the *DUF966* genes in each subgenome of common wheat were determined using “all-against-all” BLAST, with an E-value cut-off of 1 × 10^−10^ and identity > 75% (Letunic et al., 2004). The R package “circlize” was used to draw a graph showing its position and homology relationship (Fang et al., 2020). The non-synonymous (Ka) and synonymous (Ks) substitution rates were calculated using DnaSP 6.0 (Rozas et al., 2017) to diagnose the form of sequence evolution.

### Gene Structure and Predicted Protein Feature Analyses

The predicted TaDUF966 protein sequences were analyzed using protein identification and analysis tools available online. The protein length, isoelectric point (pI), molecular weight (MW), instability, and index were predicted using the ProtParam (Gasteiger et al., 2005) tool (https://web.expasy.org/protparam/). The subcellular localization of these proteins was predicted using the Plant-mPLoc online tool (http://www.csbio.sjtu.edu.cn/bioinf/plant-multi/) (Chou and Shen, 2010).

The MEME (v5.0.3) (Bailey and Elkan, 1994) suite analysis tool and MAST (http://meme-suite.org/tools) motif search tool were used to identify conserved motifs in TaDUF966 proteins. The known DUF966 protein sequences, including AtDUF966 and OsDSR, were used for training the parameter settings. Then, the trained parameters were used to identify conserved TaDUF966 motifs as follows: each sequence may contain any number of non-overlapping repeats of each motif; number of different motifs (1–10); motif width = 6–50 amino acids (aa). The functions of predicted motifs were analyzed using InterPro (http://www.ebi.ac.uk/interpro) and SMART motif search programs (http://coot.embl-heidelberg.de/SMART). In addition, multiple sequence alignment analysis of TaDUF966 proteins was conducted using ClustalX2 (Kim and Joo, 2010).

### Prediction of *Cis*-Acting Elements

To analyze putative *cis*-elements in *TaDUF966* gene promoters, the 1,500 bp sequence upstream of the start codon of each gene was searched in the Plant-CARE database (Bailey and Elkan, 1994). The identified motifs were analyzed using the Fisher test, and those with an adjusted *p*-value < 0.05 were considered significantly enriched.

### Analysis of Wheat Transcriptome Under Salt Stress

Transcriptome responses of two wheat cultivars to salt stress RNA-seq datasets generated previously under an abiotic stress treatments were downloaded from NCBI and mapped to the wheat reference genome using HISAT2. Then, genes were assembled using cufflinks to inspect the expression levels of *TaDUF966* genes (normalized by TPM, transcripts per kilobase of exon model per million mapped reads). The R package “pheatmap” was used to draw the pheatmap of *TaDUF966* genes (Zhu et al., 2019c).

### RNA Extraction and Quantitative Real-Time PCR (qRT-PCR) Analysis

Total RNA was isolated from root samples from plants treated with or without NaCl using the TRIzol Reagent (Invitrogen, Carlsbad, CA, U.S.A). The isolated total RNA samples were quantified using the NanoDrop spectrophotometer (Thermo Fisher Scientific) and then treated with DNase I (Thermo Fisher Scientific) to remove any residual DNA contamination. The DNA-free RNA samples were converted to cDNA using a cDNA synthesis kit (Thermo Fisher Scientific). Gene-specific primers were designed using Primer Premier 5.0 (**Table S1**). Then, quantitative PCR (qPCR) was carried out using Maxima SYBR Green/ROX qPCR Master Mix (Zhang et al., 2019; Chen et al., 2020). The *EF-1α* gene was used as an internal reference. Three independent biological replicates, each containing three technical replicates, were conducted for each sample in both control and NaCl treatments. Gene expression was quantified using the 2^−△△Ct^ method (Yin J. L. et al., 2018).

### Virus-Induced Gene Silencing (VIGS) Experiment in Wheat

Two cDNA fragments from different sites silenced TaDUF966-9B. Using BLAST analysis in NCBI, these fragments are specific (Zhu et al., 2018a). Primers for constructs in plant transformation were designed using Primer Premier 5.0 and are listed in **Table S2**. *Barley stripe mosaic virus* (BSMV) consists of *α*, *β*, and *γ* three RNA chains. According to the manufacturer’s instructions of RiboMAXTM, using the RiboMAXTM large-scale RNA production system-T7 (Petty et al., 1990) and Ribom7G Cap analogues (Promega, Madison, Wisconsin, USA) *in vitro* linearized plasmids containing the tripartite BSMV genome were sealed at the transcripts. The second leaf of the two-leaf wheat seedling was mechanically wiped with BSMV transcript and incubated at 25°C. Use BSMV: TaPDS (TaPDS, wheat octahydrolysin desaturase) as a positive control.

Control plants were treated with 1× Fes buffer (0.1M glycine, 0.06M K2HPO4, 1% w/v tetrasodium pyrophosphate, 1% w/v bentonite, and 1% w/v celite, pH 8.5) devoid of BSMV transcripts (Zhu et al., 2018b). At 10 dpi, 150 mM NaCl was poured and samples were taken at 1 d and 6 d for RNA isolation, followed by qRT-PCR analysis and phenotypic analysis. qRT-PCR primers were designed using Primer Premier 5.0 and are listed in **Table S3**. The experiment was repeated at least three times.

## Results

### Identification and Classification of *TaDUF966* Genes

**Figure 1:**
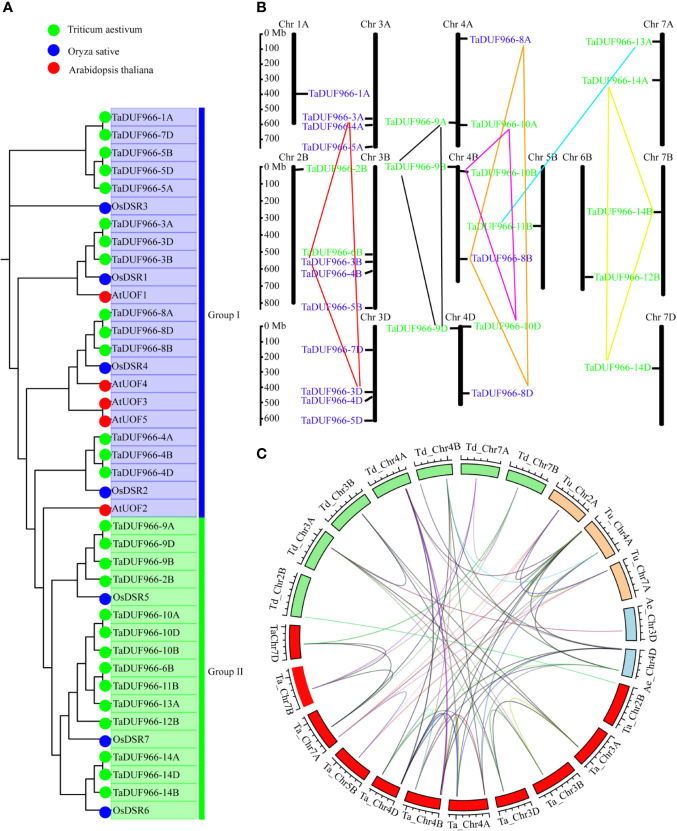
Phylogenetic analysis of *DUF966* gene family. **(A)** Phylogenetic tree of DUF966 proteins from wheat, Arabidopsis, and rice. Predicted amino acid sequences were aligned using ClustalW2 sequence alignment program. Different groups were marked by different colors. Blue, Group I; green, Group II. Green round, 28 DUF966 proteins from wheat; blue round, 7 DUF966 proteins from rice; red round, 5 DUF966 proteins from Arabidopsis. **(B)** Chromosomal localization of the 28 DUF966 genes in the wheat genome. Positions were determined in the map. Different groups of TaDUF966 are represented in different colors. Blue, Group I; green, Group II. **(C)** Genome-wide syntenic analysis among common wheat (Ta, AABBDD, red box) and progenitor species *Aegilops tauschii* (Ae, DD, blue box), *T. urartu* (Tu, AA, orange box), and *T. dicoccoides* (Td, AABB, green box). The lines between the two chromosomes link the pair of orthologous DUF966 genes.

**Figure 2:**
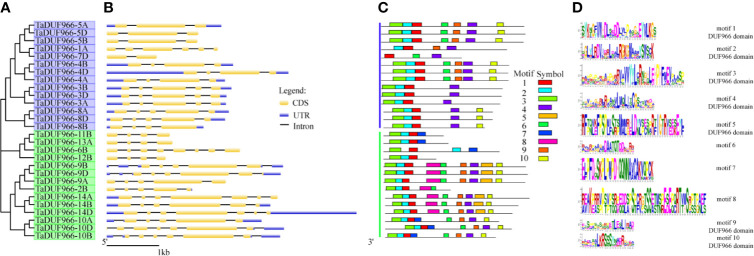
Phylogenetic relationships, exon–intron structures and motif compositions of wheat DUF966 (TaDUF966) proteins. **(A)** The tree was created with bootstrap of 1,000 by Maximum Likelihood (ML) method in MEGA7. **(B)** Exon–intron structure analyses were conducted using the GSDS database. Bar represents 1 kb of exon or intron. The intron length of each TaDUF966 gene is displayed the same length. **(C)** Motif composition of DUF966 proteins illustrated by MAST. **(D)** The logo map of conserved sequences of 10 putative motifs of the DUF966 protein.

A total of 28 TaDUF966 proteins were identified from wheat reference genome using seven rice and five Arabidopsis protein sequences as query (**Table S4**). These wheat proteins were named according to the following rules: (1) “TaDUF966” stands for the hexaploid wheat DUF966 gene family; (2) Arabic numerals represent the gene number; (3) “-A/B/D” represents the wheat subgenome to which the gene belongs [**Table 1**; (Song et al., 2019)]. We identified 7 triads TaDUFs (**Table S5**). To better analyze the phylogenetic relationships among wheat and other plant DUF966 proteins, we constructed a phylogenetic tree using DUF966 sequences from wheat, rice, and Arabidopsis (**Figure 1A**). All DUF966 proteins were divided into two major groups (Groups I and II) (**Figure 2A**). Each group contained 14 members, and TaDUF966 sequences were present in both groups. Group II contained DUF966 proteins from only wheat and rice, indicating that *DUF966* genes in wheat were more closely related to those in rice (**Figure 1A**).

**Table 1:** Information of the *DUF966* gene family in common wheat as well as its wild relatives.

Designation	Protein ID	Chr	Location
TaDUF966-1A	TraesCS1A02G359300LC	1A	396288719–396292574
TaDUF966-2B	TraesCS2B02G035500	2B	17040005–17041737
TaDUF966-3A	TraesCS3A02G318800	3A	561034605–561037042
TaDUF966-3B	TraesCS3B02G347200	3B	556645507–556648123
TaDUF966-3D	TraesCS3D02G312500	3D	426932130–426934648
TaDUF966-4A	TraesCS3A02G356700	3A	604343231–604346064
TaDUF966-4B	TraesCS3B02G389500	3B	612806847–612809862
TaDUF966-4D	TraesCS3D02G350800	3D	461954930–461959124
TaDUF966-5A	TraesCS3A02G535000	3A	746557659–746560196
TaDUF966-5B	TraesCS3B02G612100	3B	829508771–829510876
TaDUF966-5D	TraesCS3D02G540500	3D	611889001–611891152
TaDUF966-6B	TraesCS3B02G490800LC	3B	513678404–513683585
TaDUF966-7D	TraesCS3D02G207500LC	3D	153226727–153227568
TaDUF966-8A	TraesCS4A02G038000	4A	30147071–30150091
TaDUF966-8B	TraesCS4B02G267700	4B	541363253–541365268
TaDUF966-8D	TraesCS4D02G267300	4D	437950550–437953699
TaDUF966-9A	TraesCS4A02G280800	4A	589108224–589111612
TaDUF966-9B	TraesCS4B02G032000	4B	23818716–23822988
TaDUF966-9D	TraesCS4D02G029700	4D	13196531–13201007
TaDUF966-10A	TraesCS4A02G311800	4A	603496846–603499370
TaDUF966-10B	TraesCS4B02G000600	4B	556850–559568
TaDUF966-10D	TraesCS4D02G001100LC	4D	1257755–1260520
TaDUF966-11B	TraesCS5B02G319600LC	5B	347977620–347978519
TaDUF966-12B	TraesCS6B02G681100LC	6B	648273617–648274459
TaDUF966-13A	TraesCS7A02G117000LC	7A	49204692–49205642
TaDUF966-14A	TraesCS7A02G281800	7A	306344545–306348514
TaDUF966-14B	TraesCS7B02G179600	7B	265430053–265433243
TaDUF966-14D	TraesCS7D02G280300	7D	272597370–272602133
TuDUF966-1A	TuG1812G0200002574.01.T01	2A	305051841–305053878
TuDUF966-2A	TuG1812U0000104100.01.T01	2A	305051841–305054142
TuDUF966-3A	TuG1812G0300003957.01.T01	3A	596116508–596119041
TuDUF966-4A	TuG1812G0400000077.01.T01	4A	4169025–4170232
TuDUF966-5A	TuG1812G0400000309.01.T01	4A	15533663–15538158
TuDUF966-6A	TuG1812G0400003116.01.T01	4A	557235547–557238342
TuDUF966-7A	TuG1812U0000204800.01.T01	4A	4271740–4272947
TuDUF966-8A	TuG1812G0700000981.01.T01	7A	51135931–51136917
TuDUF966-9A	TuG1812U0000267400.01.T01	7A	51135931–51136917
TuDUF966-10Un	TuG1812S0000575500.01.T01	Un	40–3452
TdDUF966-1B	TRIDC2BG002780.1	2B	12906663–12908163
TdDUF966-2A	TRIDC3AG045980.1	3A	559407269–559409302
TdDUF966-3A	TRIDC3AG051330.1	3A	600886081–600887613
TdDUF966-4A	TRIDC3AG075710.1	3A	750804499–750806588
TdDUF966-5B	TRIDC3BG052150.1	3B	569063112–569065233
TdDUF966-6B	TRIDC3BG057930.1	3B	623877065–623879673
TdDUF966-7B	TRIDC3BG087140.1	3B	836134473–836136404
TdDUF966-8A	TRIDC4AG005290.1	4A	30277701–30278927
TdDUF966-9A	TRIDC4AG043530.1	4A	582115269–582119628
TdDUF966-10A	TRIDC4AG047220.1	4A	595442094–595444369
TdDUF966-11B	TRIDC4BG001500.1	4B	4480304–4482366
TdDUF966-12B	TRIDC4BG005350.1	4B	22750249–22754174
TdDUF966-13B	TRIDC4BG046350.1	4B	547074740–547076829
TdDUF966-14A	TRIDC7AG037390.1	7A	304364007–304367206
TdDUF966-15B	TRIDC7BG028580.1	7B	275715194–275718556
AeDUF966-1D	AET2Gv20621000.2	2D	340642693–340647592
AeDUF966-2D	AET3Gv20385200.1	3D	156184974–156185962
AeDUF966-3D	AET3Gv20731600.1	3D	434177980–434179497
AeDUF966-4D	AET3Gv20802400.1	3D	469721272–469724009
AeDUF966-5D	AET3Gv21248000.1	3D	623842697–623845442
AeDUF966-6D	AET4Gv20004000.1	4D	894539–897603
AeDUF966-7D	AET4Gv20059700.1	4D	14460999–14465228
AeDUF966-8D	AET4Gv20657800.1	4D	443956872–443958973
AeDUF966-9D	AET7Gv20681900.2	7D	274570324–274576316

### Chromosomal Location of *TaDUF966*

The identified 28 *TaDUF966* genes were distributed on 13 chromosomes, and each chromosome contained 1–4 *TaDUF966* genes (**Figure 1B** and **Table 1**). The 24 genes of the TaDUF966 gene were located on chromosome 3 (3A, 3B, and 3D, 11 genes), chromosome 4 (4A, 4B, and 4D, 9 genes) and chromosome 7 (7A, 7B, and 7D, 4 genes). The other 4 chromosomes (Chr1A, Chr2B, Chr5B, and Chr6B) contained only one TaDUF966 gene for each chromosome. Most members of Group I were distributed on 3 chromosomes (3A, 3B, and 3D), while members of Group II were evenly distributed on 13 chromosomes.

### Homologous Gene Pairs and Synteny Analysis

The homologous pairs of DUF966 were identified in the common wheat ancestral species, *Triticum urartu* (AA), *Triticum dicoccoides* (AABB), and *Aegilops tauschii* (DD); 10, 15, and 9 homologous pairs of DUF966 were identified, respectively, named as TuDUF966, TdDUF966, and AeDUF966 based on the naming rules. The location and other details of these genes are summarized in **Table 1**. Comparisons between *TuDUF966 vs. TdDUF966*, *TaDUF966 vs. TdDUF966*, *TaDUF966 vs. TuDUF966*, *TaDUF966 vs. AeDUF966*, and *TaDUF966 vs. TaDUF966* revealed 2, 21, 12, 6, and 21 pairs of putative paralogs, respectively (**Figure 1C**). To better understand the evolutionary factors affecting the *DUF966* gene family, we calculated the Ka/Ks ratios among homologous pairs of *TaDUF966*, *TuDUF966*, *TdDUF966*, and *AeDUGF966* genes (**Table S6**). Most of the *DUF966* gene pairs showed Ka/Ks ratio < 1, indicating that these genes contained more synonymous than non-synonymous changes. Moreover, these genes were under negative selection pressure to protect the state of the ancestor. Three homologous pairs, including *TaDUF966-11B*/*TuDUF966-9A*, *TaDUF966-11B*/*TuDUF966-8A*, and *TaDUF966-11B*/*TaDUF966-13B*, showed Ka/Ks ratio > 1, indicating positive selection, accelerated evolution, and neofunctionalization. Analysis of duplication events of *TaDUF966* genes revealed that gene duplication occurred mainly among Group II genes (**Figure 1B**, **Table S6**). Of the 28 *TaDUF966* genes, 17 (60.7%) genes showed segmental duplication events. Furthermore, *TuDUF966* genes showed segmental and tandem duplications, while *TdDUF966* genes showed segmental duplications (**Table S6**). These data suggest that segmental duplication events played a key role in the expansion of the *DUF966* gene family in wheat.

### Analysis of Gene Structure and Motif Composition in *TaDUF966* Genes

To investigate the structure of TaDUF966 genes, we extracted their detailed information from the GFF3 file of the reference genome (Jiang et al., 2020b). The results showed that all TaDUF966 genes contain introns. Comparative exon–intron structure analysis revealed the presence of 2–6 exons in Group I members and 4–8 exons in Group II members (**Figure 2B**). Motif usually refers to the basic structure that constitutes any kind of characteristic sequence. It is a subset of the structural domain, and its function is to reflect the various biological functions of the structural domain. The prediction of protein motifs is a useful protein analysis method. Analysis of 28 TaDUF966 proteins using MEME revealed 10 motifs (**Figures 2C, D****)**. Details of these 10 motifs are shown in **Table S7**. Each TaDUF966 protein contains 3–10 motifs, and most TaDUF966 proteins contain motifs 1, 2, and 3. In the phylogenetic tree, we found that members with relatively similar genetic relationships have more similar motifs, which indicates that DUF966 members gathered in the same subgroup may have more similar biological effects. In addition, all TaDUF966 proteins contain the DUF966 conserved Motif 1. Motif analysis using NCBI BLAST showed that seven of these 10 motifs (motifs 1–5, 9, and 10) belong to the DUF966 conserved domain, while the remaining three motifs were uncharacterized. Multiple sequence alignment analysis of TaDUF966 proteins revealed that the amino acid sequence of the DUF966 domain is highly conserved (**Figure S1**), suggesting that this domain plays a key role in protein function.

### Analysis of TaDUF966 Protein Features

Protein feature analysis showed that TaDUF966 proteins contain an average of 422 aa (191 aa–644 aa) (**Table 2**). Other features of TaDUF966 proteins were as follows: average pI = 8.89 (range = 6.46–9.88); average MW = 46.1 kDa (range = 21.3–69.84 kDa); average instability index = 63.48 (range = 36.76–79.99) (**Table S8**). The instability index values indicate that TaDUF966 proteins are relatively unstable. The results of the subcellular localization prediction of all 28 TaDUF966 proteins showed that 27 proteins localized to the nucleus or chloroplast, whereas one protein localized to the cytoplasm.A

**Table 2:** Predicted amino acid sequence features of TaDUF966 proteins in wheat.

Designation	Domain Number	Alternativesplices	Length (aa)	Cds (bp)	Exon Number
TaDUF966-1A	2	1	401	1,206	6
TaDUF966-2B	1	2	292	879	5
TaDUF966-3A	1	2	379	1,140	5
TaDUF966-3B	1	1	387	1,164	5
TaDUF966-3D	1	2	384	1,155	5
TaDUF966-4A	1	1	406	1,211	4
TaDUF966-4B	1	1	408	1,227	4
TaDUF966-4D	1	1	405	1,218	4
TaDUF966-5A	1	1	456	1,371	3
TaDUF966-5B	1	1	454	1,365	3
TaDUF966-5D	1	1	460	1,383	3
TaDUF966-6B	1	1	420	1,263	8
TaDUF966-7D	1	1	257	774	2
TaDUF966-8A	1	1	396	1,191	5
TaDUF966-8B	1	1	367	1,104	4
TaDUF966-8D	1	1	395	1,188	5
TaDUF966-9A	1	2	513	1,542	5
TaDUF966-9B	1	1	515	1,548	5
TaDUF966-9D	1	1	522	1,569	6
TaDUF966-10A	1	1	508	1,527	6
TaDUF966-10B	1	2	494	1,485	8
TaDUF966-10D	1	1	565	1698	8
TaDUF966-11B	1	1	218	657	4
TaDUF966-12B	1	1	191	576	4
TaDUF966-13A	1	1	235	708	4
TaDUF966-14A	1	4	644	1,935	7
TaDUF966-14B	1	3	595	1,768	6
TaDUF966-14D	1	3	568	1,707	7

### Prediction of *Cis*-Acting Regulatory Elements in *TaDUF966* Gene Promoters

**Figure 3:**
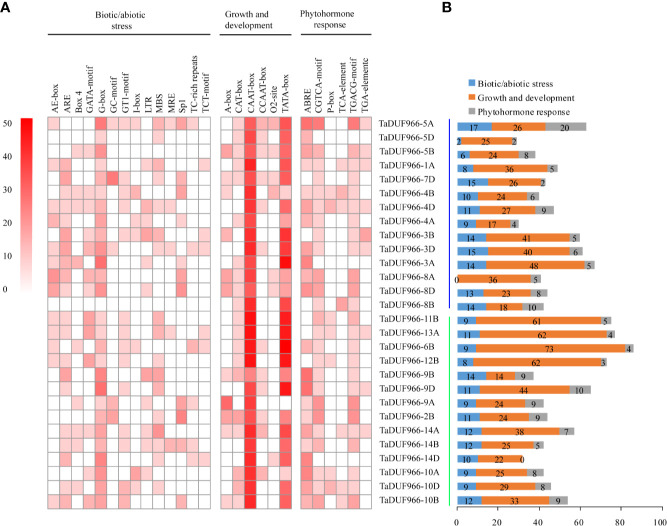
*Cis*-acting elements in the promoters of hexaploidy wheat *DUF966* genes. **(A)** *Cis*-acting elements in the promoters of DUF966 genes in wheat. The different shades of red color represent the number of *cis*-acting elements. **(B)** Biotic/abiotic stress, Growth and development, and Phytohormone responses refer to the types and number of *cis*-acting elements.

*Cis*-acting elements play important roles in the initiation of gene transcription. The *TaDUF966* gene promoters were predicted to possess *cis*-acting regulatory elements, which are involved in three different physiological and biochemical processes. (**Figure 3**, **Tables S9**, **S10**). Among the biotic/abiotic stress-responsive *cis*-elements, ARE (antioxidant response element) and G-box were highly enriched in *TaDUF966* gene promoters. Secondly, the CAAT-box and TATA-box (*cis*-elements related to growth and development) were also enriched in *TaDUF966* gene promoters. Among the phytohormone-related *cis*-acting elements, the ABA-responsive element (ABRE) was the most frequently identified in *TaDUF966* gene promoters. In addition, *Cis*-acting regulatory elements involved in the response to methyl jasmonate (MeJA), CGTCA-box and TGACG-box, were also identified in most of the *TaDUF966* gene promoters. In general, TaDUF966 gene promoter may respond to various endogenous and exogenous stimuli.

### Transcriptome Expression Profiling of *TaDUF966* Genes Under Salt Stress

**Figure 4:**
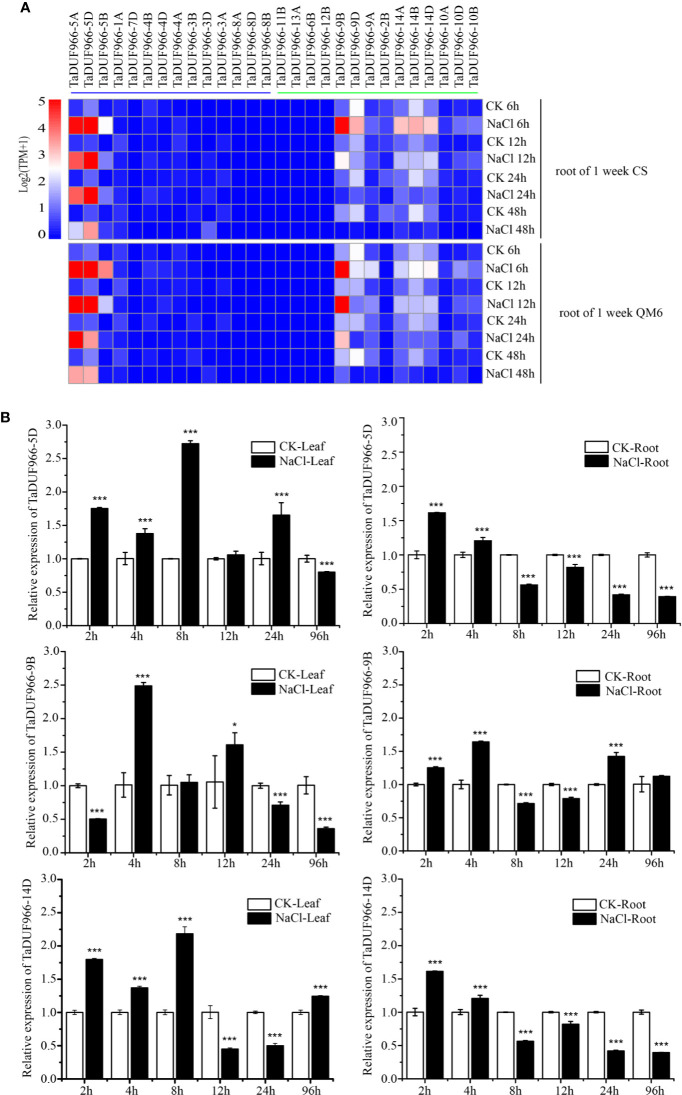
Expression analysis of wheat *TaDUF966* genes. **(A)** Expression profiles of TaDUF966 genes in root tissues, development stages, and NaCl treatment of wheat. The heat-map was established based on the R package “Pheatmap” and R function “boxplot”. The scale represents signal intensity of TPM values. **(B)** The qRT-PCR results. 10-day-old seedling leaves and roots were sampled after 0, 3, 6, 9, 12, and 24 h under stress conditions comprising 150 mM NaCl. t-tests were used to determine significant differences in expression patterns for CK and NaCl treatments (***p < 0.001, *P < 0.05).

Gene expression acts as a link between the transmission and realization of genetic information in organisms, and genome-wide analysis of gene expression patterns helps to identify the gene expression pathway and its regulation. In this study, RNA-seq data downloaded from NCBI were mapped to the wheat reference genome using HISAT2, and expression patterns of *TaDUF966* genes under salt stress were analyzed. The pheatmap, based on standardized TPM values, showed that salt stress strongly induced *TaDUF966-5*, -*9*, and -*14* (A, B, and D) but did not affect the expression of other genes (**Figure 4A**, **Table S11**) (Appels et al., 2018). The majority of *TaDUF966* genes showed low expression, whereas 9 genes showed high expression in two different wheat varieties. The expression of *TaDUF966* genes increased with the duration of salt treatment, reaching a peak at 6 h, followed by a gradual decline.

### Expression Analysis of *TaDUF966* Genes

To further reveal the potential functions of *TaDUF966* genes in association with abiotic stress (salt stress), we analyzed the expression patterns of several *TaDUF966* genes by qRT-PCR. According to transcriptome analysis, expression of *TaDUF966-5*, *-9*, and *-14* (A, B, and D) was relatively higher in salt-treated roots compared with control roots. The results of qRT-PCR analysis of *TaDUF966-5D*, *TaDUF966-9B*, and *TaDUF966-14D* were consistent with RNA-seq data. These three genes showed higher expression levels in the root and leaf tissues of wheat plants treated with NaCl compared with the control. Expression levels of these three genes increased in leaf tissues at 4 or 8 h after NaCl treatment but decreased in root tissues at 8 h (**Figure 4B**). In particular, the expression level of TaDUF966-9B increased in root tissues at 2 h after NaCl treatment, decreased in root tissues at 8 h, and was induced by salt stress 24 h later. Therefore, TaDUF966-9B may be better induced by salt stress. In subsequent trials we focused on this gene.

### Silencing of *TaDUF966-9B* Reduces Salt Tolerance in Wheat

**Figure 5:**
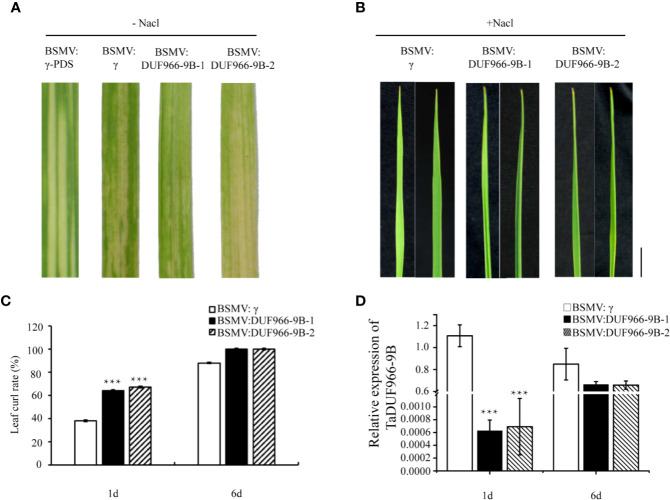
Silencing of *TaDUF966-9B* decreases salt tolerance in wheat. **(A)** BSMV: TaPDS showed photobleaching at 10 dpi; Mock: wheat leaves treated with 1× Fes buffer. **(B)** At 10 dpi, 150 mM NaCl was poured and sampled, these leaves were sampled at 10 dpi for phenotypic analysis. Bars, 1 cm. **(C)** Statistics of leaf curl rate in TaDUF966-9B-knockdown plants treated with 150 mM NaCl at 1 and 6 dpi. Values represent mean + standard errors of three independent assays. Differences were assessed using Student’s t-tests. **(D)** Silencing efficiency assessment of TaDUF966 in the 1 and 6 dpi of TaDUF966-9B-knockdown plants treated with 150 mM NaCl (***p < 0.001).

To further confirm the function of *TaDUF966-9B*, we performed a virus-induced gene silencing (VIGS) assay. Silencing of the *TaDUF966-9B* gene was achieved using two constructs (*BSMV: TaDUF966-9B-1* and *BSMV : TaDUF966-9B-2*), each carrying an approximately 250 bp fragment (**Figure S2**) of the corresponding *TaDUF966-9B* gene, respectively, applied as independent treatments. All plants inoculated with BSMV showed mild chlorotic mosaic symptoms at 10 days post-inoculation (dpi), while leaves inoculated with *BSMV : TaPDS* showed photobleaching (**Figure 5A**), indicating that the BSMV-induced gene silencing system was functional. The expression of each homolog was also analyzed by qRT-PCR. At 10 days after friction-implanted virus, the leaf curling phenotype was significantly stronger in plants expressing either construct than in plants carrying the empty vector control (**Figures 5B, C****)**, and the level of *BSMV : TaDUF966-9B-1/2* mRNA in NaCl-treated plants was significantly lower than that in the empty vector control (**Figure 5D**). These results indicate that the *TaDUF966-9B* gene is involved in salt stress tolerance in wheat.

## Discussion

Advances in high-throughput sequencing over the past two decades have increased the gap between genome sequence data and gene function information (Yin et al., 2020). Additionally, the number of DUF gene families has increased in the Pfam database (Bateman et al., 2010). Although the origin, diversity, and preliminary biological functions of these DUF gene families have been investigated in many studies, elucidating the biological functions of these genes, particularly in wheat, remains challenging.

The current Pfam database 32.0 contains 17,939 gene families, of which 3,961 gene families (22%) represent DUFs (Chalupska et al., 2008). Bioinformatics analysis shows that the *DUF966* gene family is widely distributed in rice, tomato, and Arabidopsis. In this study, a total of 28 *TaDUF966* genes were identified in common wheat. These genes were distributed on 13 chromosomes, particularly on chromosomes 3, 4, and 7; there are six *TaDUF966* genes, which are identified on A, B, and D subgenomes, respectively (**Figure 1B**). The other 10 *TaDUF966* gene family members did not appear simultaneously on three homeologous chromosomes, indicating that *TaDUF966* genes might have been amplified by replication during evolution, although a small number of genes could have been selectively deleted during evolution. This is consistent with previous research (Luo and Tian, 2017). Moreover, TaDUF966 proteins contained different numbers of motifs (3 to 10), but only one or two highly conserved DUF966 domains (**Table 2**), but functions of each domain remain to be investigated.

In addition to wheat, *DUF966* genes have also been identified in other plant species such as Arabidopsis, rice, maize, soybean, sorghum, tomato, grape, poplar, pawpaw, and alfalfa (Luo et al., 2014). A previous study showed that the rice DUF966 gene, *OsDSR2*, and tomato DUF966 gene, *JWS19*, play a vital role in response to salt stress and simulated drought stress (Tirajo, 2005; Luo et al., 2014). Nevertheless, the function of these DUF966 proteins has not been characterized further. In this study, our qRT-PCR data showed that three *TaDUF966* genes (*TaDUF966-5D*, *TaDUF966-9B*, and *TaDUF966-14D*) were up-regulated in the root and leaf tissues of wheat plants treated with NaCl, which is consistent with previous RNA-seq data (**Figure 5**). These results, together with phylogenetic analysis, strongly suggest that the DUF966 family members in wheat are also involved in salt stress tolerance.

To further verify the role of *TaDUF966* family genes in salt stress tolerance, we silenced one wheat gene (*TaDUF966-9B*) using VIGS and then treated the plants with NaCl. The expression of *TaDUF966-9B* was not induced upon NaCl treatment, indicating that *TaDUF966-9B* responds to salt stress in wheat. Although gene-specific primers were designed for *TaDUF966-9B*, and sequences of cDNA fragments cloned into VIGS vectors were confirmed by Sanger sequencing, the possibility of knocking down the expression of *TaDUF966-9B* homologs could not be completely ruled out. Therefore, further studies are needed to generate stable transgenic lines using the CRISPR/Cas technology to better understand the function of *TaDUF966* genes. In conclusion, our results provide a comprehensive analysis of the biological function of *DUF966* family genes in wheat.

## Data Availability Statement

All datasets presented in this study are included in the article/supplementary material.

## Author Contributions

XGZ, DM, and YQ guided the design of the experiment. XYZ, WS, YH, WJ, JS, and JY directed the data analysis. XYZ conducted data analysis and manuscript writing. XGZ, DM and YQ supervised the experiment and confirmed the manuscript. XGZ is the guarantor of this work, so she can have full access to all the data in the research and is responsible for the integrity of the data and the accuracy of the data analysis. All authors contributed to the article and approved the submitted version. Thank all the above staff for the help in this study. The authors thank the reviewers for their valuable suggestions during the revision of the early manuscripts.

## Funding

This research was funded by the National Key R&D Program of China, grant number 2018YFD0200506.

## Conflict of Interest

The authors declare that the research was conducted in the absence of any commercial or financial relationships that could be construed as a potential conflict of interest.
