# Supplementary material for: Genome-Wide Mining of Wheat DUF966 Gene Family Provides New Insights Into Salt Stress Responses
Source: Front Plant Sci. 2020 Aug 28;11:569838. doi: 10.3389/fpls.2020.569838 (PMC7483657; doi:10.3389/fpls.2020.569838)
Supplement: Supplementary file 1 [file Table_1.docx]

Supplementary Material
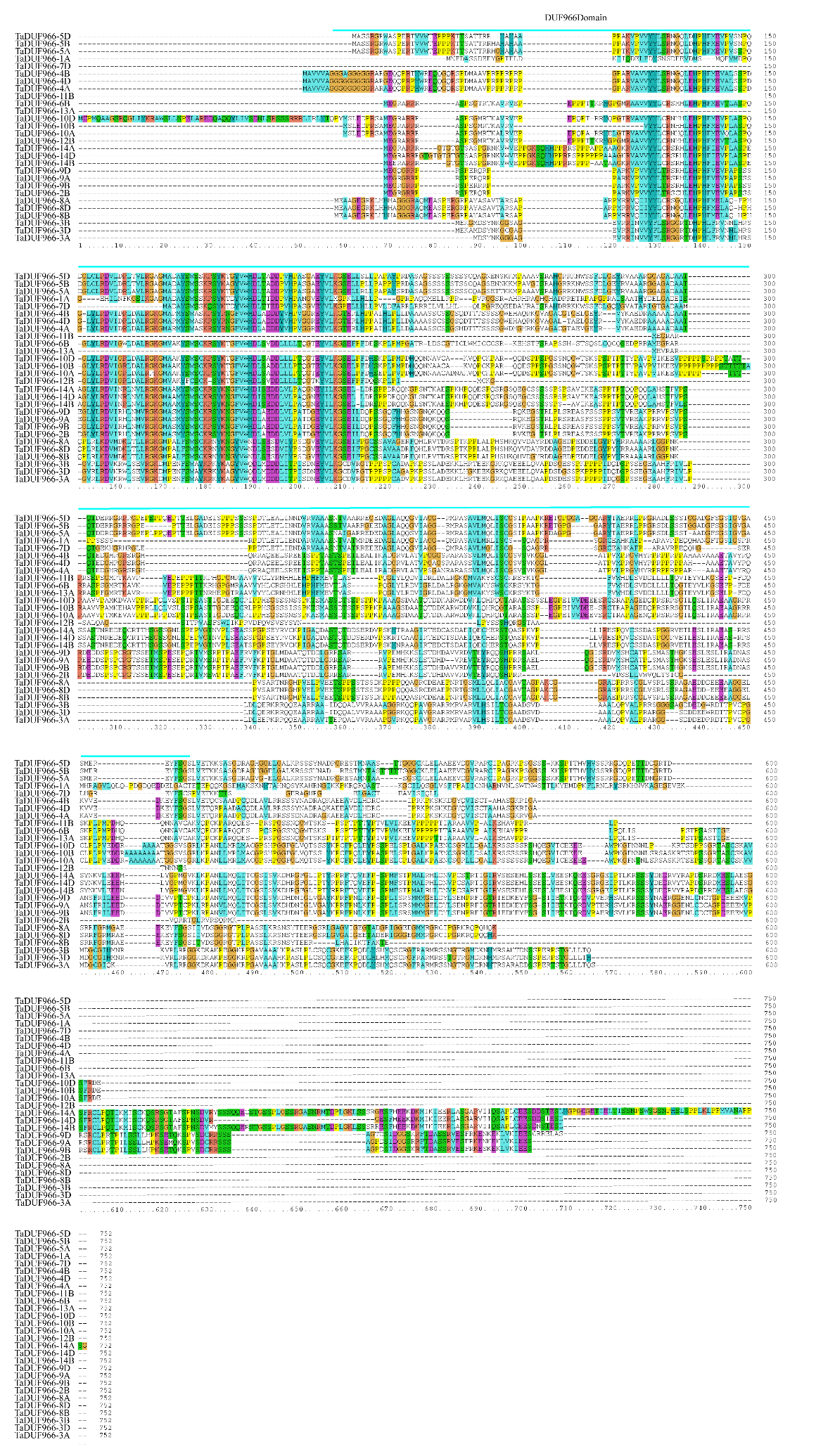


Supplementary Figure 1. | The DNA conserved domain sequence alignment of the TaDUF966 gene family members


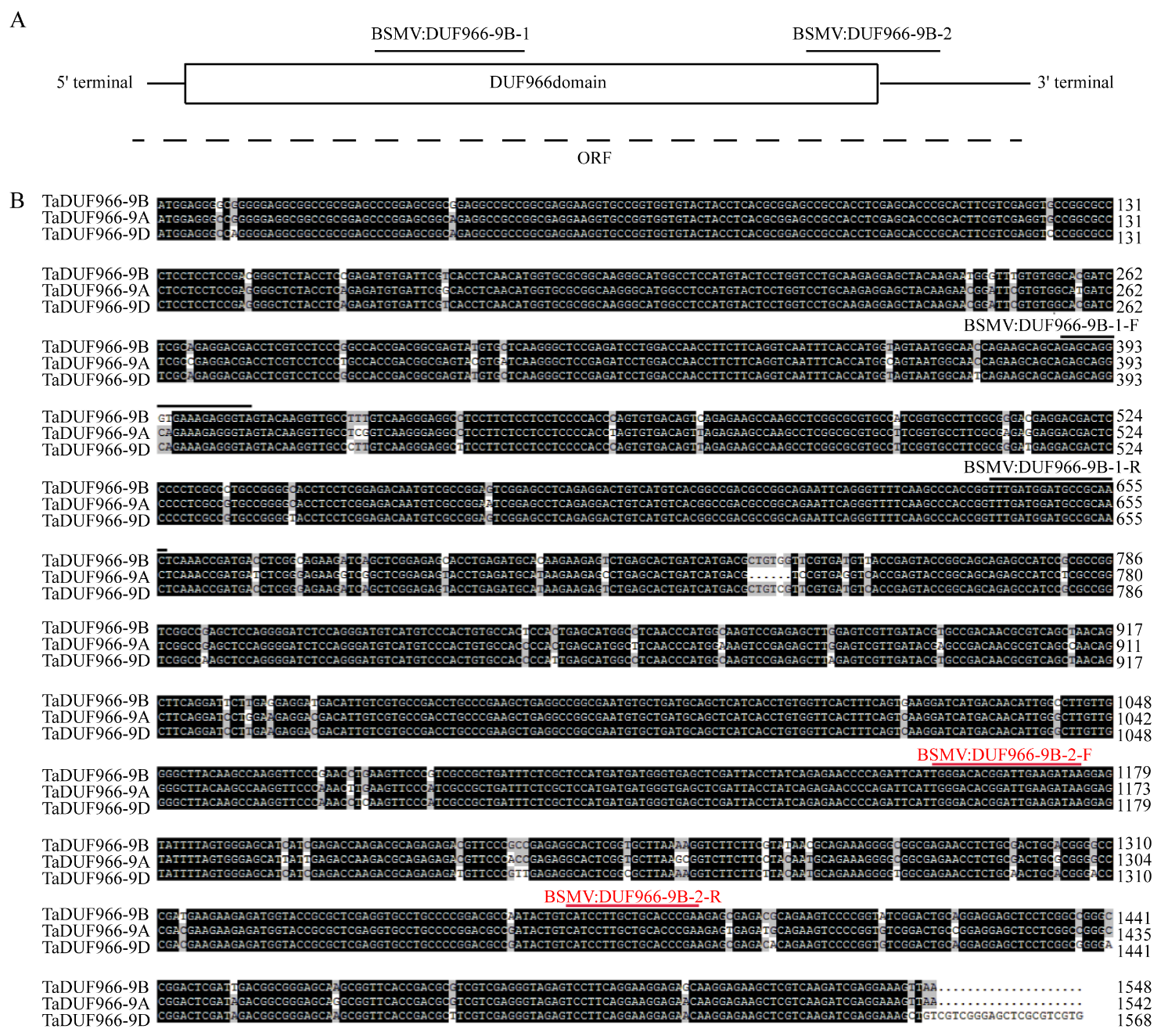


Supplementary Figure 2. TaDUF966-9B fragment was used for BSMV-HIGS analysis. (A) Overview of the two fragments used for BSMV-HIGS. (B) Sequence alignment of the positions of TaDUF966-9B and the primers used for HIGS.

Supplementary Table 1. | Primers for quantitative real-time PCR

| **Primer name** | **Primers (5’-3’)** |
| --- | --- |
| qPCR-TaDUF966-5D-F | GAGCTACAAGACCGGCTACG |
| qPCR-TaDUF966-5D-R | GAGGAGCCGGCGGAAGCGTCA |
| qPCR-TaDUF966-9B-F | GGCAGAATTCAGGGTTTTCA |
| qPCR-TaDUF966-9B-R | CTGCCGGTACTCGGTAACAT |
| qPCR-TaDUF966-14D-F | ATGGCCTTAGCAACACCAAG |
| qPCR-TaDUF966-14D-R | GATGGCACAAATGTGGAGTG |
| TaEF-F | TGGTGTCATCAAGCCTGGTATGGT |
| TaEF-R | ACTCATGGTGCATCTCAACGGACT |

Supplementary Table 2. | Primers for constructs in plant transformation

| **Primer name** | **Primers (5’-3’)** |
| --- | --- |
| BSMV:DUF966-9B-1-F | TAGCTAGCTGATTAATTAAGAGCAGGGTGAAAGAGGGTA |
| BSMV:DUF966-9B-1-R | TTGCTAGCTGAGCGGCCGCGTTGCGGCATCCATCAAA |
| BSMV:DUF966-9B-2-F | TAGCTAGCTGATTAATTAATGGGACACGGATTGAAGATAA |
| BSMV:DUF966-9B-2-R | TTGCTAGCTGAGCGGCCGCTCGGGTGCAGCAAGGATG |

Supplementary Table 3. | Primers for quantitative real-time PCR in plant transformation

| **Primer name** | **Primers (5’-3’)** |
| --- | --- |
| qPCR-TaDUF966-9B-F | TGTCCCACTGTGCCACTC |
| qPCR-TaDUF966-9B-R | CGATGATGCTCCCACTAAA |
| TaEF-F | TGGTGTCATCAAGCCTGGTATGGT |
| TaEF-R | ACTCATGGTGCATCTCAACGGACT |

Supplementary Table 4. | General information of DUF966 genes selected for phylogenetic analysis of Figure 1A

| **Gene** | **Locus ID** | **splice variant** | **Source of information** |
| --- | --- | --- | --- |
| OsDSR1 | LOC_Os01g57020 | 1 | <http://rice.plantbiology.msu.edu/index.shtml>  (Luo and Tian 2017) |
| OsDSR2 | LOC_Os01g62200 | 2 | <http://rice.plantbiology.msu.edu/index.shtml>  (Luo and Tian 2017) |
| OsDSR3 | LOC_Os01g74370 | 1 | <http://rice.plantbiology.msu.edu/index.shtml>  (Luo and Tian 2017) |
| OsDSR4 | LOC_Os03g09200 | 1 | <http://rice.plantbiology.msu.edu/index.shtml>  (Luo and Tian 2017) |
| OsDSR5 | LOC_Os03g48600 | 10 | <http://rice.plantbiology.msu.edu/index.shtml>  (Luo and Lian 2017) |
| OsDSR6 | LOC_Os08g44760 | 3 | <http://rice.plantbiology.msu.edu/index.shtml>  (Luo and Lian 2017) |
| OsDSR7 | LOC_Os09g39750 | 1 | <http://rice.plantbiology.msu.edu/index.shtml>  (Luo and Lian 2017) |
| AtUOF1 | AT1G05577 | 1 | https://www.arabidopsis.org/index.jsp |
| AtUOF2 | AT2G28150 | 3 | https://www.arabidopsis.org/index.jsp |
| AtUOF3 | AT3G46110 | 3 | https://www.arabidopsis.org/index.jsp |
| AtUOF4 | AT5G10150 | 4 | https://www.arabidopsis.org/index.jsp |
| AtUOF5 | AT5G59790 | 2 | https://www.arabidopsis.org/index.jsp |

Supplementary Table 5. | List of 1:1:1 High Confidence syntenic triads identified in this study

| Number | A | B | D |
| --- | --- | --- | --- |
| 1 | TraesCS3A02G318800 | TraesCS3B02G347200 | TraesCS3D02G312500 |
| 2 | TraesCS3A02G356700 | TraesCS3B02G389500 | TraesCS3D02G350800 |
| 3 | TraesCS3A02G535000 | TraesCS3B02G612100 | TraesCS3D02G540500 |
| 4 | TraesCS4A02G038000 | TraesCS4B02G267700 | TraesCS4D02G267300 |
| 5 | TraesCS4A02G280800 | TraesCS4B02G032000 | TraesCS4D02G029700 |
| 6 | TraesCS4A02G311800 | TraesCS4B02G000600 | TraesCS4D02G001100LC |
| 7 | TraesCS7A02G281800 | TraesCS7B02G179600 | TraesCS7D02G280300 |

Supplementary Table 6. | Homologous gene pairs of DUF966 genes

| **No** | **Gene pairs** | **Ka** | **Ks** | **Ka/Ks ratio** | **Replicative type** |
| --- | --- | --- | --- | --- | --- |
| 1 | TaDUF966-2B/TdDUF966-1B | 0.038490541 | 0.070050881 | 0.549465478 |  |
| 2 | TaDUF966-3A/TdDUF966-2A | 0.005837315 | 0.021764313 | 0.268205809 |  |
| 3 | TaDUF966-3A/TaDUF966-3D | 0.036075484 | 0.111780681 | 0.322734518 | Segmental duplication |
| 4 | TaDUF966-3A/TaDUF966-3B | 0.041656415 | 0.09724805 | 0.42835219 | Segmental duplication |
| 5 | TaDUF966-3A/TdDUF966-5B | 0.044134823 | 0.093246314 | 0.4733144 |  |
| 6 | TaDUF966-3B/TdDUF966-5B | 0.002304592 | 0.003582096 | 0.643363985 |  |
| 7 | TaDUF966-3B/TaDUF966-3D | 0.025633563 | 0.1118887 | 0.229098765 | Segmental duplication |
| 8 | TaDUF966-3D/TdDUF966-5B | 0.027141605 | 0.108506079 | 0.250139024 |  |
| 9 | TaDUF966-8A/TuDUF966-6A | 0.001126549 | 0.006703955 | 0.168042473 |  |
| 10 | TaDUF966-8A/TaDUF966-8D | 0.020584482 | 0.08880853 | 0.231784969 | Segmental duplication |
| 11 | TaDUF966-8A/AeDUF966-8D | 0.036546893 | 0.122465093 | 0.29842702 |  |
| 12 | TaDUF966-8A/TaDUF966-8B | 0.042047636 | 0.118068004 | 0.356130658 | Segmental duplication |
| 13 | TaDUF966-8A/TdDUF966-13B | 0.015381868 | 0.099681981 | 0.154309412 |  |
| 14 | TaDUF966-8B/TdDUF966-13B | 0.002541298 | 0.003745326 | 0.678525287 |  |
| 15 | TaDUF966-8B/TaDUF966-8D | 0.028803871 | 0.119944447 | 0.240143434 | Segmental duplication |
| 16 | TaDUF966-8B/AeDUF966-8D | 0.034977196 | 0.112033051 | 0.31220426 |  |
| 17 | TaDUF966-8B/TuDUF966-6A | 0.040764122 | 0.118068004 | 0.345259683 |  |
| 18 | TaDUF966-8D/AeDUF966-8D | 0.020075318 | 0.020771948 | 0.966462971 |  |
| 19 | TaDUF966-8D/TuDUF966-6A | 0.019425957 | 0.08880853 | 0.218739769 |  |
| 20 | TaDUF966-8D/TdDUF966-13B | 0.006392538 | 0.082924612 | 0.077088551 |  |
| 21 | TaDUF966-9A/TdDUF966-9A | 8.58E-04 | 0.002690586 | 0.318753266 |  |
| 22 | TaDUF966-9A/TuDUF966-5A | 0.002575847 | 0 | #NAME? |  |
| 23 | TaDUF966-9A/TaDUF966-9D | 0.013818311 | 0.126119327 | 0.109565372 | Segmental duplication |
| 24 | TaDUF966-9A/TaDUF966-9B | 0.012535412 | 0.159390181 | 0.078646073 | Segmental duplication |
| 25 | TaDUF966-9A/AeDUF966-7D | 0.04062594 | 0.167065209 | 0.24317415 |  |
| 26 | TaDUF966-9B/TaDUF966-9D | 0.010307965 | 0.128405896 | 0.080276413 | Segmental duplication |
| 27 | TaDUF966-9B/TdDUF966-9A | 0.011665822 | 0.162635805 | 0.071729726 |  |
| 28 | TaDUF966-9B/TuDUF966-5A | 0.013409628 | 0.159310779 | 0.084172763 |  |
| 29 | TaDUF966-9B/AeDUF966-7D | 0.042116031 | 0.159713385 | 0.263697566 |  |
| 30 | TaDUF966-9D/TdDUF966-9A | 0.01294903 | 0.129256712 | 0.100180719 |  |
| 31 | TaDUF966-9D/AeDUF966-7D | 0.030707022 | 0.03965095 | 0.774433446 |  |
| 32 | TaDUF966-10A/TdDUF966-10A | 8.82E-04 | 0 | #NAME? |  |
| 33 | TaDUF966-10A/TaDUF966-10D | 0.031941772 | 0.170537745 | 0.187300308 | Segmental duplication |
| 34 | TaDUF966-10A/TaDUF966-10B | 0.032125837 | 0.144066166 | 0.222993627 | Segmental duplication |
| 35 | TaDUF966-10D/TdDUF966-10A | 0.030996305 | 0.17062124 | 0.181667331 |  |
| 36 | TaDUF966-10D/TaDUF966-10B | 0.022320503 | 0.1475279 | 0.151296826 | Segmental duplication |
| 37 | TaDUF966-11B/TuDUF966-9A | 0.03808999 | 0.017929141 | 2.124473843 |  |
| 38 | TaDUF966-11B/TuDUF966-8A | 0.03808999 | 0.017929141 | 2.124473843 |  |
| 39 | TaDUF966-11B/TaDUF966-13A | 0.042414127 | 0.03018516 | 1.405131747 | Segmental duplication |
| 40 | TaDUF966-13A/TuDUF966-9A | 0.007802411 | 0.016514429 | 0.472460248 |  |
| 41 | TaDUF966-13A/TuDUF966-8A | 0.007802411 | 0.016514429 | 0.472460248 |  |
| 42 | TaDUF966-13A/TuDUF966-7A | 0.076937916 | 0.181305947 | 0.424354066 |  |
| 43 | TaDUF966-13A/TuDUF966-4A | 0.076937916 | 0.181305947 | 0.424354066 |  |
| 44 | TaDUF966-14A/TaDUF966-14B | 0.011208877 | 0.047250418 | 0.237222802 | Segmental duplication |
| 45 | TaDUF966-14A/TdDUF966-15B | 0.011962125 | 0.052143402 | 0.229408221 |  |
| 46 | TaDUF966-14A/TdDUF966-14A | 8.89E-04 | 0 | #NAME? |  |
| 47 | TaDUF966-14B/TdDUF966-15B | 0.002228276 | 0.004594195 | 0.485019885 |  |
| 48 | TaDUF966-14B/TaDUF966-14D | 0.011132667 | 0.055663049 | 0.200001034 | Segmental duplication |
| 49 | TaDUF966-14B/TdDUF966-14A | 0.011823876 | 0.04449279 | 0.26574813 |  |
| 50 | TaDUF966-14D/TaDUF966-14A | 0.007081555 | 0.02948179 | 0.240201004 | Segmental duplication |
| 51 | TaDUF966-14D/TdDUF966-15B | 0.011934217 | 0.060934321 | 0.195853782 |  |
| 52 | TdDUF966-2A/TaDUF966-3D | 0.032298569 | 0.121905107 | 0.264948447 |  |
| 53 | TdDUF966-2A/TaDUF966-3B | 0.04216047 | 0.108903357 | 0.387136553 |  |
| 54 | TdDUF966-2A/TdDUF966-5B | 0.043662142 | 0.104092869 | 0.41945373 | Segmental duplication |
| 55 | TdDUF966-9A/TuDUF966-5A | 0.001716493 | 0.00268938 | 0.638248772 |  |
| 56 | TdDUF966-10A/TaDUF966-10B | 0.031156147 | 0.144137014 | 0.216156465 |  |
| 57 | TdDUF966-13B/TuDUF966-6A | 0.014087928 | 0.099681981 | 0.141328727 |  |
| 58 | TdDUF966-14A/TdDUF966-15B | 0.012817665 | 0.051069976 | 0.250982389 | Segmental duplication |
| 59 | TuDUF966-1A/TuDUF966-2A | 0 | 0 | NaN | Tandem duplication |
| 60 | TuDUF966-4A/TuDUF966-7A | 0 | 0 | NaN | Tandem duplication |
| 61 | TuDUF966-4A/TuDUF966-9A | 0.068391453 | 0.167187383 | 0.409070659 | Segmental duplication |
| 62 | TuDUF966-4A/TuDUF966-8A | 0.068391453 | 0.167187383 | 0.409070659 | Segmental duplication |
| 63 | TuDUF966-5A/TaDUF966-9D | 0.014692584 | 0.122871997 | 0.11957634 |  |
| 64 | TuDUF966-7A/TuDUF966-9A | 0.068391453 | 0.167187383 | 0.409070659 | Segmental duplication |
| 65 | TuDUF966-7A/TuDUF966-8A | 0.068391453 | 0.167187383 | 0.409070659 | Segmental duplication |
| 66 | TuDUF966-8A/TuDUF966-9A | 0 | 0 | NaN | Tandem duplication |
| 67 | AeDUF966-3D/TdDUF966-2A | 0.031439316 | 0.110266453 | 0.285121311 |  |
| 68 | AeDUF966-6D/TdDUF966-11B | 0.028727204 | 0.149689619 | 0.1919118 |  |
| 69 | AeDUF966-7D/TdDUF966-9A | 0.039589704 | 0.166967952 | 0.2371096 |  |
| 70 | AeDUF966-7D/TuDUF966-5A | 0.041677388 | 0.163081933 | 0.25556104 |  |
| 71 | AeDUF966-8D/TuDUF966-6A | 0.035267575 | 0.122465093 | 0.287980631 |  |
| 72 | AeDUF966-8D/TdDUF966-13B | 0.006392538 | 0.082924612 | 0.077088551 |  |

Supplementary Table 7. | Motif sequences identified by MEME tools

| **Motif** | **Domain** | **Number of amino acid** | **Best possible match** |
| --- | --- | --- | --- |
| Motif 1 | DUF966 | 30 | SYKNGFVWHDLSEDDLVLPAQGTEYVLKGS |
| Motif 2 | DUF966 | 29 | QGLYLRDVIDRLBALRGKGMAAMYSWSCK |
| Motif 3 | DUF966 | 41 | RAPPPPTRPAPPGRRVAVVYYLCRNGHLEHPHFMEVPLASP |
| Motif 4 | DUF966 | 29 | TGVPAGGRLRPANVLMQLITCGSVSVKDA |
| Motif 5 | DUF966 | 41 | RFPNLEFPSPLFSTPMALGELDYLPENPRTIGTRIEDKEHL |
| Motif 6 | unknown | 21 | AVAKPAAGADAATQTDDKGRR |
| Motif 7 | unknown | 29 | ELPFDHSKPLPMPDHQQNNAVCAKVQPCK |
| Motif 8 | unknown | 50 | REAKPRRVPSVPSRPZDDSPSPCRGTSSETMSPESEPQRTVMSGPTPAE |
| Motif 9 | DUF966 | 21 | EISPPSSSGRPETLESLIRAD |
| Motif 10 | DUF966 | 21 | RGELSALKRSSSYNADRGGEA |

Supplementary Table 8. | Predicted sequence features of TaDUF966 proteins.

| **Common  name** | **Molecular  weight(D)** | **Isoelectric  point** | **Instability  index** | **Predicted  location(s)** |
| --- | --- | --- | --- | --- |
| TaDUF966-1A | 44445.46 | 7 | 54.85 | Cytoplasm |
| TaDUF966-2B | 33087.26 | 9.88 | 79.99 | Nucleus |
| TaDUF966-3A | 41787.11 | 9.72 | 57.87 | Nucleus |
| TaDUF966-3B | 42571.15 | 9.77 | 60.43 | Nucleus |
| TaDUF966-3D | 42427.17 | 9.73 | 56.53 | Nucleus |
| TaDUF966-4A | 43560.64 | 9.49 | 47.44 | Chloroplast /Nucleus |
| TaDUF966-4B | 43742.93 | 9.33 | 46.49 | Chloroplast |
| TaDUF966-4D | 43572.77 | 9.51 | 49.17 | Chloroplast /Nucleus |
| TaDUF966-5A | 47829.85 | 9.16 | 58.23 | Nucleus |
| TaDUF966-5B | 47640.58 | 9.22 | 54.91 | Chloroplast /Nucleus |
| TaDUF966-5D | 48065.98 | 9.22 | 55.09 | Nucleus |
| TaDUF966-6B | 46564.4 | 9.34 | 64.48 | Nucleus |
| TaDUF966-7D | 28290.79 | 8.35 | 57.89 | Nucleus |
| TaDUF966-8A | 42968.06 | 8.7 | 56.75 | Nucleus |
| TaDUF966-8B | 40174.87 | 8.12 | 58.77 | Nucleus |
| TaDUF966-8D | 43174.27 | 8.7 | 58.54 | Nucleus |
| TaDUF966-9A | 57592.67 | 9.02 | 77.06 | Chloroplast /Nucleus |
| TaDUF966-9B | 57563.65 | 9.01 | 76.04 | Nucleus |
| TaDUF966-9D | 58423.65 | 9.11 | 75.49 | Nucleus |
| TaDUF966-10A | 54683.24 | 8.72 | 78.74 | Nucleus |
| TaDUF966-10B | 52816.26 | 9.08 | 78.08 | Nucleus |
| TaDUF966-10D | 61250.84 | 9.31 | 78.12 | Nucleus |
| TaDUF966-11B | 24228.92 | 9.53 | 69.89 | Nucleus |
| TaDUF966-12B | 21298.42 | 9.86 | 36.76 | Chloroplast |
| TaDUF966-13A | 25991.82 | 9.54 | 59.08 | Nucleus |
| TaDUF966-14A | 69840.59 | 6.82 | 75.5 | Nucleus |
| TaDUF966-14B | 65223.49 | 7.23 | 77.78 | Nucleus |
| TaDUF966-14D | 62017.04 | 6.46 | 77.43 | Nucleus |

## Supplementary Table 9. | Detailed information on predicted *C*is-Acting Elements

| **Number** | **Name** | **types** | **function** |
| --- | --- | --- | --- |
| 1 | AE-box | Biotic/abiotic stress | part of a module for light response |
| 2 | ARE | Biotic/abiotic stress | cis-acting regulatory element essential for the anaerobic induction |
| 3 | Box 4 | Biotic/abiotic stress | part of a conserved DNA module involved in light responsiveness |
| 4 | GATA-motif | Biotic/abiotic stress | part of a light responsive element |
| 5 | G-Box | Biotic/abiotic stress | cis-acting regulatory element involved in light responsiveness |
| 6 | GC-motif | Biotic/abiotic stress | enhancer-like element involved in anoxic specific inducibility |
| 7 | GT1-motif | Biotic/abiotic stress | light responsive element |
| 8 | I-box | Biotic/abiotic stress | part of a light responsive element |
| 9 | LTR | Biotic/abiotic stress | cis-acting element involved in low-temperature responsiveness |
| 10 | MBS | Biotic/abiotic stress | MYB binding site involved in drought-inducibility |
| 11 | MRE | Biotic/abiotic stress | MYB binding site involved in light responsiveness |
| 12 | Sp1 | Biotic/abiotic stress | light responsive element |
| 13 | TC-rich repeats | Biotic/abiotic stress | cis-acting element involved in defense and stress responsiveness |
| 14 | TCT-motif | Biotic/abiotic stress | part of a light responsive element |
| 15 | A-box | Growth and development | cis-acting regulatory element |
| 16 | CAT-box | Growth and development | cis-acting regulatory element related to meristem expression |
| 17 | CAAT-box | Growth and development | common cis-acting element in promoter and enhancer regions |
| 18 | CCAAT-box | Growth and development | MYBHv1 binding site |
| 19 | O2-site | Growth and development | cis-acting regulatory element involved in zein metabolism regulation |
| 20 | TATA-box | Growth and development | core promoter element around -30 of transcription start |
| 21 | ABRE | Phytohormone response | cis-acting element involved in the abscisic acid responsiveness |
| 22 | CGTCA-motif | Phytohormone response | cis-acting regulatory element involved in the MeJA-responsiveness |
| 23 | P-box | Phytohormone response | gibberellin-responsive element |
| 24 | TCA-element | Phytohormone response | cis-acting element involved in salicylic acid responsiveness |
| 25 | TGACG-motif | Phytohormone response | cis-acting regulatory element involved in the MeJA-responsiveness |
| 26 | TGA-element | Phytohormone response | auxin-responsive element |

**Supplementary Table 10. |** Prediction results about cis-acting elements by PlantCARE analysis.

| **Gene** | | **Name** | | **Coding sequence** | | **Start position** | | **End position** | | **Code length** | |
| --- | --- | --- | --- | --- | --- | --- | --- | --- | --- | --- | --- |
| TaDUF966-1A | ABRE | | ACGTG | | 445 | | 450 | | 5 | |  |
| TaDUF966-1A | AE-box | | AGAAACAA | | 26 | | 34 | | 8 | |  |
| TaDUF966-1A | ARE | | AAACCA | | 900 | | 906 | | 6 | |  |
| TaDUF966-1A | ARE | | AAACCA | | 1275 | | 1281 | | 6 | |  |
| TaDUF966-1A | CAAT-box | | CAAT | | 49 | | 53 | | 4 | |  |
| TaDUF966-1A | CAAT-box | | CAAT | | 97 | | 101 | | 4 | |  |
| TaDUF966-1A | CAAT-box | | CCAAT | | 387 | | 392 | | 5 | |  |
| TaDUF966-1A | CAAT-box | | CAAT | | 388 | | 392 | | 4 | |  |
| TaDUF966-1A | CAAT-box | | CCAAT | | 467 | | 472 | | 5 | |  |
| TaDUF966-1A | CAAT-box | | CAAT | | 526 | | 530 | | 4 | |  |
| TaDUF966-1A | CAAT-box | | CCAAT | | 587 | | 592 | | 5 | |  |
| TaDUF966-1A | CAAT-box | | CAAAT | | 603 | | 608 | | 5 | |  |
| TaDUF966-1A | CAAT-box | | CAAAT | | 635 | | 640 | | 5 | |  |
| TaDUF966-1A | CAAT-box | | CAAT | | 844 | | 848 | | 4 | |  |
| TaDUF966-1A | CAAT-box | | CAAT | | 891 | | 895 | | 4 | |  |
| TaDUF966-1A | CAAT-box | | CCAAT | | 993 | | 998 | | 5 | |  |
| TaDUF966-1A | CAAT-box | | CAAAT | | 1049 | | 1054 | | 5 | |  |
| TaDUF966-1A | CAAT-box | | CAAAT | | 1113 | | 1118 | | 5 | |  |
| TaDUF966-1A | CAAT-box | | CAAAT | | 1144 | | 1149 | | 5 | |  |
| TaDUF966-1A | CAAT-box | | CAAT | | 1232 | | 1236 | | 4 | |  |
| TaDUF966-1A | CAAT-box | | CCAAT | | 1248 | | 1253 | | 5 | |  |
| TaDUF966-1A | CAAT-box | | CAAT | | 1249 | | 1253 | | 4 | |  |
| TaDUF966-1A | CAAT-box | | CAAAT | | 1284 | | 1289 | | 5 | |  |
| TaDUF966-1A | CAAT-box | | CAAT | | 1297 | | 1301 | | 4 | |  |
| TaDUF966-1A | CAAT-box | | CAAT | | 1307 | | 1311 | | 4 | |  |
| TaDUF966-1A | CAAT-box | | CAAAT | | 1357 | | 1362 | | 5 | |  |
| TaDUF966-1A | CAAT-box | | CCAAT | | 1369 | | 1374 | | 5 | |  |
| TaDUF966-1A | CAAT-box | | CAAT | | 1370 | | 1374 | | 4 | |  |
| TaDUF966-1A | CAT-box | | GCCACT | | 1158 | | 1164 | | 6 | |  |
| TaDUF966-1A | CGTCA-motif | | CGTCA | | 447 | | 452 | | 5 | |  |
| TaDUF966-1A | G-box | | CACGTC | | 445 | | 451 | | 6 | |  |
| TaDUF966-1A | LTR | | CCGAAA | | 515 | | 521 | | 6 | |  |
| TaDUF966-1A | MBS | | CAACTG | | 804 | | 810 | | 6 | |  |
| TaDUF966-1A | MBS | | CAACTG | | 869 | | 875 | | 6 | |  |
| TaDUF966-1A | TATA-box | | TATAAGAA | | 323 | | 331 | | 8 | |  |
| TaDUF966-1A | TATA-box | | TATAA | | 326 | | 331 | | 5 | |  |
| TaDUF966-1A | TATA-box | | TATA | | 327 | | 331 | | 4 | |  |
| TaDUF966-1A | TATA-box | | TATAAA | | 422 | | 428 | | 6 | |  |
| TaDUF966-1A | TATA-box | | TATAA | | 423 | | 428 | | 5 | |  |
| TaDUF966-1A | TATA-box | | TATA | | 424 | | 428 | | 4 | |  |
| TaDUF966-1A | TATA-box | | TATA | | 1257 | | 1261 | | 4 | |  |
| TaDUF966-1A | TATA-box | | TATAAAA | | 1326 | | 1333 | | 7 | |  |
| TaDUF966-1A | TATA-box | | TATAAA | | 1327 | | 1333 | | 6 | |  |
| TaDUF966-1A | TATA-box | | TATAA | | 1328 | | 1333 | | 5 | |  |
| TaDUF966-1A | TATA-box | | TATA | | 1329 | | 1333 | | 4 | |  |
| TaDUF966-1A | TCA-element | | TCAGAAGAGG | | 4 | | 13 | | 9 | |  |
| TaDUF966-1A | TCT-motif | | TCTTAC | | 1074 | | 1080 | | 6 | |  |
| TaDUF966-1A | TGACG-motif | | TGACG | | 447 | | 452 | | 5 | |  |
| TaDUF966-1A | TGA-element | | AACGAC | | 1398 | | 1404 | | 6 | |  |
| TaDUF966-2 | A-box | | CCGTCC | | 510 | | 516 | | 6 | |  |
| TaDUF966-2 | A-box | | CCGTCC | | 1259 | | 1265 | | 6 | |  |
| TaDUF966-2 | A-box | | CCGTCC | | 1314 | | 1320 | | 6 | |  |
| TaDUF966-2 | ABRE | | GCCGCGTGGC | | 688 | | 697 | | 9 | |  |
| TaDUF966-2 | CAAT-box | | CAAAT | | 201 | | 206 | | 5 | |  |
| TaDUF966-2 | CAAT-box | | CAAT | | 296 | | 300 | | 4 | |  |
| TaDUF966-2 | CAAT-box | | CAAT | | 489 | | 493 | | 4 | |  |
| TaDUF966-2 | CAAT-box | | CAAT | | 627 | | 631 | | 4 | |  |
| TaDUF966-2 | CAAT-box | | CAAAT | | 912 | | 917 | | 5 | |  |
| TaDUF966-2 | CAAT-box | | CAAAT | | 988 | | 993 | | 5 | |  |
| TaDUF966-2 | CAAT-box | | CAAAT | | 1029 | | 1034 | | 5 | |  |
| TaDUF966-2 | CAAT-box | | CAAT | | 1038 | | 1042 | | 4 | |  |
| TaDUF966-2 | CAAT-box | | CAAAT | | 1112 | | 1117 | | 5 | |  |
| TaDUF966-2 | CAAT-box | | CAAAT | | 1159 | | 1164 | | 5 | |  |
| TaDUF966-2 | CAAT-box | | CAAT | | 1408 | | 1412 | | 4 | |  |
| TaDUF966-2 | CAAT-box | | CAAT | | 1457 | | 1461 | | 4 | |  |
| TaDUF966-2 | CAT-box | | GCCACT | | 221 | | 227 | | 6 | |  |
| TaDUF966-2 | CAT-box | | GCCACT | | 677 | | 683 | | 6 | |  |
| TaDUF966-2 | CAT-box | | GCCACT | | 871 | | 877 | | 6 | |  |
| TaDUF966-2 | CCAAT-box | | CAACGG | | 525 | | 531 | | 6 | |  |
| TaDUF966-2 | CGTCA-motif | | CGTCA | | 119 | | 124 | | 5 | |  |
| TaDUF966-2 | CGTCA-motif | | CGTCA | | 339 | | 344 | | 5 | |  |
| TaDUF966-2 | CGTCA-motif | | CGTCA | | 641 | | 646 | | 5 | |  |
| TaDUF966-2 | CGTCA-motif | | CGTCA | | 1410 | | 1415 | | 5 | |  |
| TaDUF966-2 | G-box | | TAACACGTAG | | 29 | | 38 | | 9 | |  |
| TaDUF966-2 | G-box | | CACGAC | | 360 | | 366 | | 6 | |  |
| TaDUF966-2 | GC-motif | | CCCCCG | | 585 | | 591 | | 6 | |  |
| TaDUF966-2 | GC-motif | | CCCCCG | | 1468 | | 1474 | | 6 | |  |
| TaDUF966-2 | GC-motif | | CCCCCG | | 1493 | | 1499 | | 6 | |  |
| TaDUF966-2 | MBS | | CAACTG | | 451 | | 457 | | 6 | |  |
| TaDUF966-2 | Sp1 | | GGGCGG | | 160 | | 166 | | 6 | |  |
| TaDUF966-2 | Sp1 | | GGGCGG | | 685 | | 691 | | 6 | |  |
| TaDUF966-2 | Sp1 | | GGGCGG | | 735 | | 741 | | 6 | |  |
| TaDUF966-2 | Sp1 | | GGGCGG | | 769 | | 775 | | 6 | |  |
| TaDUF966-2 | Sp1 | | GGGCGG | | 1465 | | 1471 | | 6 | |  |
| TaDUF966-2 | TATA-box | | TATATA | | 1103 | | 1109 | | 6 | |  |
| TaDUF966-2 | TATA-box | | ATATAA | | 1104 | | 1110 | | 6 | |  |
| TaDUF966-2 | TATA-box | | TATA | | 1105 | | 1109 | | 4 | |  |
| TaDUF966-2 | TATA-box | | TATACA | | 1149 | | 1155 | | 6 | |  |
| TaDUF966-2 | TATA-box | | TATA | | 1151 | | 1155 | | 4 | |  |
| TaDUF966-2 | TGACG-motif | | TGACG | | 119 | | 124 | | 5 | |  |
| TaDUF966-2 | TGACG-motif | | TGACG | | 339 | | 344 | | 5 | |  |
| TaDUF966-2 | TGACG-motif | | TGACG | | 641 | | 646 | | 5 | |  |
| TaDUF966-2 | TGACG-motif | | TGACG | | 1410 | | 1415 | | 5 | |  |
| TaDUF966-3A | ABRE | | ACGTG | | 148 | | 153 | | 5 | |  |
| TaDUF966-3A | ABRE | | ACGTG | | 304 | | 309 | | 5 | |  |
| TaDUF966-3A | ABRE | | ACGTG | | 373 | | 378 | | 5 | |  |
| TaDUF966-3A | ABRE | | CACGTG | | 623 | | 629 | | 6 | |  |
| TaDUF966-3A | ABRE | | ACGTG | | 624 | | 629 | | 5 | |  |
| TaDUF966-3A | AE-box | | AGAAACAA | | 702 | | 710 | | 8 | |  |
| TaDUF966-3A | ARE | | AAACCA | | 682 | | 688 | | 6 | |  |
| TaDUF966-3A | ARE | | AAACCA | | 754 | | 760 | | 6 | |  |
| TaDUF966-3A | Box 4 | | ATTAAT | | 121 | | 127 | | 6 | |  |
| TaDUF966-3A | Box 4 | | ATTAAT | | 346 | | 352 | | 6 | |  |
| TaDUF966-3A | CAAT-box | | CAAAT | | 245 | | 250 | | 5 | |  |
| TaDUF966-3A | CAAT-box | | CAAAT | | 333 | | 338 | | 5 | |  |
| TaDUF966-3A | CAAT-box | | CAAT | | 368 | | 372 | | 4 | |  |
| TaDUF966-3A | CAAT-box | | CAAT | | 386 | | 390 | | 4 | |  |
| TaDUF966-3A | CAAT-box | | CAAAT | | 515 | | 520 | | 5 | |  |
| TaDUF966-3A | CAAT-box | | CAAAT | | 586 | | 591 | | 5 | |  |
| TaDUF966-3A | CAAT-box | | CAAT | | 615 | | 619 | | 4 | |  |
| TaDUF966-3A | CAAT-box | | CAAT | | 638 | | 642 | | 4 | |  |
| TaDUF966-3A | CAAT-box | | CAAT | | 645 | | 649 | | 4 | |  |
| TaDUF966-3A | CAAT-box | | CAAT | | 1211 | | 1215 | | 4 | |  |
| TaDUF966-3A | CAAT-box | | CAAAT | | 1356 | | 1361 | | 5 | |  |
| TaDUF966-3A | CAAT-box | | CAAAT | | 1372 | | 1377 | | 5 | |  |
| TaDUF966-3A | CAAT-box | | CAAT | | 1467 | | 1471 | | 4 | |  |
| TaDUF966-3A | CAT-box | | GCCACT | | 1222 | | 1228 | | 6 | |  |
| TaDUF966-3A | G-box | | TAAACGTG | | 145 | | 153 | | 8 | |  |
| TaDUF966-3A | G-box | | CACGTG | | 623 | | 629 | | 6 | |  |
| TaDUF966-3A | G-Box | | CACGTT | | 147 | | 153 | | 6 | |  |
| TaDUF966-3A | G-Box | | CACGTT | | 303 | | 309 | | 6 | |  |
| TaDUF966-3A | G-Box | | CACGTT | | 372 | | 378 | | 6 | |  |
| TaDUF966-3A | G-Box | | CACGTGAAA | | 620 | | 629 | | 9 | |  |
| TaDUF966-3A | G-Box | | CACGTG | | 623 | | 629 | | 6 | |  |
| TaDUF966-3A | LTR | | CCGAAA | | 725 | | 731 | | 6 | |  |
| TaDUF966-3A | Sp1 | | GGGCGG | | 1341 | | 1347 | | 6 | |  |
| TaDUF966-3A | TATA-box | | TATACA | | 38 | | 44 | | 6 | |  |
| TaDUF966-3A | TATA-box | | TATA | | 40 | | 44 | | 4 | |  |
| TaDUF966-3A | TATA-box | | TATACA | | 58 | | 64 | | 6 | |  |
| TaDUF966-3A | TATA-box | | TATA | | 60 | | 64 | | 4 | |  |
| TaDUF966-3A | TATA-box | | TATACA | | 151 | | 157 | | 6 | |  |
| TaDUF966-3A | TATA-box | | TATA | | 153 | | 157 | | 4 | |  |
| TaDUF966-3A | TATA-box | | TATACA | | 172 | | 178 | | 6 | |  |
| TaDUF966-3A | TATA-box | | TATA | | 174 | | 178 | | 4 | |  |
| TaDUF966-3A | TATA-box | | TATATA | | 219 | | 225 | | 6 | |  |
| TaDUF966-3A | TATA-box | | TATA | | 221 | | 225 | | 4 | |  |
| TaDUF966-3A | TATA-box | | TATATA | | 263 | | 269 | | 6 | |  |
| TaDUF966-3A | TATA-box | | ATATAT | | 264 | | 270 | | 6 | |  |
| TaDUF966-3A | TATA-box | | TATATA | | 265 | | 271 | | 6 | |  |
| TaDUF966-3A | TATA-box | | ATATAA | | 266 | | 272 | | 6 | |  |
| TaDUF966-3A | TATA-box | | TATA | | 267 | | 271 | | 4 | |  |
| TaDUF966-3A | TATA-box | | TATACA | | 285 | | 291 | | 6 | |  |
| TaDUF966-3A | TATA-box | | TATA | | 287 | | 291 | | 4 | |  |
| TaDUF966-3A | TATA-box | | TATTTAAA | | 356 | | 364 | | 8 | |  |
| TaDUF966-3A | TATA-box | | TATACA | | 397 | | 403 | | 6 | |  |
| TaDUF966-3A | TATA-box | | TATA | | 399 | | 403 | | 4 | |  |
| TaDUF966-3A | TATA-box | | TATACA | | 419 | | 425 | | 6 | |  |
| TaDUF966-3A | TATA-box | | TATA | | 421 | | 425 | | 4 | |  |
| TaDUF966-3A | TATA-box | | ATATAT | | 447 | | 453 | | 6 | |  |
| TaDUF966-3A | TATA-box | | TATA | | 448 | | 452 | | 4 | |  |
| TaDUF966-3A | TATA-box | | TATA | | 492 | | 496 | | 4 | |  |
| TaDUF966-3A | TATA-box | | TATACA | | 640 | | 646 | | 6 | |  |
| TaDUF966-3A | TATA-box | | TATA | | 642 | | 646 | | 4 | |  |
| TaDUF966-3A | TATA-box | | TATA | | 1199 | | 1203 | | 4 | |  |
| TaDUF966-3A | TATA-box | | ATATAT | | 1293 | | 1299 | | 6 | |  |
| TaDUF966-3A | TATA-box | | TATATA | | 1294 | | 1300 | | 6 | |  |
| TaDUF966-3A | TATA-box | | TATA | | 1296 | | 1300 | | 4 | |  |
| TaDUF966-3A | TATA-box | | TATATA | | 1303 | | 1309 | | 6 | |  |
| TaDUF966-3A | TATA-box | | ATATAA | | 1304 | | 1310 | | 6 | |  |
| TaDUF966-3A | TATA-box | | TATA | | 1305 | | 1309 | | 4 | |  |
| TaDUF966-3B | A-box | | CCGTCC | | 296 | | 302 | | 6 | |  |
| TaDUF966-3B | A-box | | CCGTCC | | 1059 | | 1065 | | 6 | |  |
| TaDUF966-3B | ARE | | AAACCA | | 230 | | 236 | | 6 | |  |
| TaDUF966-3B | ARE | | AAACCA | | 276 | | 282 | | 6 | |  |
| TaDUF966-3B | ARE | | AAACCA | | 1260 | | 1266 | | 6 | |  |
| TaDUF966-3B | CAAT-box | | CAAT | | 3 | | 7 | | 4 | |  |
| TaDUF966-3B | CAAT-box | | CAAT | | 56 | | 60 | | 4 | |  |
| TaDUF966-3B | CAAT-box | | CAAT | | 202 | | 206 | | 4 | |  |
| TaDUF966-3B | CAAT-box | | CCAAT | | 279 | | 284 | | 5 | |  |
| TaDUF966-3B | CAAT-box | | CAAT | | 280 | | 284 | | 4 | |  |
| TaDUF966-3B | CAAT-box | | CAAAT | | 337 | | 342 | | 5 | |  |
| TaDUF966-3B | CAAT-box | | CAAAT | | 353 | | 358 | | 5 | |  |
| TaDUF966-3B | CAAT-box | | CAAAT | | 359 | | 364 | | 5 | |  |
| TaDUF966-3B | CAAT-box | | CAAT | | 812 | | 816 | | 4 | |  |
| TaDUF966-3B | CAAT-box | | CCAAT | | 847 | | 852 | | 5 | |  |
| TaDUF966-3B | CAAT-box | | CAAAT | | 945 | | 950 | | 5 | |  |
| TaDUF966-3B | CAAT-box | | CAAT | | 958 | | 962 | | 4 | |  |
| TaDUF966-3B | CAAT-box | | CAAAT | | 986 | | 991 | | 5 | |  |
| TaDUF966-3B | CAAT-box | | CAAT | | 1125 | | 1129 | | 4 | |  |
| TaDUF966-3B | CAAT-box | | CCAAT | | 1127 | | 1132 | | 5 | |  |
| TaDUF966-3B | CAAT-box | | CAAT | | 1173 | | 1177 | | 4 | |  |
| TaDUF966-3B | CAAT-box | | CAAT | | 1178 | | 1182 | | 4 | |  |
| TaDUF966-3B | CAAT-box | | CAAT | | 1183 | | 1187 | | 4 | |  |
| TaDUF966-3B | CAAT-box | | CAAT | | 1214 | | 1218 | | 4 | |  |
| TaDUF966-3B | CAAT-box | | CAAT | | 1489 | | 1493 | | 4 | |  |
| TaDUF966-3B | CGTCA-motif | | CGTCA | | 306 | | 311 | | 5 | |  |
| TaDUF966-3B | GATA-motif | | GATAGGA | | 34 | | 41 | | 7 | |  |
| TaDUF966-3B | G-Box | | TCCACATGGCA | | 583 | | 593 | | 10 | |  |
| TaDUF966-3B | G-box | | GCCACGTGGA | | 583 | | 592 | | 9 | |  |
| TaDUF966-3B | GT1-motif | | GGTTAAT | | 1205 | | 1212 | | 7 | |  |
| TaDUF966-3B | I-box | | GTATAAGGCC | | 411 | | 420 | | 9 | |  |
| TaDUF966-3B | LTR | | CCGAAA | | 113 | | 119 | | 6 | |  |
| TaDUF966-3B | LTR | | CCGAAA | | 1016 | | 1022 | | 6 | |  |
| TaDUF966-3B | LTR | | CCGAAA | | 1027 | | 1033 | | 6 | |  |
| TaDUF966-3B | MBS | | CAACTG | | 861 | | 867 | | 6 | |  |
| TaDUF966-3B | MBS | | CAACTG | | 889 | | 895 | | 6 | |  |
| TaDUF966-3B | TATA-box | | TATAAATA | | 87 | | 95 | | 8 | |  |
| TaDUF966-3B | TATA-box | | TATAAAT | | 88 | | 95 | | 7 | |  |
| TaDUF966-3B | TATA-box | | TATAAA | | 89 | | 95 | | 6 | |  |
| TaDUF966-3B | TATA-box | | TATAA | | 90 | | 95 | | 5 | |  |
| TaDUF966-3B | TATA-box | | TATA | | 91 | | 95 | | 4 | |  |
| TaDUF966-3B | TATA-box | | TATAAA | | 164 | | 170 | | 6 | |  |
| TaDUF966-3B | TATA-box | | TATAA | | 165 | | 170 | | 5 | |  |
| TaDUF966-3B | TATA-box | | TATA | | 166 | | 170 | | 4 | |  |
| TaDUF966-3B | TATA-box | | TATAA | | 219 | | 224 | | 5 | |  |
| TaDUF966-3B | TATA-box | | TATA | | 220 | | 224 | | 4 | |  |
| TaDUF966-3B | TATA-box | | ATATAA | | 716 | | 722 | | 6 | |  |
| TaDUF966-3B | TATA-box | | TATA | | 717 | | 721 | | 4 | |  |
| TaDUF966-3B | TATA-box | | TATAA | | 759 | | 764 | | 5 | |  |
| TaDUF966-3B | TATA-box | | TATA | | 760 | | 764 | | 4 | |  |
| TaDUF966-3B | TATA-box | | ATTATA | | 1339 | | 1345 | | 6 | |  |
| TaDUF966-3B | TATA-box | | TATAA | | 1340 | | 1345 | | 5 | |  |
| TaDUF966-3B | TATA-box | | TATA | | 1341 | | 1345 | | 4 | |  |
| TaDUF966-3B | TATA-box | | ATATAA | | 1468 | | 1474 | | 6 | |  |
| TaDUF966-3B | TATA-box | | TATA | | 1469 | | 1473 | | 4 | |  |
| TaDUF966-3B | TCT-motif | | TCTTAC | | 909 | | 915 | | 6 | |  |
| TaDUF966-3B | TGACG-motif | | TGACG | | 306 | | 311 | | 5 | |  |
| TaDUF966-3B | TGA-element | | AACGAC | | 160 | | 166 | | 6 | |  |
| TaDUF966-3B | TGA-element | | AACGAC | | 1292 | | 1298 | | 6 | |  |
| TaDUF966-3B | TGA-element | | AACGAC | | 1391 | | 1397 | | 6 | |  |
| TaDUF966-3D | ABRE | | ACGTG | | 306 | | 311 | | 5 | |  |
| TaDUF966-3D | ABRE | | CACGTG | | 630 | | 636 | | 6 | |  |
| TaDUF966-3D | ABRE | | ACGTG | | 631 | | 636 | | 5 | |  |
| TaDUF966-3D | AE-box | | AGAAACAA | | 379 | | 387 | | 8 | |  |
| TaDUF966-3D | ARE | | AAACCA | | 218 | | 224 | | 6 | |  |
| TaDUF966-3D | ARE | | AAACCA | | 502 | | 508 | | 6 | |  |
| TaDUF966-3D | ARE | | AAACCA | | 1468 | | 1474 | | 6 | |  |
| TaDUF966-3D | CAAT-box | | CAAAT | | 77 | | 82 | | 5 | |  |
| TaDUF966-3D | CAAT-box | | CAAAT | | 84 | | 89 | | 5 | |  |
| TaDUF966-3D | CAAT-box | | CAAAT | | 114 | | 119 | | 5 | |  |
| TaDUF966-3D | CAAT-box | | CAAT | | 136 | | 140 | | 4 | |  |
| TaDUF966-3D | CAAT-box | | CCAAT | | 216 | | 221 | | 5 | |  |
| TaDUF966-3D | CAAT-box | | CAAT | | 227 | | 231 | | 4 | |  |
| TaDUF966-3D | CAAT-box | | CAAT | | 283 | | 287 | | 4 | |  |
| TaDUF966-3D | CAAT-box | | CAAT | | 289 | | 293 | | 4 | |  |
| TaDUF966-3D | CAAT-box | | CAAAT | | 316 | | 321 | | 5 | |  |
| TaDUF966-3D | CAAT-box | | CAAT | | 348 | | 352 | | 4 | |  |
| TaDUF966-3D | CAAT-box | | CAAAT | | 359 | | 364 | | 5 | |  |
| TaDUF966-3D | CAAT-box | | CAAT | | 439 | | 443 | | 4 | |  |
| TaDUF966-3D | CAAT-box | | CAAT | | 457 | | 461 | | 4 | |  |
| TaDUF966-3D | CAAT-box | | CAAT | | 473 | | 477 | | 4 | |  |
| TaDUF966-3D | CAAT-box | | CAAAT | | 601 | | 606 | | 5 | |  |
| TaDUF966-3D | CAAT-box | | CAAT | | 604 | | 608 | | 4 | |  |
| TaDUF966-3D | CAAT-box | | CAAT | | 637 | | 641 | | 4 | |  |
| TaDUF966-3D | CAAT-box | | TGCCAAC | | 1003 | | 1010 | | 7 | |  |
| TaDUF966-3D | CAAT-box | | CAAT | | 1143 | | 1147 | | 4 | |  |
| TaDUF966-3D | CAAT-box | | CAAT | | 1197 | | 1201 | | 4 | |  |
| TaDUF966-3D | CAAT-box | | CAAAT | | 1203 | | 1208 | | 5 | |  |
| TaDUF966-3D | CAAT-box | | CAAAT | | 1269 | | 1274 | | 5 | |  |
| TaDUF966-3D | CCAAT-box | | CAACGG | | 920 | | 926 | | 6 | |  |
| TaDUF966-3D | CGTCA-motif | | CGTCA | | 350 | | 355 | | 5 | |  |
| TaDUF966-3D | GATA-motif | | GATAGGA | | 939 | | 946 | | 7 | |  |
| TaDUF966-3D | GATA-motif | | AAGGATAAGG | | 1259 | | 1268 | | 9 | |  |
| TaDUF966-3D | G-Box | | CACGTG | | 630 | | 636 | | 6 | |  |
| TaDUF966-3D | G-box | | TACGTG | | 305 | | 311 | | 6 | |  |
| TaDUF966-3D | G-box | | CACGTG | | 630 | | 636 | | 6 | |  |
| TaDUF966-3D | G-box | | CACGAC | | 1117 | | 1123 | | 6 | |  |
| TaDUF966-3D | GC-motif | | CCCCCG | | 917 | | 923 | | 6 | |  |
| TaDUF966-3D | MBS | | CAACTG | | 677 | | 683 | | 6 | |  |
| TaDUF966-3D | MRE | | AACCTAA | | 1191 | | 1198 | | 7 | |  |
| TaDUF966-3D | TATA-box | | TATATTTATATTT | | 105 | | 117 | | 12 | |  |
| TaDUF966-3D | TATA-box | | TATAAATA | | 107 | | 115 | | 8 | |  |
| TaDUF966-3D | TATA-box | | TATAAAT | | 108 | | 115 | | 7 | |  |
| TaDUF966-3D | TATA-box | | TATAAA | | 109 | | 115 | | 6 | |  |
| TaDUF966-3D | TATA-box | | TATAA | | 110 | | 115 | | 5 | |  |
| TaDUF966-3D | TATA-box | | TATA | | 111 | | 115 | | 4 | |  |
| TaDUF966-3D | TATA-box | | ATATAT | | 639 | | 645 | | 6 | |  |
| TaDUF966-3D | TATA-box | | TATA | | 640 | | 644 | | 4 | |  |
| TaDUF966-3D | TATA-box | | TATATA | | 1066 | | 1072 | | 6 | |  |
| TaDUF966-3D | TATA-box | | ATATAT | | 1067 | | 1073 | | 6 | |  |
| TaDUF966-3D | TATA-box | | TATA | | 1068 | | 1072 | | 4 | |  |
| TaDUF966-3D | TATA-box | | ATATAT | | 1221 | | 1227 | | 6 | |  |
| TaDUF966-3D | TATA-box | | TATA | | 1222 | | 1226 | | 4 | |  |
| TaDUF966-3D | TATA-box | | ATATAA | | 1246 | | 1252 | | 6 | |  |
| TaDUF966-3D | TATA-box | | TATA | | 1247 | | 1251 | | 4 | |  |
| TaDUF966-3D | TATA-box | | ATATAA | | 1458 | | 1464 | | 6 | |  |
| TaDUF966-3D | TATA-box | | TATA | | 1459 | | 1463 | | 4 | |  |
| TaDUF966-3D | TC-rich repeats | | GTTTTCTTAC | | 1211 | | 1220 | | 9 | |  |
| TaDUF966-3D | TCT-motif | | TCTTAC | | 197 | | 203 | | 6 | |  |
| TaDUF966-3D | TGACG-motif | | TGACG | | 350 | | 355 | | 5 | |  |
| TaDUF966-3D | TGA-element | | AACGAC | | 1393 | | 1399 | | 6 | |  |
| TaDUF966-4A | ABRE | | GACACGTGGC | | 751 | | 760 | | 9 | |  |
| TaDUF966-4A | ABRE | | ACGTG | | 1319 | | 1324 | | 5 | |  |
| TaDUF966-4A | AE-box | | AGAAACAA | | 371 | | 379 | | 8 | |  |
| TaDUF966-4A | AE-box | | AGAAACAA | | 965 | | 973 | | 8 | |  |
| TaDUF966-4A | ARE | | AAACCA | | 687 | | 693 | | 6 | |  |
| TaDUF966-4A | CAAT-box | | CAAT | | 65 | | 69 | | 4 | |  |
| TaDUF966-4A | CAAT-box | | CCAAT | | 654 | | 659 | | 5 | |  |
| TaDUF966-4A | CAAT-box | | CAAT | | 655 | | 659 | | 4 | |  |
| TaDUF966-4A | CAAT-box | | CAAAT | | 843 | | 848 | | 5 | |  |
| TaDUF966-4A | CAAT-box | | CCAAT | | 860 | | 865 | | 5 | |  |
| TaDUF966-4A | CAAT-box | | CAAT | | 861 | | 865 | | 4 | |  |
| TaDUF966-4A | CAAT-box | | CAAAT | | 951 | | 956 | | 5 | |  |
| TaDUF966-4A | CAAT-box | | CAAT | | 987 | | 991 | | 4 | |  |
| TaDUF966-4A | CAAT-box | | CAAAT | | 1053 | | 1058 | | 5 | |  |
| TaDUF966-4A | CAAT-box | | CAAT | | 1060 | | 1064 | | 4 | |  |
| TaDUF966-4A | CAAT-box | | CAAT | | 1083 | | 1087 | | 4 | |  |
| TaDUF966-4A | CAAT-box | | CAAAT | | 1093 | | 1098 | | 5 | |  |
| TaDUF966-4A | CAAT-box | | CAAT | | 1280 | | 1284 | | 4 | |  |
| TaDUF966-4A | CAT-box | | GCCACT | | 755 | | 761 | | 6 | |  |
| TaDUF966-4A | CGTCA-motif | | CGTCA | | 773 | | 778 | | 5 | |  |
| TaDUF966-4A | G-box | | CACGAC | | 88 | | 94 | | 6 | |  |
| TaDUF966-4A | G-box | | CACGAC | | 783 | | 789 | | 6 | |  |
| TaDUF966-4A | G-box | | CACGTC | | 1318 | | 1324 | | 6 | |  |
| TaDUF966-4A | I-box | | gGATAAGGTG | | 1308 | | 1317 | | 9 | |  |
| TaDUF966-4A | LTR | | CCGAAA | | 96 | | 102 | | 6 | |  |
| TaDUF966-4A | Sp1 | | GGGCGG | | 893 | | 899 | | 6 | |  |
| TaDUF966-4A | TATA-box | | TACAAAA | | 1174 | | 1181 | | 7 | |  |
| TaDUF966-4A | TATA-box | | ATATAA | | 1255 | | 1261 | | 6 | |  |
| TaDUF966-4A | TATA-box | | TATA | | 1256 | | 1260 | | 4 | |  |
| TaDUF966-4A | TGACG-motif | | TGACG | | 773 | | 778 | | 5 | |  |
| TaDUF966-4B | A-box | | CCGTCC | | 330 | | 336 | | 6 | |  |
| TaDUF966-4B | ABRE | | ACGTG | | 143 | | 148 | | 5 | |  |
| TaDUF966-4B | ARE | | AAACCA | | 289 | | 295 | | 6 | |  |
| TaDUF966-4B | Box 4 | | ATTAAT | | 17 | | 23 | | 6 | |  |
| TaDUF966-4B | CAAT-box | | CAAAT | | 4 | | 9 | | 5 | |  |
| TaDUF966-4B | CAAT-box | | CAAAT | | 286 | | 291 | | 5 | |  |
| TaDUF966-4B | CAAT-box | | CAAAT | | 389 | | 394 | | 5 | |  |
| TaDUF966-4B | CAAT-box | | CAAT | | 470 | | 474 | | 4 | |  |
| TaDUF966-4B | CAAT-box | | CAAT | | 612 | | 616 | | 4 | |  |
| TaDUF966-4B | CAAT-box | | CAAAT | | 649 | | 654 | | 5 | |  |
| TaDUF966-4B | CAAT-box | | CAAAT | | 657 | | 662 | | 5 | |  |
| TaDUF966-4B | CAAT-box | | CAAAT | | 689 | | 694 | | 5 | |  |
| TaDUF966-4B | CAAT-box | | CAAT | | 696 | | 700 | | 4 | |  |
| TaDUF966-4B | CAAT-box | | CAAT | | 738 | | 742 | | 4 | |  |
| TaDUF966-4B | CAAT-box | | CAAT | | 855 | | 859 | | 4 | |  |
| TaDUF966-4B | CAAT-box | | CAAT | | 916 | | 920 | | 4 | |  |
| TaDUF966-4B | CAAT-box | | CAAAT | | 1050 | | 1055 | | 5 | |  |
| TaDUF966-4B | CAAT-box | | CCAAT | | 1111 | | 1116 | | 5 | |  |
| TaDUF966-4B | CAAT-box | | CAAT | | 1112 | | 1116 | | 4 | |  |
| TaDUF966-4B | CAAT-box | | CAAT | | 1114 | | 1118 | | 4 | |  |
| TaDUF966-4B | CAAT-box | | CAAT | | 1173 | | 1177 | | 4 | |  |
| TaDUF966-4B | CAAT-box | | CAAT | | 1180 | | 1184 | | 4 | |  |
| TaDUF966-4B | CAAT-box | | CCAAT | | 1274 | | 1279 | | 5 | |  |
| TaDUF966-4B | CAAT-box | | CAAT | | 1275 | | 1279 | | 4 | |  |
| TaDUF966-4B | CGTCA-motif | | CGTCA | | 678 | | 683 | | 5 | |  |
| TaDUF966-4B | GATA-motif | | AAGGATAAGG | | 1323 | | 1332 | | 9 | |  |
| TaDUF966-4B | G-box | | CACGTC | | 143 | | 149 | | 6 | |  |
| TaDUF966-4B | GT1-motif | | GGTTAAT | | 768 | | 775 | | 7 | |  |
| TaDUF966-4B | I-box | | gGATAAGGTG | | 1321 | | 1330 | | 9 | |  |
| TaDUF966-4B | I-box | | ccttatcct | | 1323 | | 1332 | | 9 | |  |
| TaDUF966-4B | O2-site | | GATGACATGG | | 1012 | | 1021 | | 9 | |  |
| TaDUF966-4B | O2-site | | GATGATGTGG | | 1219 | | 1228 | | 9 | |  |
| TaDUF966-4B | P-box | | CCTTTTG | | 1351 | | 1358 | | 7 | |  |
| TaDUF966-4B | Sp1 | | GGGCGG | | 1094 | | 1100 | | 6 | |  |
| TaDUF966-4B | Sp1 | | GGGCGG | | 1266 | | 1272 | | 6 | |  |
| TaDUF966-4B | Sp1 | | GGGCGG | | 1270 | | 1276 | | 6 | |  |
| TaDUF966-4B | TATA-box | | TATA | | 621 | | 625 | | 4 | |  |
| TaDUF966-4B | TCA-element | | TCAGAAGAGG | | 484 | | 493 | | 9 | |  |
| TaDUF966-4B | TCA-element | | CCATCTTTTT | | 817 | | 826 | | 9 | |  |
| TaDUF966-4B | TGACG-motif | | TGACG | | 678 | | 683 | | 5 | |  |
| TaDUF966-4D | ABRE | | CACGTG | | 102 | | 108 | | 6 | |  |
| TaDUF966-4D | ABRE | | ACGTG | | 103 | | 108 | | 5 | |  |
| TaDUF966-4D | ABRE | | TACGGTC | | 706 | | 713 | | 7 | |  |
| TaDUF966-4D | AE-box | | AGAAACAA | | 158 | | 166 | | 8 | |  |
| TaDUF966-4D | ARE | | AAACCA | | 440 | | 446 | | 6 | |  |
| TaDUF966-4D | Box 4 | | ATTAAT | | 1234 | | 1240 | | 6 | |  |
| TaDUF966-4D | CAAT-box | | CAAAT | | 5 | | 10 | | 5 | |  |
| TaDUF966-4D | CAAT-box | | CAAT | | 79 | | 83 | | 4 | |  |
| TaDUF966-4D | CAAT-box | | CAAT | | 180 | | 184 | | 4 | |  |
| TaDUF966-4D | CAAT-box | | CAAT | | 187 | | 191 | | 4 | |  |
| TaDUF966-4D | CAAT-box | | CAAT | | 233 | | 237 | | 4 | |  |
| TaDUF966-4D | CAAT-box | | CAAAT | | 271 | | 276 | | 5 | |  |
| TaDUF966-4D | CAAT-box | | CAAAT | | 274 | | 279 | | 5 | |  |
| TaDUF966-4D | CAAT-box | | CAAAT | | 283 | | 288 | | 5 | |  |
| TaDUF966-4D | CAAT-box | | CAAT | | 289 | | 293 | | 4 | |  |
| TaDUF966-4D | CAAT-box | | CAAAT | | 306 | | 311 | | 5 | |  |
| TaDUF966-4D | CAAT-box | | CAAT | | 575 | | 579 | | 4 | |  |
| TaDUF966-4D | CAAT-box | | CAAT | | 762 | | 766 | | 4 | |  |
| TaDUF966-4D | CAAT-box | | CAAT | | 967 | | 971 | | 4 | |  |
| TaDUF966-4D | CAAT-box | | CAAT | | 1125 | | 1129 | | 4 | |  |
| TaDUF966-4D | CAAT-box | | CAAT | | 1127 | | 1131 | | 4 | |  |
| TaDUF966-4D | CAAT-box | | CAAAT | | 1281 | | 1286 | | 5 | |  |
| TaDUF966-4D | CAAT-box | | CAAAT | | 1296 | | 1301 | | 5 | |  |
| TaDUF966-4D | CAAT-box | | CAAT | | 1312 | | 1316 | | 4 | |  |
| TaDUF966-4D | CCAAT-box | | CAACGG | | 805 | | 811 | | 6 | |  |
| TaDUF966-4D | CGTCA-motif | | CGTCA | | 877 | | 882 | | 5 | |  |
| TaDUF966-4D | GATA-motif | | GATAGGA | | 713 | | 720 | | 7 | |  |
| TaDUF966-4D | G-Box | | CACGTG | | 102 | | 108 | | 6 | |  |
| TaDUF966-4D | G-box | | CACGTG | | 102 | | 108 | | 6 | |  |
| TaDUF966-4D | GT1-motif | | GGTTAA | | 1249 | | 1255 | | 6 | |  |
| TaDUF966-4D | LTR | | CCGAAA | | 475 | | 481 | | 6 | |  |
| TaDUF966-4D | LTR | | CCGAAA | | 841 | | 847 | | 6 | |  |
| TaDUF966-4D | MBS | | CAACTG | | 598 | | 604 | | 6 | |  |
| TaDUF966-4D | MRE | | AACCTAA | | 485 | | 492 | | 7 | |  |
| TaDUF966-4D | P-box | | CCTTTTG | | 825 | | 832 | | 7 | |  |
| TaDUF966-4D | P-box | | CCTTTTG | | 1033 | | 1040 | | 7 | |  |
| TaDUF966-4D | TATA-box | | TATTTAAA | | 206 | | 214 | | 8 | |  |
| TaDUF966-4D | TATA-box | | ATTATA | | 240 | | 246 | | 6 | |  |
| TaDUF966-4D | TATA-box | | TATAA | | 241 | | 246 | | 5 | |  |
| TaDUF966-4D | TATA-box | | TATA | | 242 | | 246 | | 4 | |  |
| TaDUF966-4D | TATA-box | | TACAAAA | | 259 | | 266 | | 7 | |  |
| TaDUF966-4D | TATA-box | | TATAA | | 294 | | 299 | | 5 | |  |
| TaDUF966-4D | TATA-box | | TATA | | 295 | | 299 | | 4 | |  |
| TaDUF966-4D | TATA-box | | TACAAAA | | 378 | | 385 | | 7 | |  |
| TaDUF966-4D | TCA-element | | CCATCTTTTT | | 227 | | 236 | | 9 | |  |
| TaDUF966-4D | TGACG-motif | | TGACG | | 877 | | 882 | | 5 | |  |
| TaDUF966-4D | TGA-element | | AACGAC | | 542 | | 548 | | 6 | |  |
| TaDUF966-5A | ABRE | | ACGTG | | 255 | | 260 | | 5 | |  |
| TaDUF966-5A | ABRE | | ACGTG | | 298 | | 303 | | 5 | |  |
| TaDUF966-5A | ABRE | | CACGTG | | 545 | | 551 | | 6 | |  |
| TaDUF966-5A | ABRE | | ACGTG | | 546 | | 551 | | 5 | |  |
| TaDUF966-5A | ABRE | | GCCGCGTGGC | | 670 | | 679 | | 9 | |  |
| TaDUF966-5A | ABRE | | ACGTG | | 1123 | | 1128 | | 5 | |  |
| TaDUF966-5A | ABRE | | ACGTG | | 1143 | | 1148 | | 5 | |  |
| TaDUF966-5A | AE-box | | AGAAACAA | | 556 | | 564 | | 8 | |  |
| TaDUF966-5A | CAAT-box | | CAAAT | | 83 | | 88 | | 5 | |  |
| TaDUF966-5A | CAAT-box | | CAAT | | 448 | | 452 | | 4 | |  |
| TaDUF966-5A | CAAT-box | | CAAT | | 717 | | 721 | | 4 | |  |
| TaDUF966-5A | CAAT-box | | CAAAT | | 862 | | 867 | | 5 | |  |
| TaDUF966-5A | CAAT-box | | CAAT | | 974 | | 978 | | 4 | |  |
| TaDUF966-5A | CAAT-box | | CAAT | | 1214 | | 1218 | | 4 | |  |
| TaDUF966-5A | CAAT-box | | CAAT | | 1313 | | 1317 | | 4 | |  |
| TaDUF966-5A | CAAT-box | | CAAAT | | 1317 | | 1322 | | 5 | |  |
| TaDUF966-5A | CAAT-box | | CAAT | | 1341 | | 1345 | | 4 | |  |
| TaDUF966-5A | CAAT-box | | CCAAT | | 1492 | | 1497 | | 5 | |  |
| TaDUF966-5A | CAT-box | | GCCACT | | 239 | | 245 | | 6 | |  |
| TaDUF966-5A | CCAAT-box | | CAACGG | | 60 | | 66 | | 6 | |  |
| TaDUF966-5A | CCAAT-box | | CAACGG | | 830 | | 836 | | 6 | |  |
| TaDUF966-5A | CGTCA-motif | | CGTCA | | 252 | | 257 | | 5 | |  |
| TaDUF966-5A | CGTCA-motif | | CGTCA | | 257 | | 262 | | 5 | |  |
| TaDUF966-5A | CGTCA-motif | | CGTCA | | 603 | | 608 | | 5 | |  |
| TaDUF966-5A | CGTCA-motif | | CGTCA | | 617 | | 622 | | 5 | |  |
| TaDUF966-5A | CGTCA-motif | | CGTCA | | 1125 | | 1130 | | 5 | |  |
| TaDUF966-5A | CGTCA-motif | | CGTCA | | 1163 | | 1168 | | 5 | |  |
| TaDUF966-5A | G-Box | | CACGTT | | 298 | | 304 | | 6 | |  |
| TaDUF966-5A | G-Box | | CACGTG | | 545 | | 551 | | 6 | |  |
| TaDUF966-5A | G-Box | | CACGTT | | 1143 | | 1149 | | 6 | |  |
| TaDUF966-5A | G-box | | CACGTC | | 255 | | 261 | | 6 | |  |
| TaDUF966-5A | G-box | | CACGTG | | 545 | | 551 | | 6 | |  |
| TaDUF966-5A | G-box | | CACGTC | | 1123 | | 1129 | | 6 | |  |
| TaDUF966-5A | GC-motif | | CCCCCG | | 398 | | 404 | | 6 | |  |
| TaDUF966-5A | GT1-motif | | GGTTAA | | 551 | | 557 | | 6 | |  |
| TaDUF966-5A | I-box | | GTATAAGGCC | | 520 | | 529 | | 9 | |  |
| TaDUF966-5A | MBS | | CAACTG | | 97 | | 103 | | 6 | |  |
| TaDUF966-5A | MBS | | CAACTG | | 1131 | | 1137 | | 6 | |  |
| TaDUF966-5A | MRE | | AACCTAA | | 459 | | 466 | | 7 | |  |
| TaDUF966-5A | O2-site | | GATGATGTGG | | 39 | | 48 | | 9 | |  |
| TaDUF966-5A | O2-site | | GTTGACGTGA | | 1122 | | 1132 | | 10 | |  |
| TaDUF966-5A | Sp1 | | GGGCGG | | 18 | | 24 | | 6 | |  |
| TaDUF966-5A | Sp1 | | GGGCGG | | 528 | | 534 | | 6 | |  |
| TaDUF966-5A | Sp1 | | GGGCGG | | 621 | | 627 | | 6 | |  |
| TaDUF966-5A | TATA-box | | TATA | | 897 | | 901 | | 4 | |  |
| TaDUF966-5A | TATA-box | | TATA | | 950 | | 954 | | 4 | |  |
| TaDUF966-5A | TATA-box | | TATAA | | 1147 | | 1152 | | 5 | |  |
| TaDUF966-5A | TATA-box | | TATA | | 1148 | | 1152 | | 4 | |  |
| TaDUF966-5A | TATA-box | | TACATAAA | | 1277 | | 1285 | | 8 | |  |
| TaDUF966-5A | TATA-box | | TATAA | | 1354 | | 1359 | | 5 | |  |
| TaDUF966-5A | TATA-box | | TATA | | 1355 | | 1359 | | 4 | |  |
| TaDUF966-5A | TATA-box | | TATAAAA | | 1413 | | 1420 | | 7 | |  |
| TaDUF966-5A | TATA-box | | TATAAA | | 1414 | | 1420 | | 6 | |  |
| TaDUF966-5A | TATA-box | | TATAA | | 1415 | | 1420 | | 5 | |  |
| TaDUF966-5A | TATA-box | | TATA | | 1416 | | 1420 | | 4 | |  |
| TaDUF966-5A | TC-rich repeats | | ATTCTCTAAC | | 1328 | | 1337 | | 9 | |  |
| TaDUF966-5A | TGACG-motif | | TGACG | | 252 | | 257 | | 5 | |  |
| TaDUF966-5A | TGACG-motif | | TGACG | | 257 | | 262 | | 5 | |  |
| TaDUF966-5A | TGACG-motif | | TGACG | | 603 | | 608 | | 5 | |  |
| TaDUF966-5A | TGACG-motif | | TGACG | | 617 | | 622 | | 5 | |  |
| TaDUF966-5A | TGACG-motif | | TGACG | | 1125 | | 1130 | | 5 | |  |
| TaDUF966-5A | TGACG-motif | | TGACG | | 1163 | | 1168 | | 5 | |  |
| TaDUF966-5A | TGA-element | | AACGAC | | 271 | | 277 | | 6 | |  |
| TaDUF966-5B | A-box | | CCGTCC | | 28 | | 34 | | 6 | |  |
| TaDUF966-5B | A-box | | CCGTCC | | 626 | | 632 | | 6 | |  |
| TaDUF966-5B | A-box | | CCGTCC | | 790 | | 796 | | 6 | |  |
| TaDUF966-5B | ABRE | | ACGTG | | 661 | | 666 | | 5 | |  |
| TaDUF966-5B | ABRE | | ACGTG | | 770 | | 775 | | 5 | |  |
| TaDUF966-5B | ABRE | | GCCGCGTGGC | | 1259 | | 1268 | | 9 | |  |
| TaDUF966-5B | Box 4 | | ATTAAT | | 42 | | 48 | | 6 | |  |
| TaDUF966-5B | CAAT-box | | CAAT | | 82 | | 86 | | 4 | |  |
| TaDUF966-5B | CAAT-box | | CAAAT | | 104 | | 109 | | 5 | |  |
| TaDUF966-5B | CAAT-box | | CAAAT | | 204 | | 209 | | 5 | |  |
| TaDUF966-5B | CAAT-box | | CAAT | | 513 | | 517 | | 4 | |  |
| TaDUF966-5B | CAAT-box | | CAAT | | 520 | | 524 | | 4 | |  |
| TaDUF966-5B | CAAT-box | | CAAAT | | 971 | | 976 | | 5 | |  |
| TaDUF966-5B | CAAT-box | | CCAAT | | 1070 | | 1075 | | 5 | |  |
| TaDUF966-5B | CAAT-box | | CAAT | | 1105 | | 1109 | | 4 | |  |
| TaDUF966-5B | CAAT-box | | CAAAT | | 1227 | | 1232 | | 5 | |  |
| TaDUF966-5B | CAAT-box | | CAAT | | 1336 | | 1340 | | 4 | |  |
| TaDUF966-5B | CAT-box | | GCCACT | | 1297 | | 1303 | | 6 | |  |
| TaDUF966-5B | CCAAT-box | | CAACGG | | 215 | | 221 | | 6 | |  |
| TaDUF966-5B | CGTCA-motif | | CGTCA | | 16 | | 21 | | 5 | |  |
| TaDUF966-5B | CGTCA-motif | | CGTCA | | 768 | | 773 | | 5 | |  |
| TaDUF966-5B | GATA-motif | | GATAGGG | | 933 | | 940 | | 7 | |  |
| TaDUF966-5B | G-box | | CACGAC | | 22 | | 28 | | 6 | |  |
| TaDUF966-5B | G-box | | CACGAC | | 77 | | 83 | | 6 | |  |
| TaDUF966-5B | G-box | | CACGTC | | 660 | | 666 | | 6 | |  |
| TaDUF966-5B | G-box | | CACGTC | | 769 | | 775 | | 6 | |  |
| TaDUF966-5B | O2-site | | GATGA(C/T)(A/G)TG(A/G) | | 761 | | 769.5 | | 8.5 | |  |
| TaDUF966-5B | TATA-box | | ATATAT | | 1029 | | 1035 | | 6 | |  |
| TaDUF966-5B | TATA-box | | TATA | | 1030 | | 1034 | | 4 | |  |
| TaDUF966-5B | TATA-box | | TATAAAA | | 1083 | | 1090 | | 7 | |  |
| TaDUF966-5B | TATA-box | | TATAAA | | 1084 | | 1090 | | 6 | |  |
| TaDUF966-5B | TATA-box | | TATAA | | 1085 | | 1090 | | 5 | |  |
| TaDUF966-5B | TATA-box | | TATA | | 1086 | | 1090 | | 4 | |  |
| TaDUF966-5B | TATA-box | | TATAA | | 1273 | | 1278 | | 5 | |  |
| TaDUF966-5B | TATA-box | | TATA | | 1274 | | 1278 | | 4 | |  |
| TaDUF966-5B | TGACG-motif | | TGACG | | 16 | | 21 | | 5 | |  |
| TaDUF966-5B | TGACG-motif | | TGACG | | 768 | | 773 | | 5 | |  |
| TaDUF966-5B | TGA-element | | AACGAC | | 1166 | | 1172 | | 6 | |  |
| TaDUF966-5D | ABRE | | GCCGCGTGGC | | 1252 | | 1261 | | 9 | |  |
| TaDUF966-5D | ABRE | | ACGTG | | 1440 | | 1445 | | 5 | |  |
| TaDUF966-5D | CAAT-box | | CCAAT | | 65 | | 70 | | 5 | |  |
| TaDUF966-5D | CAAT-box | | CCAAT | | 74 | | 79 | | 5 | |  |
| TaDUF966-5D | CAAT-box | | CAAT | | 168 | | 172 | | 4 | |  |
| TaDUF966-5D | CAAT-box | | CAAT | | 176 | | 180 | | 4 | |  |
| TaDUF966-5D | CAAT-box | | CAAAT | | 187 | | 192 | | 5 | |  |
| TaDUF966-5D | CAAT-box | | CAAT | | 199 | | 203 | | 4 | |  |
| TaDUF966-5D | CAAT-box | | CCAAT | | 786 | | 791 | | 5 | |  |
| TaDUF966-5D | CAAT-box | | CAAT | | 792 | | 796 | | 4 | |  |
| TaDUF966-5D | CAAT-box | | CAAAT | | 796 | | 801 | | 5 | |  |
| TaDUF966-5D | CAAT-box | | CCAAT | | 841 | | 846 | | 5 | |  |
| TaDUF966-5D | CAAT-box | | CAAT | | 842 | | 846 | | 4 | |  |
| TaDUF966-5D | CAAT-box | | CAAAT | | 1017 | | 1022 | | 5 | |  |
| TaDUF966-5D | CAAT-box | | CCAAT | | 1116 | | 1121 | | 5 | |  |
| TaDUF966-5D | CAAT-box | | CAAAT | | 1220 | | 1225 | | 5 | |  |
| TaDUF966-5D | CAT-box | | GCCACT | | 1292 | | 1298 | | 6 | |  |
| TaDUF966-5D | CCAAT-box | | CAACGG | | 825 | | 831 | | 6 | |  |
| TaDUF966-5D | G-box | | CACGTC | | 1440 | | 1446 | | 6 | |  |
| TaDUF966-5D | MBS | | CAACTG | | 28 | | 34 | | 6 | |  |
| TaDUF966-5D | TATA-box | | TATA | | 125 | | 129 | | 4 | |  |
| TaDUF966-5D | TATA-box | | ATATAT | | 1075 | | 1081 | | 6 | |  |
| TaDUF966-5D | TATA-box | | TATA | | 1076 | | 1080 | | 4 | |  |
| TaDUF966-5D | TATA-box | | TATAAAA | | 1129 | | 1136 | | 7 | |  |
| TaDUF966-5D | TATA-box | | TATAAA | | 1130 | | 1136 | | 6 | |  |
| TaDUF966-5D | TATA-box | | TATAA | | 1131 | | 1136 | | 5 | |  |
| TaDUF966-5D | TATA-box | | TATA | | 1132 | | 1136 | | 4 | |  |
| TaDUF966-5D | TATA-box | | ATATAA | | 1368 | | 1374 | | 6 | |  |
| TaDUF966-5D | TATA-box | | TATA | | 1369 | | 1373 | | 4 | |  |
| TaDUF966-6B | ABRE | | CACGTG | | 1028 | | 1034 | | 6 | |  |
| TaDUF966-6B | ABRE | | ACGTG | | 1029 | | 1034 | | 5 | |  |
| TaDUF966-6B | AE-box | | AGAAACAA | | 101 | | 109 | | 8 | |  |
| TaDUF966-6B | Box 4 | | ATTAAT | | 1092 | | 1098 | | 6 | |  |
| TaDUF966-6B | CAAT-box | | CAAT | | 90 | | 94 | | 4 | |  |
| TaDUF966-6B | CAAT-box | | CAAAT | | 160 | | 165 | | 5 | |  |
| TaDUF966-6B | CAAT-box | | CAAAT | | 210 | | 215 | | 5 | |  |
| TaDUF966-6B | CAAT-box | | CAAAT | | 253 | | 258 | | 5 | |  |
| TaDUF966-6B | CAAT-box | | CAAT | | 319 | | 323 | | 4 | |  |
| TaDUF966-6B | CAAT-box | | CAAAT | | 353 | | 358 | | 5 | |  |
| TaDUF966-6B | CAAT-box | | CAAAT | | 444 | | 449 | | 5 | |  |
| TaDUF966-6B | CAAT-box | | CAAT | | 454 | | 458 | | 4 | |  |
| TaDUF966-6B | CAAT-box | | CAAT | | 469 | | 473 | | 4 | |  |
| TaDUF966-6B | CAAT-box | | CAAT | | 589 | | 593 | | 4 | |  |
| TaDUF966-6B | CAAT-box | | CAAAT | | 597 | | 602 | | 5 | |  |
| TaDUF966-6B | CAAT-box | | CAAT | | 624 | | 628 | | 4 | |  |
| TaDUF966-6B | CAAT-box | | CAAAT | | 634 | | 639 | | 5 | |  |
| TaDUF966-6B | CAAT-box | | CAAT | | 672 | | 676 | | 4 | |  |
| TaDUF966-6B | CAAT-box | | CAAT | | 684 | | 688 | | 4 | |  |
| TaDUF966-6B | CAAT-box | | CAAT | | 718 | | 722 | | 4 | |  |
| TaDUF966-6B | CAAT-box | | CAAT | | 753 | | 757 | | 4 | |  |
| TaDUF966-6B | CAAT-box | | CAAT | | 961 | | 965 | | 4 | |  |
| TaDUF966-6B | CAAT-box | | CAAT | | 989 | | 993 | | 4 | |  |
| TaDUF966-6B | CAAT-box | | CAAAT | | 1068 | | 1073 | | 5 | |  |
| TaDUF966-6B | CAAT-box | | CAAT | | 1081 | | 1085 | | 4 | |  |
| TaDUF966-6B | CAAT-box | | TGCCAAC | | 1152 | | 1159 | | 7 | |  |
| TaDUF966-6B | CAAT-box | | CAAAT | | 1191 | | 1196 | | 5 | |  |
| TaDUF966-6B | CAAT-box | | CAAT | | 1217 | | 1221 | | 4 | |  |
| TaDUF966-6B | CAAT-box | | CAAT | | 1254 | | 1258 | | 4 | |  |
| TaDUF966-6B | CAAT-box | | CAAAT | | 1283 | | 1288 | | 5 | |  |
| TaDUF966-6B | CAAT-box | | CAAT | | 1292 | | 1296 | | 4 | |  |
| TaDUF966-6B | CAAT-box | | CAAT | | 1324 | | 1328 | | 4 | |  |
| TaDUF966-6B | CAAT-box | | CCAAT | | 1398 | | 1403 | | 5 | |  |
| TaDUF966-6B | CAT-box | | GCCACT | | 809 | | 815 | | 6 | |  |
| TaDUF966-6B | CCAAT-box | | CAACGG | | 106 | | 112 | | 6 | |  |
| TaDUF966-6B | CGTCA-motif | | CGTCA | | 507 | | 512 | | 5 | |  |
| TaDUF966-6B | G-Box | | CACGTG | | 1028 | | 1034 | | 6 | |  |
| TaDUF966-6B | G-box | | CACGTG | | 1028 | | 1034 | | 6 | |  |
| TaDUF966-6B | GT1-motif | | GGTTAA | | 184 | | 190 | | 6 | |  |
| TaDUF966-6B | I-box | | TGATAATGT | | 1420 | | 1429 | | 9 | |  |
| TaDUF966-6B | TATA-box | | taTATAAAtc | | 400 | | 409 | | 9 | |  |
| TaDUF966-6B | TATA-box | | ATATAA | | 401 | | 407 | | 6 | |  |
| TaDUF966-6B | TATA-box | | TATA | | 402 | | 406 | | 4 | |  |
| TaDUF966-6B | TATA-box | | TATA | | 413 | | 417 | | 4 | |  |
| TaDUF966-6B | TATA-box | | TATAAAT | | 485 | | 492 | | 7 | |  |
| TaDUF966-6B | TATA-box | | TATAAA | | 486 | | 492 | | 6 | |  |
| TaDUF966-6B | TATA-box | | TATAA | | 487 | | 492 | | 5 | |  |
| TaDUF966-6B | TATA-box | | TATA | | 488 | | 492 | | 4 | |  |
| TaDUF966-6B | TATA-box | | ATATAT | | 613 | | 619 | | 6 | |  |
| TaDUF966-6B | TATA-box | | TATATA | | 614 | | 620 | | 6 | |  |
| TaDUF966-6B | TATA-box | | TATA | | 616 | | 620 | | 4 | |  |
| TaDUF966-6B | TATA-box | | TATATAA | | 836 | | 843 | | 7 | |  |
| TaDUF966-6B | TATA-box | | TATATA | | 837 | | 843 | | 6 | |  |
| TaDUF966-6B | TATA-box | | TATA | | 839 | | 843 | | 4 | |  |
| TaDUF966-6B | TATA-box | | ATTATA | | 991 | | 997 | | 6 | |  |
| TaDUF966-6B | TATA-box | | TATAA | | 992 | | 997 | | 5 | |  |
| TaDUF966-6B | TATA-box | | TATA | | 993 | | 997 | | 4 | |  |
| TaDUF966-6B | TATA-box | | TATAA | | 1018 | | 1023 | | 5 | |  |
| TaDUF966-6B | TATA-box | | TATA | | 1019 | | 1023 | | 4 | |  |
| TaDUF966-6B | TATA-box | | TATA | | 1051 | | 1055 | | 4 | |  |
| TaDUF966-6B | TATA-box | | TATACA | | 1097 | | 1103 | | 6 | |  |
| TaDUF966-6B | TATA-box | | TATA | | 1099 | | 1103 | | 4 | |  |
| TaDUF966-6B | TATA-box | | TATACA | | 1133 | | 1139 | | 6 | |  |
| TaDUF966-6B | TATA-box | | TATATA | | 1135 | | 1141 | | 6 | |  |
| TaDUF966-6B | TATA-box | | ATATAT | | 1136 | | 1142 | | 6 | |  |
| TaDUF966-6B | TATA-box | | TATA | | 1137 | | 1141 | | 4 | |  |
| TaDUF966-6B | TATA-box | | TATATTTATATTT | | 1197 | | 1210 | | 13 | |  |
| TaDUF966-6B | TATA-box | | TATAAATA | | 1199 | | 1207 | | 8 | |  |
| TaDUF966-6B | TATA-box | | TATAAAT | | 1200 | | 1207 | | 7 | |  |
| TaDUF966-6B | TATA-box | | TATAAA | | 1201 | | 1207 | | 6 | |  |
| TaDUF966-6B | TATA-box | | TATAA | | 1202 | | 1207 | | 5 | |  |
| TaDUF966-6B | TATA-box | | TATA | | 1203 | | 1207 | | 4 | |  |
| TaDUF966-6B | TATA-box | | TATA | | 1275 | | 1279 | | 4 | |  |
| TaDUF966-6B | TATA-box | | taTATAAAtc | | 1309 | | 1318 | | 9 | |  |
| TaDUF966-6B | TATA-box | | ATATAT | | 1310 | | 1316 | | 6 | |  |
| TaDUF966-6B | TATA-box | | TATATA | | 1311 | | 1317 | | 6 | |  |
| TaDUF966-6B | TATA-box | | ATATAT | | 1312 | | 1318 | | 6 | |  |
| TaDUF966-6B | TATA-box | | TATATA | | 1313 | | 1319 | | 6 | |  |
| TaDUF966-6B | TATA-box | | TATA | | 1315 | | 1319 | | 4 | |  |
| TaDUF966-6B | TATA-box | | TATA | | 1365 | | 1369 | | 4 | |  |
| TaDUF966-6B | TATA-box | | TATAA | | 1474 | | 1479 | | 5 | |  |
| TaDUF966-6B | TATA-box | | TATA | | 1475 | | 1479 | | 4 | |  |
| TaDUF966-6B | TC-rich repeats | | ATTCTCTAAC | | 265 | | 274 | | 9 | |  |
| TaDUF966-6B | TC-rich repeats | | GTTTTCTTAC | | 1072 | | 1081 | | 9 | |  |
| TaDUF966-6B | TCT-motif | | TCTTAC | | 537 | | 543 | | 6 | |  |
| TaDUF966-6B | TGACG-motif | | TGACG | | 507 | | 512 | | 5 | |  |
| TaDUF966-7D | GC-motif | | CCCCCG | | 868 | | 874 | | 6 | |  |
| TaDUF966-7D | GC-motif | | CCCCCG | | 933 | | 939 | | 6 | |  |
| TaDUF966-7D | GC-motif | | CCCCCG | | 1069 | | 1075 | | 6 | |  |
| TaDUF966-7D | GC-motif | | CCCCCG | | 1075 | | 1081 | | 6 | |  |
| TaDUF966-7D | GC-motif | | CCCCCG | | 1108 | | 1114 | | 6 | |  |
| TaDUF966-7D | GC-motif | | CCCCCG | | 1139 | | 1145 | | 6 | |  |
| TaDUF966-7D | O2-site | | GATGA(C/T)(A/G)TG(A/G) | | 509 | | 517.5 | | 8.5 | |  |
| TaDUF966-7D | CAT-box | | GCCACT | | 841 | | 847 | | 6 | |  |
| TaDUF966-7D | TATA-box | | TATA | | 143 | | 147 | | 4 | |  |
| TaDUF966-7D | TATA-box | | TATA | | 576 | | 580 | | 4 | |  |
| TaDUF966-7D | TATA-box | | taTATAAAtc | | 619 | | 628 | | 9 | |  |
| TaDUF966-7D | TATA-box | | TATAAA | | 621 | | 627 | | 6 | |  |
| TaDUF966-7D | TATA-box | | TATATAA | | 622 | | 629 | | 7 | |  |
| TaDUF966-7D | TATA-box | | TATATA | | 623 | | 629 | | 6 | |  |
| TaDUF966-7D | TATA-box | | TATA | | 625 | | 629 | | 4 | |  |
| TaDUF966-7D | TATA-box | | TATATA | | 788 | | 794 | | 6 | |  |
| TaDUF966-7D | TATA-box | | TATA | | 790 | | 794 | | 4 | |  |
| TaDUF966-7D | TATA-box | | TAAAGATT | | 1371 | | 1379 | | 8 | |  |
| TaDUF966-7D | CAAT-box | | CAAT | | 321 | | 325 | | 4 | |  |
| TaDUF966-7D | CAAT-box | | CAAT | | 345 | | 349 | | 4 | |  |
| TaDUF966-7D | CAAT-box | | CCAAT | | 356 | | 361 | | 5 | |  |
| TaDUF966-7D | CAAT-box | | CAAT | | 357 | | 361 | | 4 | |  |
| TaDUF966-7D | CAAT-box | | CAAT | | 443 | | 447 | | 4 | |  |
| TaDUF966-7D | CAAT-box | | CAAT | | 500 | | 504 | | 4 | |  |
| TaDUF966-7D | CAAT-box | | CAAT | | 586 | | 590 | | 4 | |  |
| TaDUF966-7D | CAAT-box | | CAAT | | 691 | | 695 | | 4 | |  |
| TaDUF966-7D | CAAT-box | | CAAT | | 750 | | 754 | | 4 | |  |
| TaDUF966-7D | CAAT-box | | CAAT | | 1357 | | 1361 | | 4 | |  |
| TaDUF966-7D | CAAT-box | | CAAAT | | 1369 | | 1374 | | 5 | |  |
| TaDUF966-7D | CAAT-box | | CAAAT | | 1388 | | 1393 | | 5 | |  |
| TaDUF966-7D | G-box | | CACGAC | | 72 | | 78 | | 6 | |  |
| TaDUF966-7D | G-box | | CACGAC | | 985 | | 991 | | 6 | |  |
| TaDUF966-7D | CGTCA-motif | | CGTCA | | 117 | | 122 | | 5 | |  |
| TaDUF966-7D | Sp1 | | GGGCGG | | 1029 | | 1035 | | 6 | |  |
| TaDUF966-7D | Sp1 | | GGGCGG | | 1072 | | 1078 | | 6 | |  |
| TaDUF966-7D | A-box | | CCGTCC | | 898 | | 904 | | 6 | |  |
| TaDUF966-7D | CCAAT-box | | CAACGG | | 951 | | 957 | | 6 | |  |
| TaDUF966-7D | TGACG-motif | | TGACG | | 117 | | 122 | | 5 | |  |
| TaDUF966-7D | GT1-motif | | GGTTAA | | 1153 | | 1159 | | 6 | |  |
| TaDUF966-7D | ARE | | AAACCA | | 618 | | 624 | | 6 | |  |
| TaDUF966-7D | ARE | | AAACCA | | 1404 | | 1410 | | 6 | |  |
| TaDUF966-7D | ARE | | AAACCA | | 1426 | | 1432 | | 6 | |  |
| TaDUF966-7D | MBS | | CAACTG | | 1220 | | 1226 | | 6 | |  |
| TaDUF966-8A | CAAT-box | | CAAT | | 5 | | 9 | | 4 | |  |
| TaDUF966-8A | CAAT-box | | CAAAT | | 64 | | 69 | | 5 | |  |
| TaDUF966-8A | CAAT-box | | CAAT | | 276 | | 280 | | 4 | |  |
| TaDUF966-8A | CAAT-box | | CAAT | | 326 | | 330 | | 4 | |  |
| TaDUF966-8A | CAAT-box | | CAAT | | 394 | | 398 | | 4 | |  |
| TaDUF966-8A | CAAT-box | | CAAAT | | 408 | | 413 | | 5 | |  |
| TaDUF966-8A | CAAT-box | | CAAT | | 446 | | 450 | | 4 | |  |
| TaDUF966-8A | CAAT-box | | CAAT | | 452 | | 456 | | 4 | |  |
| TaDUF966-8A | CAAT-box | | CAAT | | 485 | | 489 | | 4 | |  |
| TaDUF966-8A | CAAT-box | | CCAAT | | 487 | | 492 | | 5 | |  |
| TaDUF966-8A | CAAT-box | | CAAT | | 492 | | 496 | | 4 | |  |
| TaDUF966-8A | CAAT-box | | CCAAT | | 499 | | 504 | | 5 | |  |
| TaDUF966-8A | CAAT-box | | CAAAT | | 683 | | 688 | | 5 | |  |
| TaDUF966-8A | CAAT-box | | CAAAT | | 711 | | 716 | | 5 | |  |
| TaDUF966-8A | CAAT-box | | CAAAT | | 1002 | | 1007 | | 5 | |  |
| TaDUF966-8A | CAAT-box | | CAAT | | 1005 | | 1009 | | 4 | |  |
| TaDUF966-8A | CAAT-box | | CAAAT | | 1126 | | 1131 | | 5 | |  |
| TaDUF966-8A | CAAT-box | | CAAT | | 1203 | | 1207 | | 4 | |  |
| TaDUF966-8A | CAAT-box | | CAAT | | 1348 | | 1352 | | 4 | |  |
| TaDUF966-8A | CAAT-box | | CAACCAACTCC | | 1383 | | 1393 | | 10 | |  |
| TaDUF966-8A | CAAT-box | | CAAAT | | 1385 | | 1390 | | 5 | |  |
| TaDUF966-8A | CAT-box | | GCCACT | | 1297 | | 1303 | | 6 | |  |
| TaDUF966-8A | CGTCA-motif | | CGTCA | | 626 | | 631 | | 5 | |  |
| TaDUF966-8A | TATA-box | | TATAA | | 23 | | 28 | | 5 | |  |
| TaDUF966-8A | TATA-box | | TATA | | 24 | | 28 | | 4 | |  |
| TaDUF966-8A | TATA-box | | TATA | | 132 | | 136 | | 4 | |  |
| TaDUF966-8A | TATA-box | | ATATAA | | 278 | | 284 | | 6 | |  |
| TaDUF966-8A | TATA-box | | TATA | | 279 | | 283 | | 4 | |  |
| TaDUF966-8A | TATA-box | | ATATAA | | 427 | | 433 | | 6 | |  |
| TaDUF966-8A | TATA-box | | TATA | | 428 | | 432 | | 4 | |  |
| TaDUF966-8A | TATA-box | | TATA | | 584 | | 588 | | 4 | |  |
| TaDUF966-8A | TATA-box | | TATAAGAA | | 1110 | | 1118 | | 8 | |  |
| TaDUF966-8A | TATA-box | | TATAA | | 1113 | | 1118 | | 5 | |  |
| TaDUF966-8A | TATA-box | | TATA | | 1114 | | 1118 | | 4 | |  |
| TaDUF966-8A | TATA-box | | TATA | | 1269 | | 1273 | | 4 | |  |
| TaDUF966-8A | TATA-box | | TATACA | | 1424 | | 1430 | | 6 | |  |
| TaDUF966-8A | TATA-box | | TATA | | 1426 | | 1430 | | 4 | |  |
| TaDUF966-8A | TCA-element | | CCATCTTTTT | | 216 | | 225 | | 9 | |  |
| TaDUF966-8A | TCA-element | | TCAGAAGAGG | | 353 | | 362 | | 9 | |  |
| TaDUF966-8A | TCA-element | | CCATCTTTTT | | 1180 | | 1189 | | 9 | |  |
| TaDUF966-8A | TGACG-motif | | TGACG | | 626 | | 631 | | 5 | |  |
| TaDUF966-8B | A-box | | CCGTCC | | 107 | | 113 | | 6 | |  |
| TaDUF966-8B | A-box | | CCGTCC | | 1139 | | 1145 | | 6 | |  |
| TaDUF966-8B | A-box | | CCGTCC | | 1156 | | 1162 | | 6 | |  |
| TaDUF966-8B | ABRE | | ACGTG | | 311 | | 316 | | 5 | |  |
| TaDUF966-8B | ABRE | | ACGTG | | 1210 | | 1215 | | 5 | |  |
| TaDUF966-8B | AE-box | | AGAAACAA | | 174 | | 182 | | 8 | |  |
| TaDUF966-8B | AE-box | | AGAAACTT | | 181 | | 189 | | 8 | |  |
| TaDUF966-8B | AE-box | | AGAAACAA | | 263 | | 271 | | 8 | |  |
| TaDUF966-8B | AE-box | | AGAAACAA | | 616 | | 624 | | 8 | |  |
| TaDUF966-8B | ARE | | AAACCA | | 149 | | 155 | | 6 | |  |
| TaDUF966-8B | ARE | | AAACCA | | 1412 | | 1418 | | 6 | |  |
| TaDUF966-8B | CAAT-box | | CAAT | | 99 | | 103 | | 4 | |  |
| TaDUF966-8B | CAAT-box | | CAAAT | | 274 | | 279 | | 5 | |  |
| TaDUF966-8B | CAAT-box | | CAAT | | 354 | | 358 | | 4 | |  |
| TaDUF966-8B | CAAT-box | | CCAAT | | 403 | | 408 | | 5 | |  |
| TaDUF966-8B | CAAT-box | | CAAT | | 404 | | 408 | | 4 | |  |
| TaDUF966-8B | CAAT-box | | CCAAT | | 568 | | 573 | | 5 | |  |
| TaDUF966-8B | CAAT-box | | CCAAT | | 575 | | 580 | | 5 | |  |
| TaDUF966-8B | CAAT-box | | CAAT | | 576 | | 580 | | 4 | |  |
| TaDUF966-8B | CAAT-box | | CAAT | | 624 | | 628 | | 4 | |  |
| TaDUF966-8B | CAAT-box | | CAAAT | | 658 | | 663 | | 5 | |  |
| TaDUF966-8B | CAAT-box | | CAAT | | 704 | | 708 | | 4 | |  |
| TaDUF966-8B | CAAT-box | | CAAAT | | 738 | | 743 | | 5 | |  |
| TaDUF966-8B | CAAT-box | | CCAAT | | 769 | | 774 | | 5 | |  |
| TaDUF966-8B | CAAT-box | | CAAT | | 770 | | 774 | | 4 | |  |
| TaDUF966-8B | CAAT-box | | CAAT | | 902 | | 906 | | 4 | |  |
| TaDUF966-8B | CAT-box | | GCCACT | | 912 | | 918 | | 6 | |  |
| TaDUF966-8B | CGTCA-motif | | CGTCA | | 309 | | 314 | | 5 | |  |
| TaDUF966-8B | CGTCA-motif | | CGTCA | | 935 | | 940 | | 5 | |  |
| TaDUF966-8B | CGTCA-motif | | CGTCA | | 1220 | | 1225 | | 5 | |  |
| TaDUF966-8B | GATA-motif | | GATAGGG | | 372 | | 379 | | 7 | |  |
| TaDUF966-8B | GATA-motif | | GATAGGA | | 480 | | 487 | | 7 | |  |
| TaDUF966-8B | G-box | | CACGTC | | 310 | | 316 | | 6 | |  |
| TaDUF966-8B | G-box | | TACGTG | | 1210 | | 1216 | | 6 | |  |
| TaDUF966-8B | O2-site | | GATGACATGG | | 987 | | 996 | | 9 | |  |
| TaDUF966-8B | Sp1 | | GGGCGG | | 649 | | 655 | | 6 | |  |
| TaDUF966-8B | Sp1 | | GGGCGG | | 765 | | 771 | | 6 | |  |
| TaDUF966-8B | Sp1 | | GGGCGG | | 1118 | | 1124 | | 6 | |  |
| TaDUF966-8B | TATA-box | | TATA | | 964 | | 968 | | 4 | |  |
| TaDUF966-8B | TATA-box | | ATATAA | | 1467 | | 1473 | | 6 | |  |
| TaDUF966-8B | TATA-box | | TATA | | 1468 | | 1472 | | 4 | |  |
| TaDUF966-8B | TGACG-motif | | TGACG | | 309 | | 314 | | 5 | |  |
| TaDUF966-8B | TGACG-motif | | TGACG | | 935 | | 940 | | 5 | |  |
| TaDUF966-8B | TGACG-motif | | TGACG | | 1220 | | 1225 | | 5 | |  |
| TaDUF966-8D | A-box | | CCGTCC | | 241 | | 247 | | 6 | |  |
| TaDUF966-8D | A-box | | CCGTCC | | 1168 | | 1174 | | 6 | |  |
| TaDUF966-8D | A-box | | CCGTCC | | 1180 | | 1186 | | 6 | |  |
| TaDUF966-8D | ABRE | | CACGTG | | 311 | | 317 | | 6 | |  |
| TaDUF966-8D | ABRE | | ACGTG | | 312 | | 317 | | 5 | |  |
| TaDUF966-8D | ABRE | | ACGTG | | 1224 | | 1229 | | 5 | |  |
| TaDUF966-8D | ABRE | | ACGTG | | 1237 | | 1242 | | 5 | |  |
| TaDUF966-8D | AE-box | | AGAAACAA | | 179 | | 187 | | 8 | |  |
| TaDUF966-8D | AE-box | | AGAAACAA | | 350 | | 358 | | 8 | |  |
| TaDUF966-8D | AE-box | | AGAAACAA | | 696 | | 704 | | 8 | |  |
| TaDUF966-8D | ARE | | AAACCA | | 154 | | 160 | | 6 | |  |
| TaDUF966-8D | CAAT-box | | CAAAT | | 276 | | 281 | | 5 | |  |
| TaDUF966-8D | CAAT-box | | CAAT | | 441 | | 445 | | 4 | |  |
| TaDUF966-8D | CAAT-box | | CCAAT | | 490 | | 495 | | 5 | |  |
| TaDUF966-8D | CAAT-box | | CAAT | | 491 | | 495 | | 4 | |  |
| TaDUF966-8D | CAAT-box | | CAAT | | 704 | | 708 | | 4 | |  |
| TaDUF966-8D | CAAT-box | | CAAAT | | 730 | | 735 | | 5 | |  |
| TaDUF966-8D | CAAT-box | | CAAAT | | 768 | | 773 | | 5 | |  |
| TaDUF966-8D | CAAT-box | | CCAAT | | 799 | | 804 | | 5 | |  |
| TaDUF966-8D | CAAT-box | | CAAT | | 800 | | 804 | | 4 | |  |
| TaDUF966-8D | CAAT-box | | CAAT | | 939 | | 943 | | 4 | |  |
| TaDUF966-8D | CAT-box | | GCCACT | | 949 | | 955 | | 6 | |  |
| TaDUF966-8D | CGTCA-motif | | CGTCA | | 972 | | 977 | | 5 | |  |
| TaDUF966-8D | CGTCA-motif | | CGTCA | | 1247 | | 1252 | | 5 | |  |
| TaDUF966-8D | CGTCA-motif | | CGTCA | | 1343 | | 1348 | | 5 | |  |
| TaDUF966-8D | GATA-motif | | GATAGGA | | 567 | | 574 | | 7 | |  |
| TaDUF966-8D | G-Box | | CACGTG | | 311 | | 317 | | 6 | |  |
| TaDUF966-8D | G-box | | CACGTG | | 311 | | 317 | | 6 | |  |
| TaDUF966-8D | G-box | | CACGTC | | 1223 | | 1229 | | 6 | |  |
| TaDUF966-8D | G-box | | TACGTG | | 1237 | | 1243 | | 6 | |  |
| TaDUF966-8D | LTR | | CCGAAA | | 359 | | 365 | | 6 | |  |
| TaDUF966-8D | O2-site | | GATGACATGG | | 1024 | | 1033 | | 9 | |  |
| TaDUF966-8D | Sp1 | | GGGCGG | | 112 | | 118 | | 6 | |  |
| TaDUF966-8D | Sp1 | | GGGCGG | | 721 | | 727 | | 6 | |  |
| TaDUF966-8D | Sp1 | | GGGCGG | | 795 | | 801 | | 6 | |  |
| TaDUF966-8D | Sp1 | | GGGCGG | | 1298 | | 1304 | | 6 | |  |
| TaDUF966-8D | TATA-box | | TATA | | 1001 | | 1005 | | 4 | |  |
| TaDUF966-8D | TATA-box | | ATATAA | | 1472 | | 1478 | | 6 | |  |
| TaDUF966-8D | TATA-box | | TATA | | 1473 | | 1477 | | 4 | |  |
| TaDUF966-8D | TGACG-motif | | TGACG | | 972 | | 977 | | 5 | |  |
| TaDUF966-8D | TGACG-motif | | TGACG | | 1247 | | 1252 | | 5 | |  |
| TaDUF966-8D | TGACG-motif | | TGACG | | 1343 | | 1348 | | 5 | |  |
| TaDUF966-9A | A-box | | CCGTCC | | 275 | | 281 | | 6 | |  |
| TaDUF966-9A | A-box | | CCGTCC | | 279 | | 285 | | 6 | |  |
| TaDUF966-9A | A-box | | CCGTCC | | 283 | | 289 | | 6 | |  |
| TaDUF966-9A | A-box | | CCGTCC | | 287 | | 293 | | 6 | |  |
| TaDUF966-9A | A-box | | CCGTCC | | 376 | | 382 | | 6 | |  |
| TaDUF966-9A | A-box | | CCGTCC | | 462 | | 468 | | 6 | |  |
| TaDUF966-9A | A-box | | CCGTCC | | 704 | | 710 | | 6 | |  |
| TaDUF966-9A | A-box | | CCGTCC | | 1320 | | 1326 | | 6 | |  |
| TaDUF966-9A | ABRE | | ACGTG | | 435 | | 440 | | 5 | |  |
| TaDUF966-9A | Box 4 | | ATTAAT | | 601 | | 607 | | 6 | |  |
| TaDUF966-9A | CAAT-box | | CCAAT | | 134 | | 139 | | 5 | |  |
| TaDUF966-9A | CAAT-box | | CAAT | | 135 | | 139 | | 4 | |  |
| TaDUF966-9A | CAAT-box | | CCAAT | | 158 | | 163 | | 5 | |  |
| TaDUF966-9A | CAAT-box | | CAAT | | 159 | | 163 | | 4 | |  |
| TaDUF966-9A | CAAT-box | | CAAT | | 196 | | 200 | | 4 | |  |
| TaDUF966-9A | CAAT-box | | CAAT | | 580 | | 584 | | 4 | |  |
| TaDUF966-9A | CAAT-box | | CAAT | | 806 | | 810 | | 4 | |  |
| TaDUF966-9A | CAAT-box | | CAAT | | 827 | | 831 | | 4 | |  |
| TaDUF966-9A | CAAT-box | | CCAAT | | 941 | | 946 | | 5 | |  |
| TaDUF966-9A | CAAT-box | | CAAT | | 942 | | 946 | | 4 | |  |
| TaDUF966-9A | CAAT-box | | CAAT | | 1007 | | 1011 | | 4 | |  |
| TaDUF966-9A | CAAT-box | | CAAT | | 1122 | | 1126 | | 4 | |  |
| TaDUF966-9A | CAAT-box | | CCAAT | | 1273 | | 1278 | | 5 | |  |
| TaDUF966-9A | CAAT-box | | CAAT | | 1274 | | 1278 | | 4 | |  |
| TaDUF966-9A | CAAT-box | | CAAT | | 1408 | | 1412 | | 4 | |  |
| TaDUF966-9A | CAAT-box | | CAAT | | 1457 | | 1461 | | 4 | |  |
| TaDUF966-9A | CGTCA-motif | | CGTCA | | 230 | | 235 | | 5 | |  |
| TaDUF966-9A | CGTCA-motif | | CGTCA | | 249 | | 254 | | 5 | |  |
| TaDUF966-9A | CGTCA-motif | | CGTCA | | 413 | | 418 | | 5 | |  |
| TaDUF966-9A | CGTCA-motif | | CGTCA | | 1410 | | 1415 | | 5 | |  |
| TaDUF966-9A | G-box | | TACGTG | | 435 | | 441 | | 6 | |  |
| TaDUF966-9A | GC-motif | | CCCCCG | | 499 | | 505 | | 6 | |  |
| TaDUF966-9A | GC-motif | | CCCCCG | | 1493 | | 1499 | | 6 | |  |
| TaDUF966-9A | LTR | | CCGAAA | | 73 | | 79 | | 6 | |  |
| TaDUF966-9A | Sp1 | | GGGCGG | | 766 | | 772 | | 6 | |  |
| TaDUF966-9A | Sp1 | | GGGCGG | | 1214 | | 1220 | | 6 | |  |
| TaDUF966-9A | Sp1 | | GGGCGG | | 1303 | | 1309 | | 6 | |  |
| TaDUF966-9A | TC-rich repeats | | GTTTTCTTAC | | 1224 | | 1233 | | 9 | |  |
| TaDUF966-9A | TGACG-motif | | TGACG | | 230 | | 235 | | 5 | |  |
| TaDUF966-9A | TGACG-motif | | TGACG | | 249 | | 254 | | 5 | |  |
| TaDUF966-9A | TGACG-motif | | TGACG | | 413 | | 418 | | 5 | |  |
| TaDUF966-9A | TGACG-motif | | TGACG | | 1410 | | 1415 | | 5 | |  |
| TaDUF966-9B | A-box | | CCGTCC | | 1193 | | 1199 | | 6 | |  |
| TaDUF966-9B | ABRE | | ACGTG | | 300 | | 305 | | 5 | |  |
| TaDUF966-9B | ABRE | | ACGTG | | 355 | | 360 | | 5 | |  |
| TaDUF966-9B | ABRE | | ACGTG | | 491 | | 496 | | 5 | |  |
| TaDUF966-9B | ABRE | | GACACGTGGC | | 817 | | 826 | | 9 | |  |
| TaDUF966-9B | ABRE | | GACACGTGGC | | 840 | | 849 | | 9 | |  |
| TaDUF966-9B | ABRE | | ACGTG | | 1224 | | 1229 | | 5 | |  |
| TaDUF966-9B | ABRE | | ACGTG | | 1302 | | 1307 | | 5 | |  |
| TaDUF966-9B | ARE | | AAACCA | | 629 | | 635 | | 6 | |  |
| TaDUF966-9B | ARE | | AAACCA | | 1381 | | 1387 | | 6 | |  |
| TaDUF966-9B | ARE | | AAACCA | | 1468 | | 1474 | | 6 | |  |
| TaDUF966-9B | CAAT-box | | CAAAT | | 741 | | 746 | | 5 | |  |
| TaDUF966-9B | CAAT-box | | CAAT | | 1209 | | 1213 | | 4 | |  |
| TaDUF966-9B | CAAT-box | | CCAAT | | 1215 | | 1220 | | 5 | |  |
| TaDUF966-9B | CAAT-box | | CAAT | | 1216 | | 1220 | | 4 | |  |
| TaDUF966-9B | CAAT-box | | CAAT | | 1235 | | 1239 | | 4 | |  |
| TaDUF966-9B | CAT-box | | GCCACT | | 821 | | 827 | | 6 | |  |
| TaDUF966-9B | CAT-box | | GCCACT | | 840 | | 846 | | 6 | |  |
| TaDUF966-9B | CCAAT-box | | CAACGG | | 720 | | 726 | | 6 | |  |
| TaDUF966-9B | CGTCA-motif | | CGTCA | | 561 | | 566 | | 5 | |  |
| TaDUF966-9B | G-Box | | CACGTT | | 299 | | 305 | | 6 | |  |
| TaDUF966-9B | G-Box | | CACGTT | | 490 | | 496 | | 6 | |  |
| TaDUF966-9B | G-Box | | CACGTT | | 1224 | | 1230 | | 6 | |  |
| TaDUF966-9B | G-Box | | CACGTT | | 1301 | | 1307 | | 6 | |  |
| TaDUF966-9B | G-box | | CACGTC | | 354 | | 360 | | 6 | |  |
| TaDUF966-9B | LTR | | CCGAAA | | 152 | | 158 | | 6 | |  |
| TaDUF966-9B | LTR | | CCGAAA | | 220 | | 226 | | 6 | |  |
| TaDUF966-9B | LTR | | CCGAAA | | 475 | | 481 | | 6 | |  |
| TaDUF966-9B | MBS | | CAACTG | | 549 | | 555 | | 6 | |  |
| TaDUF966-9B | MBS | | CAACTG | | 1396 | | 1402 | | 6 | |  |
| TaDUF966-9B | MBS | | CAACTG | | 1439 | | 1445 | | 6 | |  |
| TaDUF966-9B | TATA-box | | TATAA | | 1058 | | 1063 | | 5 | |  |
| TaDUF966-9B | TATA-box | | TATA | | 1059 | | 1063 | | 4 | |  |
| TaDUF966-9B | TATA-box | | ATATAT | | 1237 | | 1243 | | 6 | |  |
| TaDUF966-9B | TATA-box | | TATA | | 1238 | | 1242 | | 4 | |  |
| TaDUF966-9B | TATA-box | | TATA | | 1492 | | 1496 | | 4 | |  |
| TaDUF966-9B | TGACG-motif | | TGACG | | 561 | | 566 | | 5 | |  |
| TaDUF966-9D | ABRE | | CGCACGTGTC | | 150 | | 159 | | 9 | |  |
| TaDUF966-9D | ABRE | | CACGTG | | 152 | | 158 | | 6 | |  |
| TaDUF966-9D | ABRE | | ACGTG | | 153 | | 158 | | 5 | |  |
| TaDUF966-9D | ABRE | | ACGTG | | 827 | | 832 | | 5 | |  |
| TaDUF966-9D | ABRE | | CACGTG | | 857 | | 863 | | 6 | |  |
| TaDUF966-9D | ABRE | | ACGTG | | 858 | | 863 | | 5 | |  |
| TaDUF966-9D | ABRE | | ACGTG | | 1165 | | 1170 | | 5 | |  |
| TaDUF966-9D | ARE | | AAACCA | | 1188 | | 1194 | | 6 | |  |
| TaDUF966-9D | CAAT-box | | CAAAT | | 11 | | 16 | | 5 | |  |
| TaDUF966-9D | CAAT-box | | CAAT | | 161 | | 165 | | 4 | |  |
| TaDUF966-9D | CAAT-box | | CAAT | | 394 | | 398 | | 4 | |  |
| TaDUF966-9D | CAAT-box | | CAAAT | | 864 | | 869 | | 5 | |  |
| TaDUF966-9D | CAAT-box | | CAAT | | 954 | | 958 | | 4 | |  |
| TaDUF966-9D | CAAT-box | | CAAT | | 968 | | 972 | | 4 | |  |
| TaDUF966-9D | CAAT-box | | CAAT | | 997 | | 1001 | | 4 | |  |
| TaDUF966-9D | CAAT-box | | CAAT | | 1015 | | 1019 | | 4 | |  |
| TaDUF966-9D | CAAT-box | | CAAT | | 1039 | | 1043 | | 4 | |  |
| TaDUF966-9D | CAAT-box | | CAAAT | | 1134 | | 1139 | | 5 | |  |
| TaDUF966-9D | CAAT-box | | CAAAT | | 1227 | | 1232 | | 5 | |  |
| TaDUF966-9D | CAAT-box | | CAAAT | | 1251 | | 1256 | | 5 | |  |
| TaDUF966-9D | CAAT-box | | CAAT | | 1320 | | 1324 | | 4 | |  |
| TaDUF966-9D | CAAT-box | | CAAT | | 1435 | | 1439 | | 4 | |  |
| TaDUF966-9D | CCAAT-box | | CAACGG | | 77 | | 83 | | 6 | |  |
| TaDUF966-9D | CGTCA-motif | | CGTCA | | 1041 | | 1046 | | 5 | |  |
| TaDUF966-9D | G-Box | | CACGTG | | 152 | | 158 | | 6 | |  |
| TaDUF966-9D | G-Box | | CACGTT | | 826 | | 832 | | 6 | |  |
| TaDUF966-9D | G-Box | | CACGTG | | 857 | | 863 | | 6 | |  |
| TaDUF966-9D | G-box | | ACACGTGT | | 151 | | 159 | | 8 | |  |
| TaDUF966-9D | G-box | | CACGTG | | 152 | | 158 | | 6 | |  |
| TaDUF966-9D | G-box | | CACGTG | | 857 | | 863 | | 6 | |  |
| TaDUF966-9D | G-box | | TACGTG | | 1165 | | 1171 | | 6 | |  |
| TaDUF966-9D | GT1-motif | | GGTTAA | | 1026 | | 1032 | | 6 | |  |
| TaDUF966-9D | MBS | | CAACTG | | 985 | | 991 | | 6 | |  |
| TaDUF966-9D | MBS | | CAACTG | | 1363 | | 1369 | | 6 | |  |
| TaDUF966-9D | TATA-box | | TATAAAA | | 321 | | 328 | | 7 | |  |
| TaDUF966-9D | TATA-box | | TATAAA | | 322 | | 328 | | 6 | |  |
| TaDUF966-9D | TATA-box | | TATAA | | 323 | | 328 | | 5 | |  |
| TaDUF966-9D | TATA-box | | TATA | | 324 | | 328 | | 4 | |  |
| TaDUF966-9D | TATA-box | | ATTATA | | 337 | | 343 | | 6 | |  |
| TaDUF966-9D | TATA-box | | TATAA | | 338 | | 343 | | 5 | |  |
| TaDUF966-9D | TATA-box | | TATA | | 339 | | 343 | | 4 | |  |
| TaDUF966-9D | TATA-box | | ATTATA | | 405 | | 411 | | 6 | |  |
| TaDUF966-9D | TATA-box | | TATAA | | 406 | | 411 | | 5 | |  |
| TaDUF966-9D | TATA-box | | TATA | | 407 | | 411 | | 4 | |  |
| TaDUF966-9D | TATA-box | | ATTATA | | 433 | | 439 | | 6 | |  |
| TaDUF966-9D | TATA-box | | TATAA | | 434 | | 439 | | 5 | |  |
| TaDUF966-9D | TATA-box | | TATA | | 435 | | 439 | | 4 | |  |
| TaDUF966-9D | TATA-box | | ATTATA | | 750 | | 756 | | 6 | |  |
| TaDUF966-9D | TATA-box | | TATAA | | 751 | | 756 | | 5 | |  |
| TaDUF966-9D | TATA-box | | TATA | | 752 | | 756 | | 4 | |  |
| TaDUF966-9D | TATA-box | | TACAAAA | | 891 | | 898 | | 7 | |  |
| TaDUF966-9D | TATA-box | | ATATAA | | 913 | | 919 | | 6 | |  |
| TaDUF966-9D | TATA-box | | TATA | | 914 | | 918 | | 4 | |  |
| TaDUF966-9D | TATA-box | | TATA | | 1007 | | 1011 | | 4 | |  |
| TaDUF966-9D | TATA-box | | TAAAGATT | | 1100 | | 1108 | | 8 | |  |
| TaDUF966-9D | TATA-box | | ATTATA | | 1404 | | 1410 | | 6 | |  |
| TaDUF966-9D | TATA-box | | TATAA | | 1405 | | 1410 | | 5 | |  |
| TaDUF966-9D | TATA-box | | TATA | | 1406 | | 1410 | | 4 | |  |
| TaDUF966-9D | TATA-box | | TATATA | | 1454 | | 1460 | | 6 | |  |
| TaDUF966-9D | TATA-box | | ATATAT | | 1455 | | 1461 | | 6 | |  |
| TaDUF966-9D | TATA-box | | TATATA | | 1456 | | 1462 | | 6 | |  |
| TaDUF966-9D | TATA-box | | ATATAA | | 1457 | | 1463 | | 6 | |  |
| TaDUF966-9D | TATA-box | | TATA | | 1458 | | 1462 | | 4 | |  |
| TaDUF966-9D | TGACG-motif | | TGACG | | 1041 | | 1046 | | 5 | |  |
| TaDUF966-9D | TGA-element | | AACGAC | | 1425 | | 1431 | | 6 | |  |
| TaDUF966-10A | ABRE | | ACGTG | | 3 | | 8 | | 5 | |  |
| TaDUF966-10A | ABRE | | CACGTG | | 665 | | 671 | | 6 | |  |
| TaDUF966-10A | ABRE | | ACGTG | | 666 | | 671 | | 5 | |  |
| TaDUF966-10A | ABRE | | CGTACGTGCA | | 1207 | | 1216 | | 9 | |  |
| TaDUF966-10A | ABRE | | ACGTG | | 1209 | | 1214 | | 5 | |  |
| TaDUF966-10A | CAAT-box | | CAAT | | 40 | | 44 | | 4 | |  |
| TaDUF966-10A | CAAT-box | | CAAT | | 184 | | 188 | | 4 | |  |
| TaDUF966-10A | CAAT-box | | CAAAT | | 190 | | 195 | | 5 | |  |
| TaDUF966-10A | CAAT-box | | CAAAT | | 360 | | 365 | | 5 | |  |
| TaDUF966-10A | CAAT-box | | CAAAT | | 447 | | 452 | | 5 | |  |
| TaDUF966-10A | CAAT-box | | CAAT | | 482 | | 486 | | 4 | |  |
| TaDUF966-10A | CAAT-box | | CAAT | | 518 | | 522 | | 4 | |  |
| TaDUF966-10A | CAAT-box | | CAAT | | 555 | | 559 | | 4 | |  |
| TaDUF966-10A | CAAT-box | | CAAT | | 603 | | 607 | | 4 | |  |
| TaDUF966-10A | CAAT-box | | CCAAT | | 653 | | 658 | | 5 | |  |
| TaDUF966-10A | CAAT-box | | CAAT | | 654 | | 658 | | 4 | |  |
| TaDUF966-10A | CAAT-box | | CCAAT | | 774 | | 779 | | 5 | |  |
| TaDUF966-10A | CAAT-box | | CAAT | | 775 | | 779 | | 4 | |  |
| TaDUF966-10A | CAAT-box | | CAACCAACTCC | | 785 | | 795 | | 10 | |  |
| TaDUF966-10A | CAAT-box | | CAAAT | | 850 | | 855 | | 5 | |  |
| TaDUF966-10A | CAAT-box | | CAAT | | 958 | | 962 | | 4 | |  |
| TaDUF966-10A | CAAT-box | | CAAT | | 1096 | | 1100 | | 4 | |  |
| TaDUF966-10A | CAAT-box | | CAAT | | 1217 | | 1221 | | 4 | |  |
| TaDUF966-10A | CAAT-box | | CAAAT | | 1349 | | 1354 | | 5 | |  |
| TaDUF966-10A | CAAT-box | | CAAAT | | 1427 | | 1432 | | 5 | |  |
| TaDUF966-10A | CAT-box | | GCCACT | | 931 | | 937 | | 6 | |  |
| TaDUF966-10A | CAT-box | | GCCACT | | 1309 | | 1315 | | 6 | |  |
| TaDUF966-10A | CCAAT-box | | CAACGG | | 236 | | 242 | | 6 | |  |
| TaDUF966-10A | CCAAT-box | | CAACGG | | 253 | | 259 | | 6 | |  |
| TaDUF966-10A | CGTCA-motif | | CGTCA | | 1111 | | 1116 | | 5 | |  |
| TaDUF966-10A | GATA-motif | | GATAGGA | | 567 | | 574 | | 7 | |  |
| TaDUF966-10A | G-Box | | CACGTG | | 665 | | 671 | | 6 | |  |
| TaDUF966-10A | G-box | | TACGTG | | 2 | | 8 | | 6 | |  |
| TaDUF966-10A | G-box | | CACGTG | | 665 | | 671 | | 6 | |  |
| TaDUF966-10A | G-box | | TACGTG | | 1209 | | 1215 | | 6 | |  |
| TaDUF966-10A | I-box | | AAGATAAGGCT | | 488 | | 498 | | 10 | |  |
| TaDUF966-10A | I-box | | AGATAAGG | | 490 | | 498 | | 8 | |  |
| TaDUF966-10A | I-box | | gGATAAGGTG | | 755 | | 764 | | 9 | |  |
| TaDUF966-10A | LTR | | CCGAAA | | 32 | | 38 | | 6 | |  |
| TaDUF966-10A | P-box | | CCTTTTG | | 860 | | 867 | | 7 | |  |
| TaDUF966-10A | TATA-box | | TATA | | 1173 | | 1177 | | 4 | |  |
| TaDUF966-10A | TGACG-motif | | TGACG | | 1111 | | 1116 | | 5 | |  |
| TaDUF966-10B | A-box | | CCGTCC | | 1227 | | 1233 | | 6 | |  |
| TaDUF966-10B | ABRE | | CACGTG | | 216 | | 222 | | 6 | |  |
| TaDUF966-10B | ABRE | | ACGTG | | 217 | | 222 | | 5 | |  |
| TaDUF966-10B | AE-box | | AGAAACTT | | 1457 | | 1465 | | 8 | |  |
| TaDUF966-10B | ARE | | AAACCA | | 1210 | | 1216 | | 6 | |  |
| TaDUF966-10B | ARE | | AAACCA | | 1382 | | 1388 | | 6 | |  |
| TaDUF966-10B | CAAT-box | | CAAT | | 8 | | 12 | | 4 | |  |
| TaDUF966-10B | CAAT-box | | CCAAT | | 91 | | 96 | | 5 | |  |
| TaDUF966-10B | CAAT-box | | CCAAT | | 159 | | 164 | | 5 | |  |
| TaDUF966-10B | CAAT-box | | CCAAT | | 180 | | 185 | | 5 | |  |
| TaDUF966-10B | CAAT-box | | CCAAT | | 204 | | 209 | | 5 | |  |
| TaDUF966-10B | CAAT-box | | CAAT | | 205 | | 209 | | 4 | |  |
| TaDUF966-10B | CAAT-box | | CAACCAACTCC | | 296 | | 306 | | 10 | |  |
| TaDUF966-10B | CAAT-box | | CAAT | | 338 | | 342 | | 4 | |  |
| TaDUF966-10B | CAAT-box | | CAAAT | | 355 | | 360 | | 5 | |  |
| TaDUF966-10B | CAAT-box | | CAAT | | 481 | | 485 | | 4 | |  |
| TaDUF966-10B | CAAT-box | | CAAT | | 625 | | 629 | | 4 | |  |
| TaDUF966-10B | CAAT-box | | CCAAT | | 808 | | 813 | | 5 | |  |
| TaDUF966-10B | CAAT-box | | CAAT | | 809 | | 813 | | 4 | |  |
| TaDUF966-10B | CAAT-box | | CAAT | | 843 | | 847 | | 4 | |  |
| TaDUF966-10B | CAAT-box | | CAAAT | | 1080 | | 1085 | | 5 | |  |
| TaDUF966-10B | CAAT-box | | CAAT | | 1150 | | 1154 | | 4 | |  |
| TaDUF966-10B | CAAT-box | | CAAT | | 1220 | | 1224 | | 4 | |  |
| TaDUF966-10B | CAAT-box | | CAAT | | 1264 | | 1268 | | 4 | |  |
| TaDUF966-10B | CAAT-box | | CAAT | | 1311 | | 1315 | | 4 | |  |
| TaDUF966-10B | CAAT-box | | CAAAT | | 1326 | | 1331 | | 5 | |  |
| TaDUF966-10B | CAAT-box | | CAAT | | 1443 | | 1447 | | 4 | |  |
| TaDUF966-10B | CAAT-box | | CAAAT | | 1447 | | 1452 | | 5 | |  |
| TaDUF966-10B | CAT-box | | GCCACT | | 942 | | 948 | | 6 | |  |
| TaDUF966-10B | CGTCA-motif | | CGTCA | | 36 | | 41 | | 5 | |  |
| TaDUF966-10B | CGTCA-motif | | CGTCA | | 499 | | 504 | | 5 | |  |
| TaDUF966-10B | CGTCA-motif | | CGTCA | | 1259 | | 1264 | | 5 | |  |
| TaDUF966-10B | GATA-motif | | GATAGGA | | 20 | | 27 | | 7 | |  |
| TaDUF966-10B | GATA-motif | | GATAGGA | | 314 | | 321 | | 7 | |  |
| TaDUF966-10B | G-box | | CACGTG | | 216 | | 222 | | 6 | |  |
| TaDUF966-10B | G-Box | | CACGTG | | 216 | | 222 | | 6 | |  |
| TaDUF966-10B | G-Box | | TCCACATGGCA | | 328 | | 338 | | 10 | |  |
| TaDUF966-10B | GT1-motif | | GGTTAAT | | 811 | | 818 | | 7 | |  |
| TaDUF966-10B | GT1-motif | | GGTTAA | | 812 | | 818 | | 6 | |  |
| TaDUF966-10B | Sp1 | | GGGCGG | | 517 | | 523 | | 6 | |  |
| TaDUF966-10B | Sp1 | | GGGCGG | | 1160 | | 1166 | | 6 | |  |
| TaDUF966-10B | TATA-box | | ATATAT | | 117 | | 123 | | 6 | |  |
| TaDUF966-10B | TATA-box | | TATA | | 118 | | 122 | | 4 | |  |
| TaDUF966-10B | TATA-box | | TACAAAA | | 616 | | 623 | | 7 | |  |
| TaDUF966-10B | TATA-box | | TATAA | | 657 | | 662 | | 5 | |  |
| TaDUF966-10B | TATA-box | | TATA | | 658 | | 662 | | 4 | |  |
| TaDUF966-10B | TATA-box | | ccTATAAAaa | | 704 | | 713 | | 9 | |  |
| TaDUF966-10B | TATA-box | | TACAAAA | | 706 | | 713 | | 7 | |  |
| TaDUF966-10B | TATA-box | | ATATAT | | 827 | | 833 | | 6 | |  |
| TaDUF966-10B | TATA-box | | TATA | | 828 | | 832 | | 4 | |  |
| TaDUF966-10B | TCA-element | | TCAGAAGAGG | | 70 | | 79 | | 9 | |  |
| TaDUF966-10B | TGACG-motif | | TGACG | | 36 | | 41 | | 5 | |  |
| TaDUF966-10B | TGACG-motif | | TGACG | | 499 | | 504 | | 5 | |  |
| TaDUF966-10B | TGACG-motif | | TGACG | | 1259 | | 1264 | | 5 | |  |
| TaDUF966-10D | ABRE | | CGTACGTGCA | | 302 | | 311 | | 9 | |  |
| TaDUF966-10D | ABRE | | ACGTG | | 305 | | 310 | | 5 | |  |
| TaDUF966-10D | ABRE | | ACGTG | | 739 | | 744 | | 5 | |  |
| TaDUF966-10D | ARE | | AAACCA | | 1416 | | 1422 | | 6 | |  |
| TaDUF966-10D | Box 4 | | ATTAAT | | 1102 | | 1108 | | 6 | |  |
| TaDUF966-10D | CAAT-box | | CAAT | | 149 | | 153 | | 4 | |  |
| TaDUF966-10D | CAAT-box | | CAAAT | | 221 | | 226 | | 5 | |  |
| TaDUF966-10D | CAAT-box | | CAAT | | 241 | | 245 | | 4 | |  |
| TaDUF966-10D | CAAT-box | | CAAT | | 427 | | 431 | | 4 | |  |
| TaDUF966-10D | CAAT-box | | CAAAT | | 542 | | 547 | | 5 | |  |
| TaDUF966-10D | CAAT-box | | CAAT | | 545 | | 549 | | 4 | |  |
| TaDUF966-10D | CAAT-box | | CAAT | | 624 | | 628 | | 4 | |  |
| TaDUF966-10D | CAAT-box | | CAAT | | 693 | | 697 | | 4 | |  |
| TaDUF966-10D | CAAT-box | | CAAAT | | 725 | | 730 | | 5 | |  |
| TaDUF966-10D | CAAT-box | | CAAT | | 794 | | 798 | | 4 | |  |
| TaDUF966-10D | CAAT-box | | CAAT | | 871 | | 875 | | 4 | |  |
| TaDUF966-10D | CAAT-box | | CAAT | | 910 | | 914 | | 4 | |  |
| TaDUF966-10D | CAAT-box | | CCAAT | | 1031 | | 1036 | | 5 | |  |
| TaDUF966-10D | CAAT-box | | CCAAT | | 1038 | | 1043 | | 5 | |  |
| TaDUF966-10D | CAAT-box | | CAAAT | | 1075 | | 1080 | | 5 | |  |
| TaDUF966-10D | CAAT-box | | CCAAT | | 1164 | | 1169 | | 5 | |  |
| TaDUF966-10D | CAAT-box | | CAAT | | 1224 | | 1228 | | 4 | |  |
| TaDUF966-10D | CAAT-box | | CAAAT | | 1251 | | 1256 | | 5 | |  |
| TaDUF966-10D | CAAT-box | | CAAAT | | 1428 | | 1433 | | 5 | |  |
| TaDUF966-10D | CAT-box | | GCCACT | | 780 | | 786 | | 6 | |  |
| TaDUF966-10D | CAT-box | | GCCACT | | 1025 | | 1031 | | 6 | |  |
| TaDUF966-10D | CGTCA-motif | | CGTCA | | 424 | | 429 | | 5 | |  |
| TaDUF966-10D | G-box | | CACGAC | | 281 | | 287 | | 6 | |  |
| TaDUF966-10D | G-box | | TACGTG | | 304 | | 310 | | 6 | |  |
| TaDUF966-10D | G-box | | CACGTC | | 738 | | 744 | | 6 | |  |
| TaDUF966-10D | GT1-motif | | GGTTAA | | 786 | | 792 | | 6 | |  |
| TaDUF966-10D | GT1-motif | | GTGTGTGAA | | 1197 | | 1206 | | 9 | |  |
| TaDUF966-10D | I-box | | gGATAAGGTG | | 36 | | 45 | | 9 | |  |
| TaDUF966-10D | MBS | | CAACTG | | 1478 | | 1484 | | 6 | |  |
| TaDUF966-10D | P-box | | CCTTTTG | | 258 | | 265 | | 7 | |  |
| TaDUF966-10D | P-box | | CCTTTTG | | 769 | | 776 | | 7 | |  |
| TaDUF966-10D | TATA-box | | TATACA | | 47 | | 53 | | 6 | |  |
| TaDUF966-10D | TATA-box | | TATATA | | 49 | | 55 | | 6 | |  |
| TaDUF966-10D | TATA-box | | TATA | | 51 | | 55 | | 4 | |  |
| TaDUF966-10D | TATA-box | | TATACA | | 156 | | 162 | | 6 | |  |
| TaDUF966-10D | TATA-box | | TATA | | 158 | | 162 | | 4 | |  |
| TaDUF966-10D | TATA-box | | TATAA | | 833 | | 838 | | 5 | |  |
| TaDUF966-10D | TATA-box | | TATA | | 834 | | 838 | | 4 | |  |
| TaDUF966-10D | TATA-box | | TAAAGATT | | 905 | | 913 | | 8 | |  |
| TaDUF966-10D | TCA-element | | CCATCTTTTT | | 1336 | | 1345 | | 9 | |  |
| TaDUF966-10D | TGACG-motif | | TGACG | | 424 | | 429 | | 5 | |  |
| TaDUF966-11B | AE-box | | AGAAACTT | | 109 | | 117 | | 8 | |  |
| TaDUF966-11B | ARE | | AAACCA | | 1129 | | 1135 | | 6 | |  |
| TaDUF966-11B | CAAT-box | | CAAT | | 43 | | 47 | | 4 | |  |
| TaDUF966-11B | CAAT-box | | CAAAT | | 166 | | 171 | | 5 | |  |
| TaDUF966-11B | CAAT-box | | CAAT | | 212 | | 216 | | 4 | |  |
| TaDUF966-11B | CAAT-box | | CAAT | | 233 | | 237 | | 4 | |  |
| TaDUF966-11B | CAAT-box | | CCAAT | | 268 | | 273 | | 5 | |  |
| TaDUF966-11B | CAAT-box | | CAAT | | 269 | | 273 | | 4 | |  |
| TaDUF966-11B | CAAT-box | | CAAAT | | 283 | | 288 | | 5 | |  |
| TaDUF966-11B | CAAT-box | | CCAAT | | 314 | | 319 | | 5 | |  |
| TaDUF966-11B | CAAT-box | | CAAT | | 315 | | 319 | | 4 | |  |
| TaDUF966-11B | CAAT-box | | CAAAT | | 335 | | 340 | | 5 | |  |
| TaDUF966-11B | CAAT-box | | CCAAT | | 376 | | 381 | | 5 | |  |
| TaDUF966-11B | CAAT-box | | CAAT | | 377 | | 381 | | 4 | |  |
| TaDUF966-11B | CAAT-box | | CAAT | | 446 | | 450 | | 4 | |  |
| TaDUF966-11B | CAAT-box | | CAAAT | | 619 | | 624 | | 5 | |  |
| TaDUF966-11B | CAAT-box | | CAAT | | 695 | | 699 | | 4 | |  |
| TaDUF966-11B | CAAT-box | | CAAT | | 762 | | 766 | | 4 | |  |
| TaDUF966-11B | CAAT-box | | CCAAT | | 855 | | 860 | | 5 | |  |
| TaDUF966-11B | CAAT-box | | CAAT | | 856 | | 860 | | 4 | |  |
| TaDUF966-11B | CAAT-box | | CAAAT | | 919 | | 924 | | 5 | |  |
| TaDUF966-11B | CAAT-box | | CAAT | | 1037 | | 1041 | | 4 | |  |
| TaDUF966-11B | CAAT-box | | CAAT | | 1043 | | 1047 | | 4 | |  |
| TaDUF966-11B | CAAT-box | | CAAT | | 1098 | | 1102 | | 4 | |  |
| TaDUF966-11B | CAAT-box | | CCAAT | | 1132 | | 1137 | | 5 | |  |
| TaDUF966-11B | CAAT-box | | CAAT | | 1133 | | 1137 | | 4 | |  |
| TaDUF966-11B | CAAT-box | | CAAT | | 1159 | | 1163 | | 4 | |  |
| TaDUF966-11B | CAAT-box | | CAAT | | 1242 | | 1246 | | 4 | |  |
| TaDUF966-11B | CAAT-box | | CAAT | | 1326 | | 1330 | | 4 | |  |
| TaDUF966-11B | CAAT-box | | CAAT | | 1360 | | 1364 | | 4 | |  |
| TaDUF966-11B | CAAT-box | | CAAT | | 1381 | | 1385 | | 4 | |  |
| TaDUF966-11B | CAAT-box | | CAAT | | 1426 | | 1430 | | 4 | |  |
| TaDUF966-11B | CAAT-box | | CCAAT | | 1428 | | 1433 | | 5 | |  |
| TaDUF966-11B | CAAT-box | | CCAAT | | 1463 | | 1468 | | 5 | |  |
| TaDUF966-11B | CAAT-box | | CAAT | | 1464 | | 1468 | | 4 | |  |
| TaDUF966-11B | CAT-box | | GCCACT | | 1225 | | 1231 | | 6 | |  |
| TaDUF966-11B | CGTCA-motif | | CGTCA | | 123 | | 128 | | 5 | |  |
| TaDUF966-11B | CGTCA-motif | | CGTCA | | 1486 | | 1491 | | 5 | |  |
| TaDUF966-11B | GATA-motif | | AAGATAAGATT | | 390 | | 400 | | 10 | |  |
| TaDUF966-11B | GATA-motif | | GATAGGA | | 392 | | 399 | | 7 | |  |
| TaDUF966-11B | GATA-motif | | GATAGGG | | 904 | | 911 | | 7 | |  |
| TaDUF966-11B | G-box | | CACGAC | | 1270 | | 1276 | | 6 | |  |
| TaDUF966-11B | GT1-motif | | GGTTAA | | 1191 | | 1197 | | 6 | |  |
| TaDUF966-11B | MBS | | CAACTG | | 196 | | 202 | | 6 | |  |
| TaDUF966-11B | MBS | | CAACTG | | 529 | | 535 | | 6 | |  |
| TaDUF966-11B | P-box | | CCTTTTG | | 452 | | 459 | | 7 | |  |
| TaDUF966-11B | TATA-box | | TATA | | 174 | | 178 | | 4 | |  |
| TaDUF966-11B | TATA-box | | TATACA | | 295 | | 301 | | 6 | |  |
| TaDUF966-11B | TATA-box | | TATA | | 297 | | 301 | | 4 | |  |
| TaDUF966-11B | TATA-box | | TATA | | 339 | | 343 | | 4 | |  |
| TaDUF966-11B | TATA-box | | TATACA | | 347 | | 353 | | 6 | |  |
| TaDUF966-11B | TATA-box | | TATA | | 349 | | 353 | | 4 | |  |
| TaDUF966-11B | TATA-box | | TATACA | | 357 | | 363 | | 6 | |  |
| TaDUF966-11B | TATA-box | | TATA | | 359 | | 363 | | 4 | |  |
| TaDUF966-11B | TATA-box | | ATTATA | | 398 | | 404 | | 6 | |  |
| TaDUF966-11B | TATA-box | | TATATAA | | 399 | | 406 | | 7 | |  |
| TaDUF966-11B | TATA-box | | TATATA | | 400 | | 406 | | 6 | |  |
| TaDUF966-11B | TATA-box | | TATA | | 402 | | 406 | | 4 | |  |
| TaDUF966-11B | TATA-box | | TATAA | | 422 | | 427 | | 5 | |  |
| TaDUF966-11B | TATA-box | | TATA | | 423 | | 427 | | 4 | |  |
| TaDUF966-11B | TATA-box | | TACAAAA | | 439 | | 446 | | 7 | |  |
| TaDUF966-11B | TATA-box | | TATACA | | 475 | | 481 | | 6 | |  |
| TaDUF966-11B | TATA-box | | TATA | | 477 | | 481 | | 4 | |  |
| TaDUF966-11B | TATA-box | | TATA | | 549 | | 553 | | 4 | |  |
| TaDUF966-11B | TATA-box | | TATTTAAA | | 551 | | 559 | | 8 | |  |
| TaDUF966-11B | TATA-box | | ccTATAAAaa | | 855 | | 864 | | 9 | |  |
| TaDUF966-11B | TATA-box | | ATTATA | | 980 | | 986 | | 6 | |  |
| TaDUF966-11B | TATA-box | | TATAA | | 981 | | 986 | | 5 | |  |
| TaDUF966-11B | TATA-box | | TATA | | 982 | | 986 | | 4 | |  |
| TaDUF966-11B | TATA-box | | TATACA | | 1087 | | 1093 | | 6 | |  |
| TaDUF966-11B | TATA-box | | TATATA | | 1089 | | 1095 | | 6 | |  |
| TaDUF966-11B | TATA-box | | TATA | | 1091 | | 1095 | | 4 | |  |
| TaDUF966-11B | TATA-box | | TATA | | 1351 | | 1355 | | 4 | |  |
| TaDUF966-11B | TGACG-motif | | TGACG | | 123 | | 128 | | 5 | |  |
| TaDUF966-11B | TGACG-motif | | TGACG | | 1486 | | 1491 | | 5 | |  |
| TaDUF966-12B | AE-box | | AGAAACTT | | 92 | | 100 | | 8 | |  |
| TaDUF966-12B | ARE | | AAACCA | | 1077 | | 1083 | | 6 | |  |
| TaDUF966-12B | CAAT-box | | CAAT | | 22 | | 26 | | 4 | |  |
| TaDUF966-12B | CAAT-box | | CAAAT | | 149 | | 154 | | 5 | |  |
| TaDUF966-12B | CAAT-box | | CAAT | | 195 | | 199 | | 4 | |  |
| TaDUF966-12B | CAAT-box | | CAAT | | 216 | | 220 | | 4 | |  |
| TaDUF966-12B | CAAT-box | | CCAAT | | 251 | | 256 | | 5 | |  |
| TaDUF966-12B | CAAT-box | | CAAT | | 252 | | 256 | | 4 | |  |
| TaDUF966-12B | CAAT-box | | CAAAT | | 266 | | 271 | | 5 | |  |
| TaDUF966-12B | CAAT-box | | CCAAT | | 297 | | 302 | | 5 | |  |
| TaDUF966-12B | CAAT-box | | CAAT | | 298 | | 302 | | 4 | |  |
| TaDUF966-12B | CAAT-box | | CAAAT | | 318 | | 323 | | 5 | |  |
| TaDUF966-12B | CAAT-box | | CAAT | | 429 | | 433 | | 4 | |  |
| TaDUF966-12B | CAAT-box | | CAAAT | | 602 | | 607 | | 5 | |  |
| TaDUF966-12B | CAAT-box | | CAAT | | 678 | | 682 | | 4 | |  |
| TaDUF966-12B | CAAT-box | | CAAT | | 710 | | 714 | | 4 | |  |
| TaDUF966-12B | CAAT-box | | CCAAT | | 803 | | 808 | | 5 | |  |
| TaDUF966-12B | CAAT-box | | CAAT | | 804 | | 808 | | 4 | |  |
| TaDUF966-12B | CAAT-box | | CAAAT | | 867 | | 872 | | 5 | |  |
| TaDUF966-12B | CAAT-box | | CAAT | | 903 | | 907 | | 4 | |  |
| TaDUF966-12B | CAAT-box | | CAAT | | 985 | | 989 | | 4 | |  |
| TaDUF966-12B | CAAT-box | | CAAT | | 991 | | 995 | | 4 | |  |
| TaDUF966-12B | CAAT-box | | CAAT | | 1046 | | 1050 | | 4 | |  |
| TaDUF966-12B | CAAT-box | | CCAAT | | 1080 | | 1085 | | 5 | |  |
| TaDUF966-12B | CAAT-box | | CAAT | | 1081 | | 1085 | | 4 | |  |
| TaDUF966-12B | CAAT-box | | CAAT | | 1107 | | 1111 | | 4 | |  |
| TaDUF966-12B | CAAT-box | | CAAT | | 1190 | | 1194 | | 4 | |  |
| TaDUF966-12B | CAAT-box | | CAAT | | 1274 | | 1278 | | 4 | |  |
| TaDUF966-12B | CAAT-box | | CAAT | | 1308 | | 1312 | | 4 | |  |
| TaDUF966-12B | CAAT-box | | CAAT | | 1329 | | 1333 | | 4 | |  |
| TaDUF966-12B | CAAT-box | | CAAT | | 1374 | | 1378 | | 4 | |  |
| TaDUF966-12B | CAAT-box | | CCAAT | | 1376 | | 1381 | | 5 | |  |
| TaDUF966-12B | CAAT-box | | CCAAT | | 1411 | | 1416 | | 5 | |  |
| TaDUF966-12B | CAAT-box | | CAAT | | 1412 | | 1416 | | 4 | |  |
| TaDUF966-12B | CAT-box | | GCCACT | | 1173 | | 1179 | | 6 | |  |
| TaDUF966-12B | CGTCA-motif | | CGTCA | | 106 | | 111 | | 5 | |  |
| TaDUF966-12B | GATA-motif | | AAGATAAGATT | | 373 | | 383 | | 10 | |  |
| TaDUF966-12B | GATA-motif | | GATAGGA | | 375 | | 382 | | 7 | |  |
| TaDUF966-12B | GATA-motif | | GATAGGG | | 852 | | 859 | | 7 | |  |
| TaDUF966-12B | G-box | | CACGAC | | 1218 | | 1224 | | 6 | |  |
| TaDUF966-12B | GT1-motif | | GGTTAA | | 1139 | | 1145 | | 6 | |  |
| TaDUF966-12B | MBS | | CAACTG | | 179 | | 185 | | 6 | |  |
| TaDUF966-12B | P-box | | CCTTTTG | | 435 | | 442 | | 7 | |  |
| TaDUF966-12B | TATA-box | | ATATAA | | 118 | | 124 | | 6 | |  |
| TaDUF966-12B | TATA-box | | TATA | | 119 | | 123 | | 4 | |  |
| TaDUF966-12B | TATA-box | | TATA | | 157 | | 161 | | 4 | |  |
| TaDUF966-12B | TATA-box | | TATACA | | 278 | | 284 | | 6 | |  |
| TaDUF966-12B | TATA-box | | TATA | | 280 | | 284 | | 4 | |  |
| TaDUF966-12B | TATA-box | | TATA | | 322 | | 326 | | 4 | |  |
| TaDUF966-12B | TATA-box | | TATACA | | 330 | | 336 | | 6 | |  |
| TaDUF966-12B | TATA-box | | TATA | | 332 | | 336 | | 4 | |  |
| TaDUF966-12B | TATA-box | | TATATA | | 340 | | 346 | | 6 | |  |
| TaDUF966-12B | TATA-box | | TATA | | 342 | | 346 | | 4 | |  |
| TaDUF966-12B | TATA-box | | ATTATA | | 381 | | 387 | | 6 | |  |
| TaDUF966-12B | TATA-box | | TATATAA | | 382 | | 389 | | 7 | |  |
| TaDUF966-12B | TATA-box | | TATATA | | 383 | | 389 | | 6 | |  |
| TaDUF966-12B | TATA-box | | TATA | | 385 | | 389 | | 4 | |  |
| TaDUF966-12B | TATA-box | | TATAA | | 405 | | 410 | | 5 | |  |
| TaDUF966-12B | TATA-box | | TATA | | 406 | | 410 | | 4 | |  |
| TaDUF966-12B | TATA-box | | TACAAAA | | 422 | | 429 | | 7 | |  |
| TaDUF966-12B | TATA-box | | TATACA | | 458 | | 464 | | 6 | |  |
| TaDUF966-12B | TATA-box | | TATA | | 460 | | 464 | | 4 | |  |
| TaDUF966-12B | TATA-box | | TATA | | 532 | | 536 | | 4 | |  |
| TaDUF966-12B | TATA-box | | TATTTAAA | | 534 | | 542 | | 8 | |  |
| TaDUF966-12B | TATA-box | | ccTATAAAaa | | 803 | | 812 | | 9 | |  |
| TaDUF966-12B | TATA-box | | ATTATA | | 928 | | 934 | | 6 | |  |
| TaDUF966-12B | TATA-box | | TATAA | | 929 | | 934 | | 5 | |  |
| TaDUF966-12B | TATA-box | | TATA | | 930 | | 934 | | 4 | |  |
| TaDUF966-12B | TATA-box | | TATACA | | 1035 | | 1041 | | 6 | |  |
| TaDUF966-12B | TATA-box | | TATATA | | 1037 | | 1043 | | 6 | |  |
| TaDUF966-12B | TATA-box | | TATA | | 1039 | | 1043 | | 4 | |  |
| TaDUF966-12B | TATA-box | | TATA | | 1299 | | 1303 | | 4 | |  |
| TaDUF966-12B | TGACG-motif | | TGACG | | 106 | | 111 | | 5 | |  |
| TaDUF966-13A | AE-box | | AGAAACTT | | 160 | | 168 | | 8 | |  |
| TaDUF966-13A | ARE | | AAACCA | | 1180 | | 1186 | | 6 | |  |
| TaDUF966-13A | CAAT-box | | CAAAT | | 4 | | 9 | | 5 | |  |
| TaDUF966-13A | CAAT-box | | CAAT | | 90 | | 94 | | 4 | |  |
| TaDUF966-13A | CAAT-box | | CAAAT | | 217 | | 222 | | 5 | |  |
| TaDUF966-13A | CAAT-box | | CAAT | | 263 | | 267 | | 4 | |  |
| TaDUF966-13A | CAAT-box | | CAAT | | 284 | | 288 | | 4 | |  |
| TaDUF966-13A | CAAT-box | | CCAAT | | 319 | | 324 | | 5 | |  |
| TaDUF966-13A | CAAT-box | | CAAT | | 320 | | 324 | | 4 | |  |
| TaDUF966-13A | CAAT-box | | CAAAT | | 334 | | 339 | | 5 | |  |
| TaDUF966-13A | CAAT-box | | CCAAT | | 365 | | 370 | | 5 | |  |
| TaDUF966-13A | CAAT-box | | CAAT | | 366 | | 370 | | 4 | |  |
| TaDUF966-13A | CAAT-box | | CAAAT | | 386 | | 391 | | 5 | |  |
| TaDUF966-13A | CAAT-box | | CCAAT | | 427 | | 432 | | 5 | |  |
| TaDUF966-13A | CAAT-box | | CAAT | | 428 | | 432 | | 4 | |  |
| TaDUF966-13A | CAAT-box | | CAAT | | 497 | | 501 | | 4 | |  |
| TaDUF966-13A | CAAT-box | | CAAT | | 746 | | 750 | | 4 | |  |
| TaDUF966-13A | CAAT-box | | CAAT | | 813 | | 817 | | 4 | |  |
| TaDUF966-13A | CAAT-box | | CCAAT | | 875 | | 880 | | 5 | |  |
| TaDUF966-13A | CAAT-box | | CCAAT | | 906 | | 911 | | 5 | |  |
| TaDUF966-13A | CAAT-box | | CAAT | | 907 | | 911 | | 4 | |  |
| TaDUF966-13A | CAAT-box | | CAAAT | | 970 | | 975 | | 5 | |  |
| TaDUF966-13A | CAAT-box | | CAAT | | 1006 | | 1010 | | 4 | |  |
| TaDUF966-13A | CAAT-box | | CAAT | | 1088 | | 1092 | | 4 | |  |
| TaDUF966-13A | CAAT-box | | CAAT | | 1094 | | 1098 | | 4 | |  |
| TaDUF966-13A | CAAT-box | | CAAT | | 1149 | | 1153 | | 4 | |  |
| TaDUF966-13A | CAAT-box | | CCAAT | | 1183 | | 1188 | | 5 | |  |
| TaDUF966-13A | CAAT-box | | CAAT | | 1184 | | 1188 | | 4 | |  |
| TaDUF966-13A | CAAT-box | | CAAT | | 1204 | | 1208 | | 4 | |  |
| TaDUF966-13A | CAAT-box | | CAAT | | 1287 | | 1291 | | 4 | |  |
| TaDUF966-13A | CAAT-box | | CAAT | | 1371 | | 1375 | | 4 | |  |
| TaDUF966-13A | CAAT-box | | CAAT | | 1405 | | 1409 | | 4 | |  |
| TaDUF966-13A | CAAT-box | | CAAT | | 1426 | | 1430 | | 4 | |  |
| TaDUF966-13A | CAAT-box | | CAAT | | 1471 | | 1475 | | 4 | |  |
| TaDUF966-13A | CAAT-box | | CCAAT | | 1473 | | 1478 | | 5 | |  |
| TaDUF966-13A | CAT-box | | GCCACT | | 1270 | | 1276 | | 6 | |  |
| TaDUF966-13A | CGTCA-motif | | CGTCA | | 174 | | 179 | | 5 | |  |
| TaDUF966-13A | GATA-motif | | AAGATAAGATT | | 441 | | 451 | | 10 | |  |
| TaDUF966-13A | GATA-motif | | GATAGGA | | 443 | | 450 | | 7 | |  |
| TaDUF966-13A | GATA-motif | | GATAGGG | | 955 | | 962 | | 7 | |  |
| TaDUF966-13A | G-box | | CACGAC | | 1315 | | 1321 | | 6 | |  |
| TaDUF966-13A | GT1-motif | | GGTTAA | | 1236 | | 1242 | | 6 | |  |
| TaDUF966-13A | MBS | | CAACTG | | 247 | | 253 | | 6 | |  |
| TaDUF966-13A | MBS | | CAACTG | | 580 | | 586 | | 6 | |  |
| TaDUF966-13A | MRE | | AACCTAA | | 41 | | 48 | | 7 | |  |
| TaDUF966-13A | P-box | | CCTTTTG | | 503 | | 510 | | 7 | |  |
| TaDUF966-13A | TATA-box | | ATATAA | | 186 | | 192 | | 6 | |  |
| TaDUF966-13A | TATA-box | | TATA | | 187 | | 191 | | 4 | |  |
| TaDUF966-13A | TATA-box | | TATA | | 225 | | 229 | | 4 | |  |
| TaDUF966-13A | TATA-box | | TATACA | | 346 | | 352 | | 6 | |  |
| TaDUF966-13A | TATA-box | | TATA | | 348 | | 352 | | 4 | |  |
| TaDUF966-13A | TATA-box | | TATA | | 390 | | 394 | | 4 | |  |
| TaDUF966-13A | TATA-box | | TATACA | | 398 | | 404 | | 6 | |  |
| TaDUF966-13A | TATA-box | | TATA | | 400 | | 404 | | 4 | |  |
| TaDUF966-13A | TATA-box | | TATACA | | 408 | | 414 | | 6 | |  |
| TaDUF966-13A | TATA-box | | TATA | | 410 | | 414 | | 4 | |  |
| TaDUF966-13A | TATA-box | | ATTATA | | 449 | | 455 | | 6 | |  |
| TaDUF966-13A | TATA-box | | TATATAA | | 450 | | 457 | | 7 | |  |
| TaDUF966-13A | TATA-box | | TATATA | | 451 | | 457 | | 6 | |  |
| TaDUF966-13A | TATA-box | | TATA | | 453 | | 457 | | 4 | |  |
| TaDUF966-13A | TATA-box | | TATAA | | 473 | | 478 | | 5 | |  |
| TaDUF966-13A | TATA-box | | TATA | | 474 | | 478 | | 4 | |  |
| TaDUF966-13A | TATA-box | | TACAAAA | | 490 | | 497 | | 7 | |  |
| TaDUF966-13A | TATA-box | | TATACA | | 526 | | 532 | | 6 | |  |
| TaDUF966-13A | TATA-box | | TATA | | 528 | | 532 | | 4 | |  |
| TaDUF966-13A | TATA-box | | ATATAA | | 593 | | 599 | | 6 | |  |
| TaDUF966-13A | TATA-box | | TATA | | 594 | | 598 | | 4 | |  |
| TaDUF966-13A | TATA-box | | TATA | | 600 | | 604 | | 4 | |  |
| TaDUF966-13A | TATA-box | | TATTTAAA | | 602 | | 610 | | 8 | |  |
| TaDUF966-13A | TATA-box | | TATACA | | 1138 | | 1144 | | 6 | |  |
| TaDUF966-13A | TATA-box | | TATATA | | 1140 | | 1146 | | 6 | |  |
| TaDUF966-13A | TATA-box | | TATA | | 1142 | | 1146 | | 4 | |  |
| TaDUF966-13A | TATA-box | | TATA | | 1187 | | 1191 | | 4 | |  |
| TaDUF966-13A | TATA-box | | TATA | | 1396 | | 1400 | | 4 | |  |
| TaDUF966-13A | TCT-motif | | TCTTAC | | 21 | | 27 | | 6 | |  |
| TaDUF966-13A | TGACG-motif | | TGACG | | 174 | | 179 | | 5 | |  |
| TaDUF966-13A | TGA-element | | AACGAC | | 1437 | | 1443 | | 6 | |  |
| TaDUF966-14A | A-box | | CCGTCC | | 531 | | 537 | | 6 | |  |
| TaDUF966-14A | ABRE | | ACGTG | | 401 | | 406 | | 5 | |  |
| TaDUF966-14A | ABRE | | ACGTG | | 590 | | 595 | | 5 | |  |
| TaDUF966-14A | ABRE | | ACGTG | | 643 | | 648 | | 5 | |  |
| TaDUF966-14A | ARE | | AAACCA | | 386 | | 392 | | 6 | |  |
| TaDUF966-14A | Box 4 | | ATTAAT | | 876 | | 882 | | 6 | |  |
| TaDUF966-14A | CAAT-box | | CAAT | | 73 | | 77 | | 4 | |  |
| TaDUF966-14A | CAAT-box | | CAAT | | 94 | | 98 | | 4 | |  |
| TaDUF966-14A | CAAT-box | | CAAAT | | 147 | | 152 | | 5 | |  |
| TaDUF966-14A | CAAT-box | | CAAT | | 214 | | 218 | | 4 | |  |
| TaDUF966-14A | CAAT-box | | CCAAT | | 511 | | 516 | | 5 | |  |
| TaDUF966-14A | CAAT-box | | CAAAT | | 539 | | 544 | | 5 | |  |
| TaDUF966-14A | CAAT-box | | CAAAT | | 583 | | 588 | | 5 | |  |
| TaDUF966-14A | CAAT-box | | CAAAT | | 681 | | 686 | | 5 | |  |
| TaDUF966-14A | CAAT-box | | CAAT | | 699 | | 703 | | 4 | |  |
| TaDUF966-14A | CAAT-box | | CAAT | | 742 | | 746 | | 4 | |  |
| TaDUF966-14A | CAAT-box | | CAAT | | 838 | | 842 | | 4 | |  |
| TaDUF966-14A | CAAT-box | | CAAT | | 874 | | 878 | | 4 | |  |
| TaDUF966-14A | CAAT-box | | CAAAT | | 990 | | 995 | | 5 | |  |
| TaDUF966-14A | CAAT-box | | CAAAT | | 1171 | | 1176 | | 5 | |  |
| TaDUF966-14A | CAAT-box | | CAAT | | 1208 | | 1212 | | 4 | |  |
| TaDUF966-14A | CAAT-box | | CAAT | | 1214 | | 1218 | | 4 | |  |
| TaDUF966-14A | CAAT-box | | CAAT | | 1301 | | 1305 | | 4 | |  |
| TaDUF966-14A | CAAT-box | | CAAAT | | 1308 | | 1313 | | 5 | |  |
| TaDUF966-14A | CAAT-box | | CAAT | | 1331 | | 1335 | | 4 | |  |
| TaDUF966-14A | CAAT-box | | CAAT | | 1377 | | 1381 | | 4 | |  |
| TaDUF966-14A | CAAT-box | | CAAT | | 1411 | | 1415 | | 4 | |  |
| TaDUF966-14A | CAT-box | | GCCACT | | 734 | | 740 | | 6 | |  |
| TaDUF966-14A | CCAAT-box | | CAACGG | | 181 | | 187 | | 6 | |  |
| TaDUF966-14A | CCAAT-box | | CAACGG | | 236 | | 242 | | 6 | |  |
| TaDUF966-14A | CGTCA-motif | | CGTCA | | 403 | | 408 | | 5 | |  |
| TaDUF966-14A | GATA-motif | | GATAGGA | | 884 | | 891 | | 7 | |  |
| TaDUF966-14A | G-box | | CACGTC | | 401 | | 407 | | 6 | |  |
| TaDUF966-14A | G-box | | CACGTC | | 589 | | 595 | | 6 | |  |
| TaDUF966-14A | G-box | | TACGTG | | 642 | | 648 | | 6 | |  |
| TaDUF966-14A | GT1-motif | | GGTTAA | | 286 | | 292 | | 6 | |  |
| TaDUF966-14A | LTR | | CCGAAA | | 612 | | 618 | | 6 | |  |
| TaDUF966-14A | MBS | | CAACTG | | 248 | | 254 | | 6 | |  |
| TaDUF966-14A | MBS | | CAACTG | | 436 | | 442 | | 6 | |  |
| TaDUF966-14A | MBS | | CAACTG | | 1368 | | 1374 | | 6 | |  |
| TaDUF966-14A | MBS | | CAACTG | | 1402 | | 1408 | | 6 | |  |
| TaDUF966-14A | TATA-box | | TATAAA | | 1 | | 7 | | 6 | |  |
| TaDUF966-14A | TATA-box | | TATAA | | 2 | | 7 | | 5 | |  |
| TaDUF966-14A | TATA-box | | TATA | | 3 | | 7 | | 4 | |  |
| TaDUF966-14A | TATA-box | | TATAAA | | 86 | | 92 | | 6 | |  |
| TaDUF966-14A | TATA-box | | TATAA | | 87 | | 92 | | 5 | |  |
| TaDUF966-14A | TATA-box | | TATA | | 88 | | 92 | | 4 | |  |
| TaDUF966-14A | TATA-box | | TACATAAA | | 393 | | 401 | | 8 | |  |
| TaDUF966-14A | TATA-box | | ATTATA | | 523 | | 529 | | 6 | |  |
| TaDUF966-14A | TATA-box | | TATAA | | 524 | | 529 | | 5 | |  |
| TaDUF966-14A | TATA-box | | TATA | | 525 | | 529 | | 4 | |  |
| TaDUF966-14A | TATA-box | | TATA | | 788 | | 792 | | 4 | |  |
| TaDUF966-14A | TATA-box | | TATACA | | 1013 | | 1019 | | 6 | |  |
| TaDUF966-14A | TATA-box | | TATA | | 1015 | | 1019 | | 4 | |  |
| TaDUF966-14A | TCA-element | | CCATCTTTTT | | 1322 | | 1331 | | 9 | |  |
| TaDUF966-14A | TGACG-motif | | TGACG | | 403 | | 408 | | 5 | |  |
| TaDUF966-14A | TGA-element | | AACGAC | | 1155 | | 1161 | | 6 | |  |
| TaDUF966-14B | ABRE | | ACGTG | | 88 | | 93 | | 5 | |  |
| TaDUF966-14B | ARE | | AAACCA | | 725 | | 731 | | 6 | |  |
| TaDUF966-14B | Box 4 | | ATTAAT | | 1354 | | 1360 | | 6 | |  |
| TaDUF966-14B | CAAT-box | | CAAT | | 94 | | 98 | | 4 | |  |
| TaDUF966-14B | CAAT-box | | CAAT | | 457 | | 461 | | 4 | |  |
| TaDUF966-14B | CAAT-box | | CAAT | | 475 | | 479 | | 4 | |  |
| TaDUF966-14B | CAAT-box | | CCAAT | | 582 | | 587 | | 5 | |  |
| TaDUF966-14B | CAAT-box | | CAAT | | 583 | | 587 | | 4 | |  |
| TaDUF966-14B | CAAT-box | | CAAT | | 665 | | 669 | | 4 | |  |
| TaDUF966-14B | CAAT-box | | CAAAT | | 674 | | 679 | | 5 | |  |
| TaDUF966-14B | CAAT-box | | CAAAT | | 684 | | 689 | | 5 | |  |
| TaDUF966-14B | CAAT-box | | CAAAT | | 702 | | 707 | | 5 | |  |
| TaDUF966-14B | CAAT-box | | CCAAT | | 713 | | 718 | | 5 | |  |
| TaDUF966-14B | CAAT-box | | CAAT | | 878 | | 882 | | 4 | |  |
| TaDUF966-14B | CAAT-box | | CAAT | | 1023 | | 1027 | | 4 | |  |
| TaDUF966-14B | CAAT-box | | CAAAT | | 1179 | | 1184 | | 5 | |  |
| TaDUF966-14B | CAAT-box | | CAAT | | 1361 | | 1365 | | 4 | |  |
| TaDUF966-14B | CAAT-box | | CAAT | | 1423 | | 1427 | | 4 | |  |
| TaDUF966-14B | CAAT-box | | CAAAT | | 1472 | | 1477 | | 5 | |  |
| TaDUF966-14B | CAAT-box | | CAAAT | | 1477 | | 1482 | | 5 | |  |
| TaDUF966-14B | CGTCA-motif | | CGTCA | | 604 | | 609 | | 5 | |  |
| TaDUF966-14B | CGTCA-motif | | CGTCA | | 1283 | | 1288 | | 5 | |  |
| TaDUF966-14B | G-Box | | CACGTT | | 88 | | 94 | | 6 | |  |
| TaDUF966-14B | G-box | | CACGAC | | 260 | | 266 | | 6 | |  |
| TaDUF966-14B | GT1-motif | | GGTTAA | | 12 | | 18 | | 6 | |  |
| TaDUF966-14B | MBS | | CAACTG | | 311 | | 317 | | 6 | |  |
| TaDUF966-14B | LTR | | CCGAAA | | 1277 | | 1283 | | 6 | |  |
| TaDUF966-14B | MRE | | AACCTAA | | 1111 | | 1118 | | 7 | |  |
| TaDUF966-14B | MRE | | AACCTAA | | 1142 | | 1149 | | 7 | |  |
| TaDUF966-14B | Sp1 | | GGGCGG | | 8 | | 14 | | 6 | |  |
| TaDUF966-14B | Sp1 | | GGGCGG | | 984 | | 990 | | 6 | |  |
| TaDUF966-14B | TATA-box | | TATA | | 321 | | 325 | | 4 | |  |
| TaDUF966-14B | TATA-box | | TATACA | | 401 | | 407 | | 6 | |  |
| TaDUF966-14B | TATA-box | | TATA | | 403 | | 407 | | 4 | |  |
| TaDUF966-14B | TATA-box | | TACAAAA | | 639 | | 646 | | 7 | |  |
| TaDUF966-14B | TATA-box | | TATA | | 862 | | 866 | | 4 | |  |
| TaDUF966-14B | TATA-box | | TATAA | | 890 | | 895 | | 5 | |  |
| TaDUF966-14B | TATA-box | | TATA | | 891 | | 895 | | 4 | |  |
| TaDUF966-14B | TATA-box | | TATA | | 1306 | | 1310 | | 4 | |  |
| TaDUF966-14B | TC-rich repeats | | GTTTTCTTAC | | 777 | | 786 | | 9 | |  |
| TaDUF966-14B | TGACG-motif | | TGACG | | 604 | | 609 | | 5 | |  |
| TaDUF966-14B | TGACG-motif | | TGACG | | 1283 | | 1288 | | 5 | |  |
| TaDUF966-14D | ABRE | | ACGTG | | 356 | | 361 | | 5 | |  |
| TaDUF966-14D | ABRE | | ACGTG | | 593 | | 598 | | 5 | |  |
| TaDUF966-14D | ABRE | | ACGTG | | 700 | | 705 | | 5 | |  |
| TaDUF966-14D | ABRE | | CGCACGTGTC | | 802 | | 811 | | 9 | |  |
| TaDUF966-14D | ABRE | | ACGTG | | 921 | | 926 | | 5 | |  |
| TaDUF966-14D | ARE | | AAACCA | | 253 | | 259 | | 6 | |  |
| TaDUF966-14D | ARE | | AAACCA | | 628 | | 634 | | 6 | |  |
| TaDUF966-14D | CAAT-box | | CAAT | | 87 | | 91 | | 4 | |  |
| TaDUF966-14D | CAAT-box | | CAAT | | 105 | | 109 | | 4 | |  |
| TaDUF966-14D | CAAT-box | | CCAAT | | 245 | | 250 | | 5 | |  |
| TaDUF966-14D | CAAT-box | | CAAT | | 390 | | 394 | | 4 | |  |
| TaDUF966-14D | CAAT-box | | CAAT | | 426 | | 430 | | 4 | |  |
| TaDUF966-14D | CAAT-box | | CAAT | | 560 | | 564 | | 4 | |  |
| TaDUF966-14D | CAAT-box | | CAAT | | 569 | | 573 | | 4 | |  |
| TaDUF966-14D | CAAT-box | | CAAT | | 635 | | 639 | | 4 | |  |
| TaDUF966-14D | CAAT-box | | CAAT | | 778 | | 782 | | 4 | |  |
| TaDUF966-14D | CAAT-box | | CAAT | | 834 | | 838 | | 4 | |  |
| TaDUF966-14D | CAAT-box | | CAAT | | 839 | | 843 | | 4 | |  |
| TaDUF966-14D | CAAT-box | | CCAAT | | 845 | | 850 | | 5 | |  |
| TaDUF966-14D | CAAT-box | | CAAT | | 1019 | | 1023 | | 4 | |  |
| TaDUF966-14D | CAAT-box | | CAAT | | 1140 | | 1144 | | 4 | |  |
| TaDUF966-14D | CAAT-box | | CAAAT | | 1154 | | 1159 | | 5 | |  |
| TaDUF966-14D | CAAT-box | | CAAAT | | 1180 | | 1185 | | 5 | |  |
| TaDUF966-14D | CAAT-box | | CAAT | | 1257 | | 1261 | | 4 | |  |
| TaDUF966-14D | CAAT-box | | CAAT | | 1325 | | 1329 | | 4 | |  |
| TaDUF966-14D | CAAT-box | | CAAAT | | 1392 | | 1397 | | 5 | |  |
| TaDUF966-14D | CAAT-box | | CAAT | | 1429 | | 1433 | | 4 | |  |
| TaDUF966-14D | CAAT-box | | CAAT | | 1435 | | 1439 | | 4 | |  |
| TaDUF966-14D | G-Box | | CACGTT | | 356 | | 362 | | 6 | |  |
| TaDUF966-14D | G-Box | | CACGTT | | 593 | | 599 | | 6 | |  |
| TaDUF966-14D | G-box | | CACGTC | | 699 | | 705 | | 6 | |  |
| TaDUF966-14D | G-box | | CACGTC | | 920 | | 926 | | 6 | |  |
| TaDUF966-14D | G-box | | CACGAC | | 955 | | 961 | | 6 | |  |
| TaDUF966-14D | MBS | | CAACTG | | 220 | | 226 | | 6 | |  |
| TaDUF966-14D | O2-site | | GATGATGTGG | | 1235 | | 1245 | | 10 | |  |
| TaDUF966-14D | TATA-box | | ATTATA | | 240 | | 246 | | 6 | |  |
| TaDUF966-14D | TATA-box | | TATAA | | 241 | | 246 | | 5 | |  |
| TaDUF966-14D | TATA-box | | TATA | | 242 | | 246 | | 4 | |  |
| TaDUF966-14D | TATA-box | | TATA | | 307 | | 311 | | 4 | |  |
| TaDUF966-14D | TATA-box | | TATATA | | 337 | | 343 | | 6 | |  |
| TaDUF966-14D | TATA-box | | ATATAT | | 338 | | 344 | | 6 | |  |
| TaDUF966-14D | TATA-box | | TATATA | | 339 | | 345 | | 6 | |  |
| TaDUF966-14D | TATA-box | | ATATAT | | 340 | | 346 | | 6 | |  |
| TaDUF966-14D | TATA-box | | TATA | | 341 | | 345 | | 4 | |  |
| TaDUF966-14D | TCT-motif | | TCTTAC | | 662 | | 668 | | 6 | |  |
| TaDUF966-14D | TC-rich repeats | | ATTCTCTAAC | | 1380 | | 1389 | | 9 | |  |

Continue to the previous **Supplementary Table 10.**

| **Gene** | **Orientation** | **Aligned species** | **Function** |
| --- | --- | --- | --- |
| TaDUF966-1A | - | *Arabidopsis thaliana* | cis-acting element involved in the abscisic acid responsiveness |
| TaDUF966-1A | - | *Arabidopsis thaliana* | part of a module for light response |
| TaDUF966-1A | - | *Zea mays* | cis-acting regulatory element essential for the anaerobic induction |
| TaDUF966-1A | + | *Zea mays* | cis-acting regulatory element essential for the anaerobic induction |
| TaDUF966-1A | - | *Nicotiana glutinosa* |  |
| TaDUF966-1A | - | *Nicotiana glutinosa* |  |
| TaDUF966-1A | + | *Arabidopsis thaliana* | common cis-acting element in promoter and enhancer regions |
| TaDUF966-1A | + | *Nicotiana glutinosa* |  |
| TaDUF966-1A | - | *Arabidopsis thaliana* | common cis-acting element in promoter and enhancer regions |
| TaDUF966-1A | + | *Nicotiana glutinosa* |  |
| TaDUF966-1A | - | *Arabidopsis thaliana* | common cis-acting element in promoter and enhancer regions |
| TaDUF966-1A | + | *Pisum sativum* | common cis-acting element in promoter and enhancer regions |
| TaDUF966-1A | + | *Pisum sativum* | common cis-acting element in promoter and enhancer regions |
| TaDUF966-1A | - | *Nicotiana glutinosa* |  |
| TaDUF966-1A | - | *Nicotiana glutinosa* |  |
| TaDUF966-1A | - | *Arabidopsis thaliana* | common cis-acting element in promoter and enhancer regions |
| TaDUF966-1A | - | *Pisum sativum* | common cis-acting element in promoter and enhancer regions |
| TaDUF966-1A | + | *Pisum sativum* | common cis-acting element in promoter and enhancer regions |
| TaDUF966-1A | - | *Pisum sativum* | common cis-acting element in promoter and enhancer regions |
| TaDUF966-1A | + | *Nicotiana glutinosa* |  |
| TaDUF966-1A | + | *Arabidopsis thaliana* | common cis-acting element in promoter and enhancer regions |
| TaDUF966-1A | + | *Nicotiana glutinosa* |  |
| TaDUF966-1A | + | *Pisum sativum* | common cis-acting element in promoter and enhancer regions |
| TaDUF966-1A | + | *Nicotiana glutinosa* |  |
| TaDUF966-1A | + | *Nicotiana glutinosa* |  |
| TaDUF966-1A | + | *Pisum sativum* | common cis-acting element in promoter and enhancer regions |
| TaDUF966-1A | + | *Arabidopsis thaliana* | common cis-acting element in promoter and enhancer regions |
| TaDUF966-1A | + | *Nicotiana glutinosa* |  |
| TaDUF966-1A | + | *Arabidopsis thaliana* | cis-acting regulatory element related to meristem expression |
| TaDUF966-1A | + | *Hordeum vulgare* | cis-acting regulatory element involved in the MeJA-responsiveness |
| TaDUF966-1A | + | *Zea mays* | cis-acting regulatory element involved in light responsiveness |
| TaDUF966-1A | - | *Hordeum vulgare* | cis-acting element involved in low-temperature responsiveness |
| TaDUF966-1A | + | *Arabidopsis thaliana* | MYB binding site involved in drought-inducibility |
| TaDUF966-1A | + | *Arabidopsis thaliana* | MYB binding site involved in drought-inducibility |
| TaDUF966-1A | - | *Zea mays* | core promoter element around -30 of transcription start |
| TaDUF966-1A | - | *Arabidopsis thaliana* | core promoter element around -30 of transcription start |
| TaDUF966-1A | + | *Arabidopsis thaliana* | core promoter element around -30 of transcription start |
| TaDUF966-1A | - | *Helianthus annuus* | core promoter element around -30 of transcription start |
| TaDUF966-1A | - | *Arabidopsis thaliana* | core promoter element around -30 of transcription start |
| TaDUF966-1A | + | *Arabidopsis thaliana* | core promoter element around -30 of transcription start |
| TaDUF966-1A | - | *Arabidopsis thaliana* | core promoter element around -30 of transcription start |
| TaDUF966-1A | - | *Pisum sativum* | core promoter element around -30 of transcription start |
| TaDUF966-1A | - | *Helianthus annuus* | core promoter element around -30 of transcription start |
| TaDUF966-1A | - | *Arabidopsis thaliana* | core promoter element around -30 of transcription start |
| TaDUF966-1A | - | *Arabidopsis thaliana* | core promoter element around -30 of transcription start |
| TaDUF966-1A | - | *Brassica oleracea* | cis-acting element involved in salicylic acid responsiveness |
| TaDUF966-1A | + | *Arabidopsis thaliana* | part of a light responsive element |
| TaDUF966-1A | - | *Hordeum vulgare* | cis-acting regulatory element involved in the MeJA-responsiveness |
| TaDUF966-1A | + | *Brassica oleracea* | auxin-responsive element |
| TaDUF966-2B | - | *Petroselinum crispum* | cis-acting regulatory element |
| TaDUF966-2B | + | *Petroselinum crispum* | cis-acting regulatory element |
| TaDUF966-2B | - | *Petroselinum crispum* | cis-acting regulatory element |
| TaDUF966-2B | + | *Oryza sativa* | cis-acting element involved in the abscisic acid responsiveness |
| TaDUF966-2B | - | *Pisum sativum* | common cis-acting element in promoter and enhancer regions |
| TaDUF966-2B | + | *Nicotiana glutinosa* |  |
| TaDUF966-2B | - | *Nicotiana glutinosa* |  |
| TaDUF966-2B | + | *Nicotiana glutinosa* |  |
| TaDUF966-2B | - | *Pisum sativum* | common cis-acting element in promoter and enhancer regions |
| TaDUF966-2B | + | *Pisum sativum* | common cis-acting element in promoter and enhancer regions |
| TaDUF966-2B | - | *Pisum sativum* | common cis-acting element in promoter and enhancer regions |
| TaDUF966-2B | - | *Nicotiana glutinosa* |  |
| TaDUF966-2B | - | *Pisum sativum* | common cis-acting element in promoter and enhancer regions |
| TaDUF966-2B | - | *Pisum sativum* | common cis-acting element in promoter and enhancer regions |
| TaDUF966-2B | - | *Nicotiana glutinosa* |  |
| TaDUF966-2B | - | *Nicotiana glutinosa* |  |
| TaDUF966-2B | + | *Arabidopsis thaliana* | cis-acting regulatory element related to meristem expression |
| TaDUF966-2B | + | *Arabidopsis thaliana* | cis-acting regulatory element related to meristem expression |
| TaDUF966-2B | - | *Arabidopsis thaliana* | cis-acting regulatory element related to meristem expression |
| TaDUF966-2B | - | *Hordeum vulgare* | MYBHv1 binding site |
| TaDUF966-2B | + | *Hordeum vulgare* | cis-acting regulatory element involved in the MeJA-responsiveness |
| TaDUF966-2B | - | *Hordeum vulgare* | cis-acting regulatory element involved in the MeJA-responsiveness |
| TaDUF966-2B | - | *Hordeum vulgare* | cis-acting regulatory element involved in the MeJA-responsiveness |
| TaDUF966-2B | - | *Hordeum vulgare* | cis-acting regulatory element involved in the MeJA-responsiveness |
| TaDUF966-2B | + | *Brassica oleracea* | cis-acting regulatory element involved in light responsiveness |
| TaDUF966-2B | + | *Zea mays* | cis-acting regulatory element involved in light responsiveness |
| TaDUF966-2B | + | *Zea mays* | enhancer-like element involved in anoxic specific inducibility |
| TaDUF966-2B | - | *Zea mays* | enhancer-like element involved in anoxic specific inducibility |
| TaDUF966-2B | - | *Zea mays* | enhancer-like element involved in anoxic specific inducibility |
| TaDUF966-2B | - | *Arabidopsis thaliana* | MYB binding site involved in drought-inducibility |
| TaDUF966-2B | - | *Oryza sativa* | light responsive element |
| TaDUF966-2B | - | *Oryza sativa* | light responsive element |
| TaDUF966-2B | - | *Oryza sativa* | light responsive element |
| TaDUF966-2B | - | *Oryza sativa* | light responsive element |
| TaDUF966-2B | + | *Oryza sativa* | light responsive element |
| TaDUF966-2B | - | *Arabidopsis thaliana* | core promoter element around -30 of transcription start |
| TaDUF966-2B | + | *Brassica oleracea* | core promoter element around -30 of transcription start |
| TaDUF966-2B | - | *Arabidopsis thaliana* | core promoter element around -30 of transcription start |
| TaDUF966-2B | - | *Helianthus annuus* | core promoter element around -30 of transcription start |
| TaDUF966-2B | - | *Arabidopsis thaliana* | core promoter element around -30 of transcription start |
| TaDUF966-2B | - | *Hordeum vulgare* | cis-acting regulatory element involved in the MeJA-responsiveness |
| TaDUF966-2B | + | *Hordeum vulgare* | cis-acting regulatory element involved in the MeJA-responsiveness |
| TaDUF966-2B | + | *Hordeum vulgare* | cis-acting regulatory element involved in the MeJA-responsiveness |
| TaDUF966-2B | + | *Hordeum vulgare* | cis-acting regulatory element involved in the MeJA-responsiveness |
| TaDUF966-3A | + | *Arabidopsis thaliana* | cis-acting element involved in the abscisic acid responsiveness |
| TaDUF966-3A | + | *Arabidopsis thaliana* | cis-acting element involved in the abscisic acid responsiveness |
| TaDUF966-3A | + | *Arabidopsis thaliana* | cis-acting element involved in the abscisic acid responsiveness |
| TaDUF966-3A | + | *Arabidopsis thaliana* | cis-acting element involved in the abscisic acid responsiveness |
| TaDUF966-3A | + | *Arabidopsis thaliana* | cis-acting element involved in the abscisic acid responsiveness |
| TaDUF966-3A | + | *Arabidopsis thaliana* | part of a module for light response |
| TaDUF966-3A | + | *Zea mays* | cis-acting regulatory element essential for the anaerobic induction |
| TaDUF966-3A | + | *Zea mays* | cis-acting regulatory element essential for the anaerobic induction |
| TaDUF966-3A | + | *Petroselinum crispum* | part of a conserved DNA module involved in light responsiveness |
| TaDUF966-3A | + | *Petroselinum crispum* | part of a conserved DNA module involved in light responsiveness |
| TaDUF966-3A | - | *Pisum sativum* | common cis-acting element in promoter and enhancer regions |
| TaDUF966-3A | - | *Pisum sativum* | common cis-acting element in promoter and enhancer regions |
| TaDUF966-3A | - | *Nicotiana glutinosa* |  |
| TaDUF966-3A | - | *Nicotiana glutinosa* |  |
| TaDUF966-3A | - | *Pisum sativum* | common cis-acting element in promoter and enhancer regions |
| TaDUF966-3A | - | *Pisum sativum* | common cis-acting element in promoter and enhancer regions |
| TaDUF966-3A | + | *Nicotiana glutinosa* |  |
| TaDUF966-3A | - | *Nicotiana glutinosa* |  |
| TaDUF966-3A | - | *Nicotiana glutinosa* |  |
| TaDUF966-3A | - | *Nicotiana glutinosa* |  |
| TaDUF966-3A | - | *Pisum sativum* | common cis-acting element in promoter and enhancer regions |
| TaDUF966-3A | - | *Pisum sativum* | common cis-acting element in promoter and enhancer regions |
| TaDUF966-3A | - | *Nicotiana glutinosa* |  |
| TaDUF966-3A | - | *Arabidopsis thaliana* | cis-acting regulatory element related to meristem expression |
| TaDUF966-3A | + | *Brassica oleracea* | cis-acting regulatory element involved in light responsiveness |
| TaDUF966-3A | + | *Arabidopsis thaliana* | cis-acting regulatory element involved in light responsiveness |
| TaDUF966-3A | - | *Pisum sativum* | cis-acting regulatory element involved in light responsiveness |
| TaDUF966-3A | - | *Pisum sativum* | cis-acting regulatory element involved in light responsiveness |
| TaDUF966-3A | - | *Pisum sativum* | cis-acting regulatory element involved in light responsiveness |
| TaDUF966-3A | - | *Pisum sativum* | cis-acting regulatory element involved in light responsiveness |
| TaDUF966-3A | + | *Pisum sativum* | cis-acting regulatory element involved in light responsiveness |
| TaDUF966-3A | + | *Hordeum vulgare* | cis-acting element involved in low-temperature responsiveness |
| TaDUF966-3A | - | *Oryza sativa* | light responsive element |
| TaDUF966-3A | - | *Helianthus annuus* | core promoter element around -30 of transcription start |
| TaDUF966-3A | + | *Arabidopsis thaliana* | core promoter element around -30 of transcription start |
| TaDUF966-3A | - | *Helianthus annuus* | core promoter element around -30 of transcription start |
| TaDUF966-3A | + | *Arabidopsis thaliana* | core promoter element around -30 of transcription start |
| TaDUF966-3A | - | *Helianthus annuus* | core promoter element around -30 of transcription start |
| TaDUF966-3A | + | *Arabidopsis thaliana* | core promoter element around -30 of transcription start |
| TaDUF966-3A | - | *Helianthus annuus* | core promoter element around -30 of transcription start |
| TaDUF966-3A | + | *Arabidopsis thaliana* | core promoter element around -30 of transcription start |
| TaDUF966-3A | + | *Arabidopsis thaliana* | core promoter element around -30 of transcription start |
| TaDUF966-3A | + | *Arabidopsis thaliana* | core promoter element around -30 of transcription start |
| TaDUF966-3A | + | *Arabidopsis thaliana* | core promoter element around -30 of transcription start |
| TaDUF966-3A | + | *Brassica napus* | core promoter element around -30 of transcription start |
| TaDUF966-3A | + | *Arabidopsis thaliana* | core promoter element around -30 of transcription start |
| TaDUF966-3A | + | *Brassica oleracea* | core promoter element around -30 of transcription start |
| TaDUF966-3A | + | *Arabidopsis thaliana* | core promoter element around -30 of transcription start |
| TaDUF966-3A | - | *Helianthus annuus* | core promoter element around -30 of transcription start |
| TaDUF966-3A | + | *Arabidopsis thaliana* | core promoter element around -30 of transcription start |
| TaDUF966-3A | + | *Arabidopsis thaliana* | core promoter element around -30 of transcription start |
| TaDUF966-3A | - | *Helianthus annuus* | core promoter element around -30 of transcription start |
| TaDUF966-3A | + | *Arabidopsis thaliana* | core promoter element around -30 of transcription start |
| TaDUF966-3A | - | *Helianthus annuus* | core promoter element around -30 of transcription start |
| TaDUF966-3A | + | *Arabidopsis thaliana* | core promoter element around -30 of transcription start |
| TaDUF966-3A | + | *Brassica napus* | core promoter element around -30 of transcription start |
| TaDUF966-3A | + | *Arabidopsis thaliana* | core promoter element around -30 of transcription start |
| TaDUF966-3A | + | *Arabidopsis thaliana* | core promoter element around -30 of transcription start |
| TaDUF966-3A | - | *Helianthus annuus* | core promoter element around -30 of transcription start |
| TaDUF966-3A | + | *Arabidopsis thaliana* | core promoter element around -30 of transcription start |
| TaDUF966-3A | - | *Arabidopsis thaliana* | core promoter element around -30 of transcription start |
| TaDUF966-3A | - | *Brassica napus* | core promoter element around -30 of transcription start |
| TaDUF966-3A | - | *Arabidopsis thaliana* | core promoter element around -30 of transcription start |
| TaDUF966-3A | - | *Arabidopsis thaliana* | core promoter element around -30 of transcription start |
| TaDUF966-3A | - | *Arabidopsis thaliana* | core promoter element around -30 of transcription start |
| TaDUF966-3A | + | *Brassica oleracea* | core promoter element around -30 of transcription start |
| TaDUF966-3A | - | *Arabidopsis thaliana* | core promoter element around -30 of transcription start |
| TaDUF966-3B | + | *Petroselinum crispum* | cis-acting regulatory element |
| TaDUF966-3B | - | *Petroselinum crispum* | cis-acting regulatory element |
| TaDUF966-3B | - | *Zea mays* | cis-acting regulatory element essential for the anaerobic induction |
| TaDUF966-3B | + | *Zea mays* | cis-acting regulatory element essential for the anaerobic induction |
| TaDUF966-3B | + | *Zea mays* | cis-acting regulatory element essential for the anaerobic induction |
| TaDUF966-3B | + | *Nicotiana glutinosa* |  |
| TaDUF966-3B | + | *Nicotiana glutinosa* |  |
| TaDUF966-3B | + | *Nicotiana glutinosa* |  |
| TaDUF966-3B | + | *Arabidopsis thaliana* | common cis-acting element in promoter and enhancer regions |
| TaDUF966-3B | + | *Nicotiana glutinosa* |  |
| TaDUF966-3B | + | *Pisum sativum* | common cis-acting element in promoter and enhancer regions |
| TaDUF966-3B | - | *Pisum sativum* | common cis-acting element in promoter and enhancer regions |
| TaDUF966-3B | - | *Pisum sativum* | common cis-acting element in promoter and enhancer regions |
| TaDUF966-3B | - | *Nicotiana glutinosa* |  |
| TaDUF966-3B | - | *Arabidopsis thaliana* | common cis-acting element in promoter and enhancer regions |
| TaDUF966-3B | + | *Pisum sativum* | common cis-acting element in promoter and enhancer regions |
| TaDUF966-3B | + | *Nicotiana glutinosa* |  |
| TaDUF966-3B | - | *Pisum sativum* | common cis-acting element in promoter and enhancer regions |
| TaDUF966-3B | + | *Nicotiana glutinosa* |  |
| TaDUF966-3B | - | *Arabidopsis thaliana* | common cis-acting element in promoter and enhancer regions |
| TaDUF966-3B | - | *Nicotiana glutinosa* |  |
| TaDUF966-3B | - | *Nicotiana glutinosa* |  |
| TaDUF966-3B | - | *Nicotiana glutinosa* |  |
| TaDUF966-3B | + | *Nicotiana glutinosa* |  |
| TaDUF966-3B | - | *Nicotiana glutinosa* |  |
| TaDUF966-3B | + | *Hordeum vulgare* | cis-acting regulatory element involved in the MeJA-responsiveness |
| TaDUF966-3B | + | *Arabidopsis thaliana* | part of a light responsive element |
| TaDUF966-3B | + | *Triticum aestivum* | cis-acting regulatory element involved in light responsiveness |
| TaDUF966-3B | - | *Arabidopsis thaliana* | cis-acting regulatory element involved in light responsiveness |
| TaDUF966-3B | + | *Avena sativa* | light responsive element |
| TaDUF966-3B | + | *Larix laricina* | part of a light responsive element |
| TaDUF966-3B | - | *Hordeum vulgare* | cis-acting element involved in low-temperature responsiveness |
| TaDUF966-3B | + | *Hordeum vulgare* | cis-acting element involved in low-temperature responsiveness |
| TaDUF966-3B | + | *Hordeum vulgare* | cis-acting element involved in low-temperature responsiveness |
| TaDUF966-3B | + | *Arabidopsis thaliana* | MYB binding site involved in drought-inducibility |
| TaDUF966-3B | + | *Arabidopsis thaliana* | MYB binding site involved in drought-inducibility |
| TaDUF966-3B | - | *Daucus carota* | core promoter element around -30 of transcription start |
| TaDUF966-3B | - | *Brassica juncea* | core promoter element around -30 of transcription start |
| TaDUF966-3B | - | *Helianthus annuus* | core promoter element around -30 of transcription start |
| TaDUF966-3B | - | *Arabidopsis thaliana* | core promoter element around -30 of transcription start |
| TaDUF966-3B | + | *Arabidopsis thaliana* | core promoter element around -30 of transcription start |
| TaDUF966-3B | - | *Helianthus annuus* | core promoter element around -30 of transcription start |
| TaDUF966-3B | - | *Arabidopsis thaliana* | core promoter element around -30 of transcription start |
| TaDUF966-3B | + | *Arabidopsis thaliana* | core promoter element around -30 of transcription start |
| TaDUF966-3B | - | *Arabidopsis thaliana* | core promoter element around -30 of transcription start |
| TaDUF966-3B | + | *Arabidopsis thaliana* | core promoter element around -30 of transcription start |
| TaDUF966-3B | + | *Brassica oleracea* | core promoter element around -30 of transcription start |
| TaDUF966-3B | + | *Arabidopsis thaliana* | core promoter element around -30 of transcription start |
| TaDUF966-3B | - | *Arabidopsis thaliana* | core promoter element around -30 of transcription start |
| TaDUF966-3B | - | *Arabidopsis thaliana* | core promoter element around -30 of transcription start |
| TaDUF966-3B | + | *Brassica napus* | core promoter element around -30 of transcription start |
| TaDUF966-3B | - | *Arabidopsis thaliana* | core promoter element around -30 of transcription start |
| TaDUF966-3B | - | *Arabidopsis thaliana* | core promoter element around -30 of transcription start |
| TaDUF966-3B | + | *Brassica oleracea* | core promoter element around -30 of transcription start |
| TaDUF966-3B | - | *Arabidopsis thaliana* | core promoter element around -30 of transcription start |
| TaDUF966-3B | - | *Arabidopsis thaliana* | part of a light responsive element |
| TaDUF966-3B | - | *Hordeum vulgare* | cis-acting regulatory element involved in the MeJA-responsiveness |
| TaDUF966-3B | - | *Brassica oleracea* | auxin-responsive element |
| TaDUF966-3B | + | *Brassica oleracea* | auxin-responsive element |
| TaDUF966-3B | + | *Brassica oleracea* | auxin-responsive element |
| TaDUF966-3D | + | *Arabidopsis thaliana* | cis-acting element involved in the abscisic acid responsiveness |
| TaDUF966-3D | + | *Arabidopsis thaliana* | cis-acting element involved in the abscisic acid responsiveness |
| TaDUF966-3D | + | *Arabidopsis thaliana* | cis-acting element involved in the abscisic acid responsiveness |
| TaDUF966-3D | - | *Arabidopsis thaliana* | part of a module for light response |
| TaDUF966-3D | - | *Zea mays* | cis-acting regulatory element essential for the anaerobic induction |
| TaDUF966-3D | + | *Zea mays* | cis-acting regulatory element essential for the anaerobic induction |
| TaDUF966-3D | - | *Zea mays* | cis-acting regulatory element essential for the anaerobic induction |
| TaDUF966-3D | + | *Pisum sativum* | common cis-acting element in promoter and enhancer regions |
| TaDUF966-3D | - | *Pisum sativum* | common cis-acting element in promoter and enhancer regions |
| TaDUF966-3D | - | *Pisum sativum* | common cis-acting element in promoter and enhancer regions |
| TaDUF966-3D | - | *Nicotiana glutinosa* |  |
| TaDUF966-3D | - | *Arabidopsis thaliana* | common cis-acting element in promoter and enhancer regions |
| TaDUF966-3D | - | *Nicotiana glutinosa* |  |
| TaDUF966-3D | - | *Nicotiana glutinosa* |  |
| TaDUF966-3D | - | *Nicotiana glutinosa* |  |
| TaDUF966-3D | - | *Pisum sativum* | common cis-acting element in promoter and enhancer regions |
| TaDUF966-3D | - | *Nicotiana glutinosa* |  |
| TaDUF966-3D | - | *Pisum sativum* | common cis-acting element in promoter and enhancer regions |
| TaDUF966-3D | - | *Nicotiana glutinosa* |  |
| TaDUF966-3D | + | *Nicotiana glutinosa* |  |
| TaDUF966-3D | + | *Nicotiana glutinosa* |  |
| TaDUF966-3D | + | *Pisum sativum* | common cis-acting element in promoter and enhancer regions |
| TaDUF966-3D | - | *Nicotiana glutinosa* |  |
| TaDUF966-3D | + | *Nicotiana glutinosa* |  |
| TaDUF966-3D | - | *Petunia hybrida* | common cis-acting element in promoter and enhancer regions |
| TaDUF966-3D | + | *Nicotiana glutinosa* |  |
| TaDUF966-3D | - | *Nicotiana glutinosa* |  |
| TaDUF966-3D | - | *Pisum sativum* | common cis-acting element in promoter and enhancer regions |
| TaDUF966-3D | - | *Pisum sativum* | common cis-acting element in promoter and enhancer regions |
| TaDUF966-3D | - | *Hordeum vulgare* | MYBHv1 binding site |
| TaDUF966-3D | - | *Hordeum vulgare* | cis-acting regulatory element involved in the MeJA-responsiveness |
| TaDUF966-3D | + | *Arabidopsis thaliana* | part of a light responsive element |
| TaDUF966-3D | - | *Solanum tuberosum* | part of a light responsive element |
| TaDUF966-3D | + | *Pisum sativum* | cis-acting regulatory element involved in light responsiveness |
| TaDUF966-3D | + | *Arabidopsis thaliana* | cis-acting regulatory element involved in light responsiveness |
| TaDUF966-3D | + | *Arabidopsis thaliana* | cis-acting regulatory element involved in light responsiveness |
| TaDUF966-3D | + | *Zea mays* | cis-acting regulatory element involved in light responsiveness |
| TaDUF966-3D | + | *Zea mays* | enhancer-like element involved in anoxic specific inducibility |
| TaDUF966-3D | + | *Arabidopsis thaliana* | MYB binding site involved in drought-inducibility |
| TaDUF966-3D | + | *Petroselinum crispum* | MYB binding site involved in light responsiveness |
| TaDUF966-3D | + | *Avena sativa* | core promoter element around -30 of transcription start |
| TaDUF966-3D | - | *Daucus carota* | core promoter element around -30 of transcription start |
| TaDUF966-3D | - | *Brassica juncea* | core promoter element around -30 of transcription start |
| TaDUF966-3D | - | *Helianthus annuus* | core promoter element around -30 of transcription start |
| TaDUF966-3D | - | *Arabidopsis thaliana* | core promoter element around -30 of transcription start |
| TaDUF966-3D | + | *Arabidopsis thaliana* | core promoter element around -30 of transcription start |
| TaDUF966-3D | + | *Brassica napus* | core promoter element around -30 of transcription start |
| TaDUF966-3D | + | *Arabidopsis thaliana* | core promoter element around -30 of transcription start |
| TaDUF966-3D | - | *Arabidopsis thaliana* | core promoter element around -30 of transcription start |
| TaDUF966-3D | - | *Brassica napus* | core promoter element around -30 of transcription start |
| TaDUF966-3D | - | *Arabidopsis thaliana* | core promoter element around -30 of transcription start |
| TaDUF966-3D | - | *Brassica napus* | core promoter element around -30 of transcription start |
| TaDUF966-3D | - | *Arabidopsis thaliana* | core promoter element around -30 of transcription start |
| TaDUF966-3D | + | *Brassica oleracea* | core promoter element around -30 of transcription start |
| TaDUF966-3D | - | *Arabidopsis thaliana* | core promoter element around -30 of transcription start |
| TaDUF966-3D | + | *Brassica oleracea* | core promoter element around -30 of transcription start |
| TaDUF966-3D | - | *Arabidopsis thaliana* | core promoter element around -30 of transcription start |
| TaDUF966-3D | + | *Nicotiana tabacum* | cis-acting element involved in defense and stress responsiveness |
| TaDUF966-3D | + | *Arabidopsis thaliana* | part of a light responsive element |
| TaDUF966-3D | + | *Hordeum vulgare* | cis-acting regulatory element involved in the MeJA-responsiveness |
| TaDUF966-3D | + | *Brassica oleracea* | auxin-responsive element |
| TaDUF966-4A | + | *Triticum aestivum* | cis-acting element involved in the abscisic acid responsiveness |
| TaDUF966-4A | + | *Arabidopsis thaliana* | cis-acting element involved in the abscisic acid responsiveness |
| TaDUF966-4A | - | *Arabidopsis thaliana* | part of a module for light response |
| TaDUF966-4A | - | *Arabidopsis thaliana* | part of a module for light response |
| TaDUF966-4A | - | *Zea mays* | cis-acting regulatory element essential for the anaerobic induction |
| TaDUF966-4A | - | *Nicotiana glutinosa* |  |
| TaDUF966-4A | + | *Arabidopsis thaliana* | common cis-acting element in promoter and enhancer regions |
| TaDUF966-4A | + | *Nicotiana glutinosa* |  |
| TaDUF966-4A | - | *Pisum sativum* | common cis-acting element in promoter and enhancer regions |
| TaDUF966-4A | + | *Arabidopsis thaliana* | common cis-acting element in promoter and enhancer regions |
| TaDUF966-4A | + | *Nicotiana glutinosa* |  |
| TaDUF966-4A | - | *Pisum sativum* | common cis-acting element in promoter and enhancer regions |
| TaDUF966-4A | + | *Nicotiana glutinosa* |  |
| TaDUF966-4A | - | *Pisum sativum* | common cis-acting element in promoter and enhancer regions |
| TaDUF966-4A | - | *Nicotiana glutinosa* |  |
| TaDUF966-4A | - | *Nicotiana glutinosa* |  |
| TaDUF966-4A | - | *Pisum sativum* | common cis-acting element in promoter and enhancer regions |
| TaDUF966-4A | + | *Nicotiana glutinosa* |  |
| TaDUF966-4A | - | *Arabidopsis thaliana* | cis-acting regulatory element related to meristem expression |
| TaDUF966-4A | + | *Hordeum vulgare* | cis-acting regulatory element involved in the MeJA-responsiveness |
| TaDUF966-4A | - | *Zea mays* | cis-acting regulatory element involved in light responsiveness |
| TaDUF966-4A | + | *Zea mays* | cis-acting regulatory element involved in light responsiveness |
| TaDUF966-4A | - | *Zea mays* | cis-acting regulatory element involved in light responsiveness |
| TaDUF966-4A | + | *Zea mays* | part of a light responsive element |
| TaDUF966-4A | - | *Hordeum vulgare* | cis-acting element involved in low-temperature responsiveness |
| TaDUF966-4A | + | *Oryza sativa* | light responsive element |
| TaDUF966-4A | + | *Oryza sativa* | core promoter element around -30 of transcription start |
| TaDUF966-4A | + | *Brassica oleracea* | core promoter element around -30 of transcription start |
| TaDUF966-4A | - | *Arabidopsis thaliana* | core promoter element around -30 of transcription start |
| TaDUF966-4A | - | *Hordeum vulgare* | cis-acting regulatory element involved in the MeJA-responsiveness |
| TaDUF966-4B | + | *Petroselinum crispum* | cis-acting regulatory element |
| TaDUF966-4B | - | *Arabidopsis thaliana* | cis-acting element involved in the abscisic acid responsiveness |
| TaDUF966-4B | - | *Zea mays* | cis-acting regulatory element essential for the anaerobic induction |
| TaDUF966-4B | + | *Petroselinum crispum* | part of a conserved DNA module involved in light responsiveness |
| TaDUF966-4B | - | *Pisum sativum* | common cis-acting element in promoter and enhancer regions |
| TaDUF966-4B | - | *Pisum sativum* | common cis-acting element in promoter and enhancer regions |
| TaDUF966-4B | + | *Pisum sativum* | common cis-acting element in promoter and enhancer regions |
| TaDUF966-4B | + | *Nicotiana glutinosa* |  |
| TaDUF966-4B | - | *Nicotiana glutinosa* |  |
| TaDUF966-4B | - | *Pisum sativum* | common cis-acting element in promoter and enhancer regions |
| TaDUF966-4B | + | *Pisum sativum* | common cis-acting element in promoter and enhancer regions |
| TaDUF966-4B | - | *Pisum sativum* | common cis-acting element in promoter and enhancer regions |
| TaDUF966-4B | - | *Nicotiana glutinosa* |  |
| TaDUF966-4B | + | *Nicotiana glutinosa* |  |
| TaDUF966-4B | + | *Nicotiana glutinosa* |  |
| TaDUF966-4B | - | *Nicotiana glutinosa* |  |
| TaDUF966-4B | + | *Pisum sativum* | common cis-acting element in promoter and enhancer regions |
| TaDUF966-4B | + | *Arabidopsis thaliana* | common cis-acting element in promoter and enhancer regions |
| TaDUF966-4B | + | *Nicotiana glutinosa* |  |
| TaDUF966-4B | - | *Nicotiana glutinosa* |  |
| TaDUF966-4B | + | *Nicotiana glutinosa* |  |
| TaDUF966-4B | - | *Nicotiana glutinosa* |  |
| TaDUF966-4B | + | *Arabidopsis thaliana* | common cis-acting element in promoter and enhancer regions |
| TaDUF966-4B | + | *Nicotiana glutinosa* |  |
| TaDUF966-4B | + | *Hordeum vulgare* | cis-acting regulatory element involved in the MeJA-responsiveness |
| TaDUF966-4B | - | *Solanum tuberosum* | part of a light responsive element |
| TaDUF966-4B | + | *Zea mays* | cis-acting regulatory element involved in light responsiveness |
| TaDUF966-4B | + | *Avena sativa* | light responsive element |
| TaDUF966-4B | - | *Zea mays* | part of a light responsive element |
| TaDUF966-4B | + | *Arabidopsis thaliana* | part of a light responsive element |
| TaDUF966-4B | - | *Zea mays* | cis-acting regulatory element involved in zein metabolism regulation |
| TaDUF966-4B | + | *Zea mays* | cis-acting regulatory element involved in zein metabolism regulation |
| TaDUF966-4B | + | *Oryza sativa* | gibberellin-responsive element |
| TaDUF966-4B | + | *Oryza sativa* | light responsive element |
| TaDUF966-4B | - | *Oryza sativa* | light responsive element |
| TaDUF966-4B | - | *Oryza sativa* | light responsive element |
| TaDUF966-4B | + | *Arabidopsis thaliana* | core promoter element around -30 of transcription start |
| TaDUF966-4B | - | *Brassica oleracea* | cis-acting element involved in salicylic acid responsiveness |
| TaDUF966-4B | + | *Nicotiana tabacum* | cis-acting element involved in salicylic acid responsiveness |
| TaDUF966-4B | - | *Hordeum vulgare* | cis-acting regulatory element involved in the MeJA-responsiveness |
| TaDUF966-4D | + | *Arabidopsis thaliana* | cis-acting element involved in the abscisic acid responsiveness |
| TaDUF966-4D | + | *Arabidopsis thaliana* | cis-acting element involved in the abscisic acid responsiveness |
| TaDUF966-4D | - | *Arabidopsis thaliana* | cis-acting element involved in the abscisic acid responsiveness |
| TaDUF966-4D | - | *Arabidopsis thaliana* | part of a module for light response |
| TaDUF966-4D | + | *Zea mays* | cis-acting regulatory element essential for the anaerobic induction |
| TaDUF966-4D | - | *Petroselinum crispum* | part of a conserved DNA module involved in light responsiveness |
| TaDUF966-4D | - | *Pisum sativum* | common cis-acting element in promoter and enhancer regions |
| TaDUF966-4D | + | *Nicotiana glutinosa* |  |
| TaDUF966-4D | + | *Nicotiana glutinosa* |  |
| TaDUF966-4D | + | *Nicotiana glutinosa* |  |
| TaDUF966-4D | - | *Nicotiana glutinosa* |  |
| TaDUF966-4D | + | *Pisum sativum* | common cis-acting element in promoter and enhancer regions |
| TaDUF966-4D | - | *Pisum sativum* | common cis-acting element in promoter and enhancer regions |
| TaDUF966-4D | - | *Pisum sativum* | common cis-acting element in promoter and enhancer regions |
| TaDUF966-4D | + | *Nicotiana glutinosa* |  |
| TaDUF966-4D | + | *Pisum sativum* | common cis-acting element in promoter and enhancer regions |
| TaDUF966-4D | + | *Nicotiana glutinosa* |  |
| TaDUF966-4D | + | *Nicotiana glutinosa* |  |
| TaDUF966-4D | - | *Nicotiana glutinosa* |  |
| TaDUF966-4D | + | *Nicotiana glutinosa* |  |
| TaDUF966-4D | - | *Nicotiana glutinosa* |  |
| TaDUF966-4D | - | *Pisum sativum* | common cis-acting element in promoter and enhancer regions |
| TaDUF966-4D | - | *Pisum sativum* | common cis-acting element in promoter and enhancer regions |
| TaDUF966-4D | + | *Nicotiana glutinosa* |  |
| TaDUF966-4D | + | *Hordeum vulgare* | MYBHv1 binding site |
| TaDUF966-4D | + | *Hordeum vulgare* | cis-acting regulatory element involved in the MeJA-responsiveness |
| TaDUF966-4D | + | *Arabidopsis thaliana* | part of a light responsive element |
| TaDUF966-4D | + | *Pisum sativum* | cis-acting regulatory element involved in light responsiveness |
| TaDUF966-4D | + | *Arabidopsis thaliana* | cis-acting regulatory element involved in light responsiveness |
| TaDUF966-4D | + | *Arabidopsis thaliana* | light responsive element |
| TaDUF966-4D | + | *Hordeum vulgare* | cis-acting element involved in low-temperature responsiveness |
| TaDUF966-4D | - | *Hordeum vulgare* | cis-acting element involved in low-temperature responsiveness |
| TaDUF966-4D | + | *Arabidopsis thaliana* | MYB binding site involved in drought-inducibility |
| TaDUF966-4D | + | *Petroselinum crispum* | MYB binding site involved in light responsiveness |
| TaDUF966-4D | + | *Oryza sativa* | gibberellin-responsive element |
| TaDUF966-4D | - | *Oryza sativa* | gibberellin-responsive element |
| TaDUF966-4D | - | *Arabidopsis thaliana* | core promoter element around -30 of transcription start |
| TaDUF966-4D | + | *Brassica napus* | core promoter element around -30 of transcription start |
| TaDUF966-4D | - | *Arabidopsis thaliana* | core promoter element around -30 of transcription start |
| TaDUF966-4D | + | *Arabidopsis thaliana* | core promoter element around -30 of transcription start |
| TaDUF966-4D | + | *Oryza sativa* | core promoter element around -30 of transcription start |
| TaDUF966-4D | - | *Arabidopsis thaliana* | core promoter element around -30 of transcription start |
| TaDUF966-4D | + | *Arabidopsis thaliana* | core promoter element around -30 of transcription start |
| TaDUF966-4D | + | *Oryza sativa* | core promoter element around -30 of transcription start |
| TaDUF966-4D | - | *Nicotiana tabacum* | cis-acting element involved in salicylic acid responsiveness |
| TaDUF966-4D | - | *Hordeum vulgare* | cis-acting regulatory element involved in the MeJA-responsiveness |
| TaDUF966-4D | + | *Brassica oleracea* | auxin-responsive element |
| TaDUF966-5A | - | *Arabidopsis thaliana* | cis-acting element involved in the abscisic acid responsiveness |
| TaDUF966-5A | - | *Arabidopsis thaliana* | cis-acting element involved in the abscisic acid responsiveness |
| TaDUF966-5A | + | *Arabidopsis thaliana* | cis-acting element involved in the abscisic acid responsiveness |
| TaDUF966-5A | + | *Arabidopsis thaliana* | cis-acting element involved in the abscisic acid responsiveness |
| TaDUF966-5A | - | *Oryza sativa* | cis-acting element involved in the abscisic acid responsiveness |
| TaDUF966-5A | - | *Arabidopsis thaliana* | cis-acting element involved in the abscisic acid responsiveness |
| TaDUF966-5A | - | *Arabidopsis thaliana* | cis-acting element involved in the abscisic acid responsiveness |
| TaDUF966-5A | + | *Arabidopsis thaliana* | part of a module for light response |
| TaDUF966-5A | + | *Pisum sativum* | common cis-acting element in promoter and enhancer regions |
| TaDUF966-5A | - | *Nicotiana glutinosa* |  |
| TaDUF966-5A | + | *Nicotiana glutinosa* |  |
| TaDUF966-5A | - | *Pisum sativum* | common cis-acting element in promoter and enhancer regions |
| TaDUF966-5A | - | *Nicotiana glutinosa* |  |
| TaDUF966-5A | - | *Nicotiana glutinosa* |  |
| TaDUF966-5A | - | *Nicotiana glutinosa* |  |
| TaDUF966-5A | - | *Pisum sativum* | common cis-acting element in promoter and enhancer regions |
| TaDUF966-5A | + | *Nicotiana glutinosa* |  |
| TaDUF966-5A | - | *Arabidopsis thaliana* | common cis-acting element in promoter and enhancer regions |
| TaDUF966-5A | - | *Arabidopsis thaliana* | cis-acting regulatory element related to meristem expression |
| TaDUF966-5A | - | *Hordeum vulgare* | MYBHv1 binding site |
| TaDUF966-5A | - | *Hordeum vulgare* | MYBHv1 binding site |
| TaDUF966-5A | + | *Hordeum vulgare* | cis-acting regulatory element involved in the MeJA-responsiveness |
| TaDUF966-5A | + | *Hordeum vulgare* | cis-acting regulatory element involved in the MeJA-responsiveness |
| TaDUF966-5A | + | *Hordeum vulgare* | cis-acting regulatory element involved in the MeJA-responsiveness |
| TaDUF966-5A | - | *Hordeum vulgare* | cis-acting regulatory element involved in the MeJA-responsiveness |
| TaDUF966-5A | + | *Hordeum vulgare* | cis-acting regulatory element involved in the MeJA-responsiveness |
| TaDUF966-5A | - | *Hordeum vulgare* | cis-acting regulatory element involved in the MeJA-responsiveness |
| TaDUF966-5A | + | *Pisum sativum* | cis-acting regulatory element involved in light responsiveness |
| TaDUF966-5A | + | *Pisum sativum* | cis-acting regulatory element involved in light responsiveness |
| TaDUF966-5A | + | *Pisum sativum* | cis-acting regulatory element involved in light responsiveness |
| TaDUF966-5A | + | *Zea mays* | cis-acting regulatory element involved in light responsiveness |
| TaDUF966-5A | + | *Arabidopsis thaliana* | cis-acting regulatory element involved in light responsiveness |
| TaDUF966-5A | + | *Zea mays* | cis-acting regulatory element involved in light responsiveness |
| TaDUF966-5A | - | *Zea mays* | enhancer-like element involved in anoxic specific inducibility |
| TaDUF966-5A | + | *Arabidopsis thaliana* | light responsive element |
| TaDUF966-5A | + | *Larix laricina* | part of a light responsive element |
| TaDUF966-5A | - | *Arabidopsis thaliana* | MYB binding site involved in drought-inducibility |
| TaDUF966-5A | + | *Arabidopsis thaliana* | MYB binding site involved in drought-inducibility |
| TaDUF966-5A | - | *Petroselinum crispum* | MYB binding site involved in light responsiveness |
| TaDUF966-5A | + | *Zea mays* | cis-acting regulatory element involved in zein metabolism regulation |
| TaDUF966-5A | - | *Zea mays* | cis-acting regulatory element involved in zein metabolism regulation |
| TaDUF966-5A | + | *Oryza sativa* | light responsive element |
| TaDUF966-5A | - | *Oryza sativa* | light responsive element |
| TaDUF966-5A | + | *Oryza sativa* | light responsive element |
| TaDUF966-5A | - | *Arabidopsis thaliana* | core promoter element around -30 of transcription start |
| TaDUF966-5A | - | *Arabidopsis thaliana* | core promoter element around -30 of transcription start |
| TaDUF966-5A | - | *Arabidopsis thaliana* | core promoter element around -30 of transcription start |
| TaDUF966-5A | - | *Arabidopsis thaliana* | core promoter element around -30 of transcription start |
| TaDUF966-5A | - | *Oryza sativa* | core promoter element around -30 of transcription start |
| TaDUF966-5A | - | *Arabidopsis thaliana* | core promoter element around -30 of transcription start |
| TaDUF966-5A | - | *Arabidopsis thaliana* | core promoter element around -30 of transcription start |
| TaDUF966-5A | - | *Pisum sativum* | core promoter element around -30 of transcription start |
| TaDUF966-5A | - | *Helianthus annuus* | core promoter element around -30 of transcription start |
| TaDUF966-5A | - | *Arabidopsis thaliana* | core promoter element around -30 of transcription start |
| TaDUF966-5A | - | *Arabidopsis thaliana* | core promoter element around -30 of transcription start |
| TaDUF966-5A | + | *Nicotiana tabacum* | cis-acting element involved in defense and stress responsiveness |
| TaDUF966-5A | - | *Hordeum vulgare* | cis-acting regulatory element involved in the MeJA-responsiveness |
| TaDUF966-5A | - | *Hordeum vulgare* | cis-acting regulatory element involved in the MeJA-responsiveness |
| TaDUF966-5A | - | *Hordeum vulgare* | cis-acting regulatory element involved in the MeJA-responsiveness |
| TaDUF966-5A | + | *Hordeum vulgare* | cis-acting regulatory element involved in the MeJA-responsiveness |
| TaDUF966-5A | - | *Hordeum vulgare* | cis-acting regulatory element involved in the MeJA-responsiveness |
| TaDUF966-5A | + | *Hordeum vulgare* | cis-acting regulatory element involved in the MeJA-responsiveness |
| TaDUF966-5A | + | *Brassica oleracea* | auxin-responsive element |
| TaDUF966-5B | - | *Petroselinum crispum* | cis-acting regulatory element |
| TaDUF966-5B | - | *Petroselinum crispum* | cis-acting regulatory element |
| TaDUF966-5B | - | *Petroselinum crispum* | cis-acting regulatory element |
| TaDUF966-5B | + | *Arabidopsis thaliana* | cis-acting element involved in the abscisic acid responsiveness |
| TaDUF966-5B | + | *Arabidopsis thaliana* | cis-acting element involved in the abscisic acid responsiveness |
| TaDUF966-5B | - | *Oryza sativa* | cis-acting element involved in the abscisic acid responsiveness |
| TaDUF966-5B | + | *Petroselinum crispum* | part of a conserved DNA module involved in light responsiveness |
| TaDUF966-5B | + | *Nicotiana glutinosa* |  |
| TaDUF966-5B | - | *Pisum sativum* | common cis-acting element in promoter and enhancer regions |
| TaDUF966-5B | - | *Pisum sativum* | common cis-acting element in promoter and enhancer regions |
| TaDUF966-5B | - | *Nicotiana glutinosa* |  |
| TaDUF966-5B | + | *Nicotiana glutinosa* |  |
| TaDUF966-5B | - | *Pisum sativum* | common cis-acting element in promoter and enhancer regions |
| TaDUF966-5B | - | *Arabidopsis thaliana* | common cis-acting element in promoter and enhancer regions |
| TaDUF966-5B | + | *Nicotiana glutinosa* |  |
| TaDUF966-5B | + | *Pisum sativum* | common cis-acting element in promoter and enhancer regions |
| TaDUF966-5B | + | *Nicotiana glutinosa* |  |
| TaDUF966-5B | + | *Arabidopsis thaliana* | cis-acting regulatory element related to meristem expression |
| TaDUF966-5B | - | *Hordeum vulgare* | MYBHv1 binding site |
| TaDUF966-5B | + | *Hordeum vulgare* | cis-acting regulatory element involved in the MeJA-responsiveness |
| TaDUF966-5B | - | *Hordeum vulgare* | cis-acting regulatory element involved in the MeJA-responsiveness |
| TaDUF966-5B | - | *Pisum sativum* | part of a light responsive element |
| TaDUF966-5B | + | *Zea mays* | cis-acting regulatory element involved in light responsiveness |
| TaDUF966-5B | + | *Zea mays* | cis-acting regulatory element involved in light responsiveness |
| TaDUF966-5B | - | *Zea mays* | cis-acting regulatory element involved in light responsiveness |
| TaDUF966-5B | - | *Zea mays* | cis-acting regulatory element involved in light responsiveness |
| TaDUF966-5B | + | *Zea mays* | cis-acting regulatory element involved in zein metabolism regulation |
| TaDUF966-5B | - | *Brassica napus* | core promoter element around -30 of transcription start |
| TaDUF966-5B | - | *Arabidopsis thaliana* | core promoter element around -30 of transcription start |
| TaDUF966-5B | - | *Pisum sativum* | core promoter element around -30 of transcription start |
| TaDUF966-5B | - | *Helianthus annuus* | core promoter element around -30 of transcription start |
| TaDUF966-5B | - | *Arabidopsis thaliana* | core promoter element around -30 of transcription start |
| TaDUF966-5B | - | *Arabidopsis thaliana* | core promoter element around -30 of transcription start |
| TaDUF966-5B | - | *Arabidopsis thaliana* | core promoter element around -30 of transcription start |
| TaDUF966-5B | - | *Arabidopsis thaliana* | core promoter element around -30 of transcription start |
| TaDUF966-5B | - | *Hordeum vulgare* | cis-acting regulatory element involved in the MeJA-responsiveness |
| TaDUF966-5B | + | *Hordeum vulgare* | cis-acting regulatory element involved in the MeJA-responsiveness |
| TaDUF966-5B | + | *Brassica oleracea* | auxin-responsive element |
| TaDUF966-5D | - | *Oryza sativa* | cis-acting element involved in the abscisic acid responsiveness |
| TaDUF966-5D | - | *Arabidopsis thaliana* | cis-acting element involved in the abscisic acid responsiveness |
| TaDUF966-5D | - | *Arabidopsis thaliana* | common cis-acting element in promoter and enhancer regions |
| TaDUF966-5D | - | *Arabidopsis thaliana* | common cis-acting element in promoter and enhancer regions |
| TaDUF966-5D | + | *Nicotiana glutinosa* |  |
| TaDUF966-5D | + | *Nicotiana glutinosa* |  |
| TaDUF966-5D | + | *Pisum sativum* | common cis-acting element in promoter and enhancer regions |
| TaDUF966-5D | + | *Nicotiana glutinosa* |  |
| TaDUF966-5D | - | *Arabidopsis thaliana* | common cis-acting element in promoter and enhancer regions |
| TaDUF966-5D | - | *Nicotiana glutinosa* |  |
| TaDUF966-5D | + | *Pisum sativum* | common cis-acting element in promoter and enhancer regions |
| TaDUF966-5D | + | *Arabidopsis thaliana* | common cis-acting element in promoter and enhancer regions |
| TaDUF966-5D | + | *Nicotiana glutinosa* |  |
| TaDUF966-5D | - | *Pisum sativum* | common cis-acting element in promoter and enhancer regions |
| TaDUF966-5D | - | *Arabidopsis thaliana* | common cis-acting element in promoter and enhancer regions |
| TaDUF966-5D | + | *Pisum sativum* | common cis-acting element in promoter and enhancer regions |
| TaDUF966-5D | + | *Arabidopsis thaliana* | cis-acting regulatory element related to meristem expression |
| TaDUF966-5D | - | *Hordeum vulgare* | MYBHv1 binding site |
| TaDUF966-5D | + | *Zea mays* | cis-acting regulatory element involved in light responsiveness |
| TaDUF966-5D | + | *Arabidopsis thaliana* | MYB binding site involved in drought-inducibility |
| TaDUF966-5D | + | *Arabidopsis thaliana* | core promoter element around -30 of transcription start |
| TaDUF966-5D | - | *Brassica napus* | core promoter element around -30 of transcription start |
| TaDUF966-5D | - | *Arabidopsis thaliana* | core promoter element around -30 of transcription start |
| TaDUF966-5D | - | *Pisum sativum* | core promoter element around -30 of transcription start |
| TaDUF966-5D | - | *Helianthus annuus* | core promoter element around -30 of transcription start |
| TaDUF966-5D | - | *Arabidopsis thaliana* | core promoter element around -30 of transcription start |
| TaDUF966-5D | - | *Arabidopsis thaliana* | core promoter element around -30 of transcription start |
| TaDUF966-5D | + | *Brassica oleracea* | core promoter element around -30 of transcription start |
| TaDUF966-5D | - | *Arabidopsis thaliana* | core promoter element around -30 of transcription start |
| TaDUF966-6B | - | *Arabidopsis thaliana* | cis-acting element involved in the abscisic acid responsiveness |
| TaDUF966-6B | + | *Arabidopsis thaliana* | cis-acting element involved in the abscisic acid responsiveness |
| TaDUF966-6B | + | *Arabidopsis thaliana* | part of a module for light response |
| TaDUF966-6B | - | *Petroselinum crispum* | part of a conserved DNA module involved in light responsiveness |
| TaDUF966-6B | - | *Nicotiana glutinosa* |  |
| TaDUF966-6B | + | *Pisum sativum* | common cis-acting element in promoter and enhancer regions |
| TaDUF966-6B | + | *Pisum sativum* | common cis-acting element in promoter and enhancer regions |
| TaDUF966-6B | + | *Pisum sativum* | common cis-acting element in promoter and enhancer regions |
| TaDUF966-6B | - | *Nicotiana glutinosa* |  |
| TaDUF966-6B | + | *Pisum sativum* | common cis-acting element in promoter and enhancer regions |
| TaDUF966-6B | - | *Pisum sativum* | common cis-acting element in promoter and enhancer regions |
| TaDUF966-6B | + | *Nicotiana glutinosa* |  |
| TaDUF966-6B | + | *Nicotiana glutinosa* |  |
| TaDUF966-6B | + | *Nicotiana glutinosa* |  |
| TaDUF966-6B | - | *Pisum sativum* | common cis-acting element in promoter and enhancer regions |
| TaDUF966-6B | - | *Nicotiana glutinosa* |  |
| TaDUF966-6B | + | *Pisum sativum* | common cis-acting element in promoter and enhancer regions |
| TaDUF966-6B | - | *Nicotiana glutinosa* |  |
| TaDUF966-6B | - | *Nicotiana glutinosa* |  |
| TaDUF966-6B | - | *Nicotiana glutinosa* |  |
| TaDUF966-6B | - | *Nicotiana glutinosa* |  |
| TaDUF966-6B | + | *Nicotiana glutinosa* |  |
| TaDUF966-6B | + | *Nicotiana glutinosa* |  |
| TaDUF966-6B | - | *Pisum sativum* | common cis-acting element in promoter and enhancer regions |
| TaDUF966-6B | + | *Nicotiana glutinosa* |  |
| TaDUF966-6B | + | *Petunia hybrida* | common cis-acting element in promoter and enhancer regions |
| TaDUF966-6B | - | *Pisum sativum* | common cis-acting element in promoter and enhancer regions |
| TaDUF966-6B | + | *Nicotiana glutinosa* |  |
| TaDUF966-6B | + | *Nicotiana glutinosa* |  |
| TaDUF966-6B | - | *Pisum sativum* | common cis-acting element in promoter and enhancer regions |
| TaDUF966-6B | - | *Nicotiana glutinosa* |  |
| TaDUF966-6B | - | *Nicotiana glutinosa* |  |
| TaDUF966-6B | - | *Arabidopsis thaliana* | common cis-acting element in promoter and enhancer regions |
| TaDUF966-6B | - | *Arabidopsis thaliana* | cis-acting regulatory element related to meristem expression |
| TaDUF966-6B | + | *Hordeum vulgare* | MYBHv1 binding site |
| TaDUF966-6B | - | *Hordeum vulgare* | cis-acting regulatory element involved in the MeJA-responsiveness |
| TaDUF966-6B | - | *Pisum sativum* | cis-acting regulatory element involved in light responsiveness |
| TaDUF966-6B | - | *Arabidopsis thaliana* | cis-acting regulatory element involved in light responsiveness |
| TaDUF966-6B | - | *Arabidopsis thaliana* | light responsive element |
| TaDUF966-6B | + | *Solanum tuberosum* | part of a light responsive element |
| TaDUF966-6B | + | *Arabidopsis thaliana* | core promoter element around -30 of transcription start |
| TaDUF966-6B | + | *Brassica oleracea* | core promoter element around -30 of transcription start |
| TaDUF966-6B | + | *Arabidopsis thaliana* | core promoter element around -30 of transcription start |
| TaDUF966-6B | + | *Arabidopsis thaliana* | core promoter element around -30 of transcription start |
| TaDUF966-6B | - | *Brassica juncea* | core promoter element around -30 of transcription start |
| TaDUF966-6B | - | *Helianthus annuus* | core promoter element around -30 of transcription start |
| TaDUF966-6B | - | *Arabidopsis thaliana* | core promoter element around -30 of transcription start |
| TaDUF966-6B | + | *Arabidopsis thaliana* | core promoter element around -30 of transcription start |
| TaDUF966-6B | + | *Brassica napus* | core promoter element around -30 of transcription start |
| TaDUF966-6B | + | *Arabidopsis thaliana* | core promoter element around -30 of transcription start |
| TaDUF966-6B | + | *Arabidopsis thaliana* | core promoter element around -30 of transcription start |
| TaDUF966-6B | - | *Arabidopsis thaliana* | core promoter element around -30 of transcription start |
| TaDUF966-6B | - | *Arabidopsis thaliana* | core promoter element around -30 of transcription start |
| TaDUF966-6B | - | *Arabidopsis thaliana* | core promoter element around -30 of transcription start |
| TaDUF966-6B | + | *Brassica napus* | core promoter element around -30 of transcription start |
| TaDUF966-6B | - | *Arabidopsis thaliana* | core promoter element around -30 of transcription start |
| TaDUF966-6B | - | *Arabidopsis thaliana* | core promoter element around -30 of transcription start |
| TaDUF966-6B | - | *Arabidopsis thaliana* | core promoter element around -30 of transcription start |
| TaDUF966-6B | - | *Arabidopsis thaliana* | core promoter element around -30 of transcription start |
| TaDUF966-6B | - | *Arabidopsis thaliana* | core promoter element around -30 of transcription start |
| TaDUF966-6B | - | *Helianthus annuus* | core promoter element around -30 of transcription start |
| TaDUF966-6B | - | *Arabidopsis thaliana* | core promoter element around -30 of transcription start |
| TaDUF966-6B | - | *Helianthus annuus* | core promoter element around -30 of transcription start |
| TaDUF966-6B | - | *Arabidopsis thaliana* | core promoter element around -30 of transcription start |
| TaDUF966-6B | - | *Brassica napus* | core promoter element around -30 of transcription start |
| TaDUF966-6B | - | *Arabidopsis thaliana* | core promoter element around -30 of transcription start |
| TaDUF966-6B | + | *Avena sativa* | core promoter element around -30 of transcription start |
| TaDUF966-6B | - | *Daucus carota* | core promoter element around -30 of transcription start |
| TaDUF966-6B | - | *Brassica juncea* | core promoter element around -30 of transcription start |
| TaDUF966-6B | - | *Helianthus annuus* | core promoter element around -30 of transcription start |
| TaDUF966-6B | - | *Arabidopsis thaliana* | core promoter element around -30 of transcription start |
| TaDUF966-6B | - | *Arabidopsis thaliana* | core promoter element around -30 of transcription start |
| TaDUF966-6B | - | *Arabidopsis thaliana* | core promoter element around -30 of transcription start |
| TaDUF966-6B | - | *Arabidopsis thaliana* | core promoter element around -30 of transcription start |
| TaDUF966-6B | - | *Brassica napus* | core promoter element around -30 of transcription start |
| TaDUF966-6B | - | *Arabidopsis thaliana* | core promoter element around -30 of transcription start |
| TaDUF966-6B | - | *Brassica napus* | core promoter element around -30 of transcription start |
| TaDUF966-6B | - | *Arabidopsis thaliana* | core promoter element around -30 of transcription start |
| TaDUF966-6B | - | *Arabidopsis thaliana* | core promoter element around -30 of transcription start |
| TaDUF966-6B | - | *Arabidopsis thaliana* | core promoter element around -30 of transcription start |
| TaDUF966-6B | - | *Arabidopsis thaliana* | core promoter element around -30 of transcription start |
| TaDUF966-6B | - | *Arabidopsis thaliana* | core promoter element around -30 of transcription start |
| TaDUF966-6B | + | *Nicotiana tabacum* | cis-acting element involved in defense and stress responsiveness |
| TaDUF966-6B | + | *Nicotiana tabacum* | cis-acting element involved in defense and stress responsiveness |
| TaDUF966-6B | + | *Arabidopsis thaliana* | part of a light responsive element |
| TaDUF966-6B | + | *Hordeum vulgare* | cis-acting regulatory element involved in the MeJA-responsiveness |
| TaDUF966-7D | - | *Zea mays* | enhancer-like element involved in anoxic specific inducibility |
| TaDUF966-7D | - | *Zea mays* | enhancer-like element involved in anoxic specific inducibility |
| TaDUF966-7D | - | *Zea mays* | enhancer-like element involved in anoxic specific inducibility |
| TaDUF966-7D | - | *Zea mays* | enhancer-like element involved in anoxic specific inducibility |
| TaDUF966-7D | - | *Zea mays* | enhancer-like element involved in anoxic specific inducibility |
| TaDUF966-7D | - | *Zea mays* | enhancer-like element involved in anoxic specific inducibility |
| TaDUF966-7D | + | *Zea mays* | cis-acting regulatory element involved in zein metabolism regulation |
| TaDUF966-7D | - | *Arabidopsis thaliana* | cis-acting regulatory element related to meristem expression |
| TaDUF966-7D | + | *Arabidopsis thaliana* | core promoter element around -30 of transcription start |
| TaDUF966-7D | + | *Arabidopsis thaliana* | core promoter element around -30 of transcription start |
| TaDUF966-7D | - | *Arabidopsis thaliana* | core promoter element around -30 of transcription start |
| TaDUF966-7D | - | *Helianthus annuus* | core promoter element around -30 of transcription start |
| TaDUF966-7D | - | *Arabidopsis thaliana* | core promoter element around -30 of transcription start |
| TaDUF966-7D | + | *Arabidopsis thaliana* | core promoter element around -30 of transcription start |
| TaDUF966-7D | + | *Arabidopsis thaliana* | core promoter element around -30 of transcription start |
| TaDUF966-7D | - | *Arabidopsis thaliana* | core promoter element around -30 of transcription start |
| TaDUF966-7D | - | *Arabidopsis thaliana* | core promoter element around -30 of transcription start |
| TaDUF966-7D | - | *Arabidopsis thaliana* | core promoter element around -30 of transcription start |
| TaDUF966-7D | - | *Nicotiana glutinosa* |  |
| TaDUF966-7D | + | *Nicotiana glutinosa* |  |
| TaDUF966-7D | + | *Arabidopsis thaliana* | common cis-acting element in promoter and enhancer regions |
| TaDUF966-7D | + | *Nicotiana glutinosa* |  |
| TaDUF966-7D | + | *Nicotiana glutinosa* |  |
| TaDUF966-7D | - | *Nicotiana glutinosa* |  |
| TaDUF966-7D | - | *Nicotiana glutinosa* |  |
| TaDUF966-7D | - | *Nicotiana glutinosa* |  |
| TaDUF966-7D | + | *Nicotiana glutinosa* |  |
| TaDUF966-7D | + | *Nicotiana glutinosa* |  |
| TaDUF966-7D | + | *Pisum sativum* | common cis-acting element in promoter and enhancer regions |
| TaDUF966-7D | + | *Pisum sativum* | common cis-acting element in promoter and enhancer regions |
| TaDUF966-7D | - | *Zea mays* | cis-acting regulatory element involved in light responsiveness |
| TaDUF966-7D | - | *Zea mays* | cis-acting regulatory element involved in light responsiveness |
| TaDUF966-7D | + | *Hordeum vulgare* | cis-acting regulatory element involved in the MeJA-responsiveness |
| TaDUF966-7D | + | *Oryza sativa* | light responsive element |
| TaDUF966-7D | + | *Oryza sativa* | light responsive element |
| TaDUF966-7D | - | *Petroselinum crispum* | cis-acting regulatory element |
| TaDUF966-7D | + | *Hordeum vulgare* | MYBHv1 binding site |
| TaDUF966-7D | - | *Hordeum vulgare* | cis-acting regulatory element involved in the MeJA-responsiveness |
| TaDUF966-7D | - | *Arabidopsis thaliana* | light responsive element |
| TaDUF966-7D | - | *Zea mays* | cis-acting regulatory element essential for the anaerobic induction |
| TaDUF966-7D | - | *Zea mays* | cis-acting regulatory element essential for the anaerobic induction |
| TaDUF966-7D | - | *Zea mays* | cis-acting regulatory element essential for the anaerobic induction |
| TaDUF966-7D | + | *Arabidopsis thaliana* | MYB binding site involved in drought-inducibility |
| TaDUF966-8A | + | *Nicotiana glutinosa* |  |
| TaDUF966-8A | + | *Pisum sativum* | common cis-acting element in promoter and enhancer regions |
| TaDUF966-8A | + | *Nicotiana glutinosa* |  |
| TaDUF966-8A | - | *Nicotiana glutinosa* |  |
| TaDUF966-8A | + | *Nicotiana glutinosa* |  |
| TaDUF966-8A | - | *Pisum sativum* | common cis-acting element in promoter and enhancer regions |
| TaDUF966-8A | - | *Nicotiana glutinosa* |  |
| TaDUF966-8A | + | *Nicotiana glutinosa* |  |
| TaDUF966-8A | + | *Nicotiana glutinosa* |  |
| TaDUF966-8A | - | *Arabidopsis thaliana* | common cis-acting element in promoter and enhancer regions |
| TaDUF966-8A | - | *Nicotiana glutinosa* |  |
| TaDUF966-8A | - | *Arabidopsis thaliana* | common cis-acting element in promoter and enhancer regions |
| TaDUF966-8A | - | *Pisum sativum* | common cis-acting element in promoter and enhancer regions |
| TaDUF966-8A | + | *Pisum sativum* | common cis-acting element in promoter and enhancer regions |
| TaDUF966-8A | + | *Pisum sativum* | common cis-acting element in promoter and enhancer regions |
| TaDUF966-8A | - | *Nicotiana glutinosa* |  |
| TaDUF966-8A | + | *Pisum sativum* | common cis-acting element in promoter and enhancer regions |
| TaDUF966-8A | + | *Nicotiana glutinosa* |  |
| TaDUF966-8A | + | *Nicotiana glutinosa* |  |
| TaDUF966-8A | - | *Arabidopsis thaliana* | common cis-acting element in promoter and enhancer regions |
| TaDUF966-8A | - | *Pisum sativum* | common cis-acting element in promoter and enhancer regions |
| TaDUF966-8A | - | *Arabidopsis thaliana* | cis-acting regulatory element related to meristem expression |
| TaDUF966-8A | - | *Hordeum vulgare* | cis-acting regulatory element involved in the MeJA-responsiveness |
| TaDUF966-8A | - | *Arabidopsis thaliana* | core promoter element around -30 of transcription start |
| TaDUF966-8A | + | *Arabidopsis thaliana* | core promoter element around -30 of transcription start |
| TaDUF966-8A | + | *Arabidopsis thaliana* | core promoter element around -30 of transcription start |
| TaDUF966-8A | + | *Brassica oleracea* | core promoter element around -30 of transcription start |
| TaDUF966-8A | + | *Arabidopsis thaliana* | core promoter element around -30 of transcription start |
| TaDUF966-8A | + | *Brassica oleracea* | core promoter element around -30 of transcription start |
| TaDUF966-8A | + | *Arabidopsis thaliana* | core promoter element around -30 of transcription start |
| TaDUF966-8A | + | *Arabidopsis thaliana* | core promoter element around -30 of transcription start |
| TaDUF966-8A | - | *Zea mays* | core promoter element around -30 of transcription start |
| TaDUF966-8A | - | *Arabidopsis thaliana* | core promoter element around -30 of transcription start |
| TaDUF966-8A | - | *Arabidopsis thaliana* | core promoter element around -30 of transcription start |
| TaDUF966-8A | - | *Arabidopsis thaliana* | core promoter element around -30 of transcription start |
| TaDUF966-8A | - | *Helianthus annuus* | core promoter element around -30 of transcription start |
| TaDUF966-8A | - | *Arabidopsis thaliana* | core promoter element around -30 of transcription start |
| TaDUF966-8A | + | *Nicotiana tabacum* | cis-acting element involved in salicylic acid responsiveness |
| TaDUF966-8A | + | *Brassica oleracea* | cis-acting element involved in salicylic acid responsiveness |
| TaDUF966-8A | - | *Nicotiana tabacum* | cis-acting element involved in salicylic acid responsiveness |
| TaDUF966-8A | + | *Hordeum vulgare* | cis-acting regulatory element involved in the MeJA-responsiveness |
| TaDUF966-8B | - | *Petroselinum crispum* | cis-acting regulatory element |
| TaDUF966-8B | - | *Petroselinum crispum* | cis-acting regulatory element |
| TaDUF966-8B | - | *Petroselinum crispum* | cis-acting regulatory element |
| TaDUF966-8B | + | *Arabidopsis thaliana* | cis-acting element involved in the abscisic acid responsiveness |
| TaDUF966-8B | - | *Arabidopsis thaliana* | cis-acting element involved in the abscisic acid responsiveness |
| TaDUF966-8B | + | *Arabidopsis thaliana* | part of a module for light response |
| TaDUF966-8B | + | *Arabidopsis thaliana* | part of a module for light response |
| TaDUF966-8B | - | *Arabidopsis thaliana* | part of a module for light response |
| TaDUF966-8B | + | *Arabidopsis thaliana* | part of a module for light response |
| TaDUF966-8B | - | *Zea mays* | cis-acting regulatory element essential for the anaerobic induction |
| TaDUF966-8B | + | *Zea mays* | cis-acting regulatory element essential for the anaerobic induction |
| TaDUF966-8B | - | *Nicotiana glutinosa* |  |
| TaDUF966-8B | + | *Pisum sativum* | common cis-acting element in promoter and enhancer regions |
| TaDUF966-8B | + | *Nicotiana glutinosa* |  |
| TaDUF966-8B | + | *Arabidopsis thaliana* | common cis-acting element in promoter and enhancer regions |
| TaDUF966-8B | + | *Nicotiana glutinosa* |  |
| TaDUF966-8B | - | *Arabidopsis thaliana* | common cis-acting element in promoter and enhancer regions |
| TaDUF966-8B | + | *Arabidopsis thaliana* | common cis-acting element in promoter and enhancer regions |
| TaDUF966-8B | + | *Nicotiana glutinosa* |  |
| TaDUF966-8B | + | *Nicotiana glutinosa* |  |
| TaDUF966-8B | + | *Pisum sativum* | common cis-acting element in promoter and enhancer regions |
| TaDUF966-8B | + | *Nicotiana glutinosa* |  |
| TaDUF966-8B | - | *Pisum sativum* | common cis-acting element in promoter and enhancer regions |
| TaDUF966-8B | + | *Arabidopsis thaliana* | common cis-acting element in promoter and enhancer regions |
| TaDUF966-8B | + | *Nicotiana glutinosa* |  |
| TaDUF966-8B | - | *Nicotiana glutinosa* |  |
| TaDUF966-8B | - | *Arabidopsis thaliana* | cis-acting regulatory element related to meristem expression |
| TaDUF966-8B | - | *Hordeum vulgare* | cis-acting regulatory element involved in the MeJA-responsiveness |
| TaDUF966-8B | - | *Hordeum vulgare* | cis-acting regulatory element involved in the MeJA-responsiveness |
| TaDUF966-8B | - | *Hordeum vulgare* | cis-acting regulatory element involved in the MeJA-responsiveness |
| TaDUF966-8B | - | *Pisum sativum* | part of a light responsive element |
| TaDUF966-8B | - | *Arabidopsis thaliana* | part of a light responsive element |
| TaDUF966-8B | - | *Zea mays* | cis-acting regulatory element involved in light responsiveness |
| TaDUF966-8B | - | *Arabidopsis thaliana* | cis-acting regulatory element involved in light responsiveness |
| TaDUF966-8B | - | *Zea mays* | cis-acting regulatory element involved in zein metabolism regulation |
| TaDUF966-8B | - | *Oryza sativa* | light responsive element |
| TaDUF966-8B | - | *Oryza sativa* | light responsive element |
| TaDUF966-8B | + | *Oryza sativa* | light responsive element |
| TaDUF966-8B | - | *Arabidopsis thaliana* | core promoter element around -30 of transcription start |
| TaDUF966-8B | + | *Brassica oleracea* | core promoter element around -30 of transcription start |
| TaDUF966-8B | - | *Arabidopsis thaliana* | core promoter element around -30 of transcription start |
| TaDUF966-8B | + | *Hordeum vulgare* | cis-acting regulatory element involved in the MeJA-responsiveness |
| TaDUF966-8B | + | *Hordeum vulgare* | cis-acting regulatory element involved in the MeJA-responsiveness |
| TaDUF966-8B | + | *Hordeum vulgare* | cis-acting regulatory element involved in the MeJA-responsiveness |
| TaDUF966-8D | - | *Petroselinum crispum* | cis-acting regulatory element |
| TaDUF966-8D | - | *Petroselinum crispum* | cis-acting regulatory element |
| TaDUF966-8D | - | *Petroselinum crispum* | cis-acting regulatory element |
| TaDUF966-8D | + | *Arabidopsis thaliana* | cis-acting element involved in the abscisic acid responsiveness |
| TaDUF966-8D | + | *Arabidopsis thaliana* | cis-acting element involved in the abscisic acid responsiveness |
| TaDUF966-8D | + | *Arabidopsis thaliana* | cis-acting element involved in the abscisic acid responsiveness |
| TaDUF966-8D | - | *Arabidopsis thaliana* | cis-acting element involved in the abscisic acid responsiveness |
| TaDUF966-8D | + | *Arabidopsis thaliana* | part of a module for light response |
| TaDUF966-8D | - | *Arabidopsis thaliana* | part of a module for light response |
| TaDUF966-8D | + | *Arabidopsis thaliana* | part of a module for light response |
| TaDUF966-8D | - | *Zea mays* | cis-acting regulatory element essential for the anaerobic induction |
| TaDUF966-8D | + | *Pisum sativum* | common cis-acting element in promoter and enhancer regions |
| TaDUF966-8D | + | *Nicotiana glutinosa* |  |
| TaDUF966-8D | + | *Arabidopsis thaliana* | common cis-acting element in promoter and enhancer regions |
| TaDUF966-8D | + | *Nicotiana glutinosa* |  |
| TaDUF966-8D | + | *Nicotiana glutinosa* |  |
| TaDUF966-8D | + | *Pisum sativum* | common cis-acting element in promoter and enhancer regions |
| TaDUF966-8D | - | *Pisum sativum* | common cis-acting element in promoter and enhancer regions |
| TaDUF966-8D | + | *Arabidopsis thaliana* | common cis-acting element in promoter and enhancer regions |
| TaDUF966-8D | + | *Nicotiana glutinosa* |  |
| TaDUF966-8D | - | *Nicotiana glutinosa* |  |
| TaDUF966-8D | - | *Arabidopsis thaliana* | cis-acting regulatory element related to meristem expression |
| TaDUF966-8D | - | *Hordeum vulgare* | cis-acting regulatory element involved in the MeJA-responsiveness |
| TaDUF966-8D | - | *Hordeum vulgare* | cis-acting regulatory element involved in the MeJA-responsiveness |
| TaDUF966-8D | - | *Hordeum vulgare* | cis-acting regulatory element involved in the MeJA-responsiveness |
| TaDUF966-8D | - | *Arabidopsis thaliana* | part of a light responsive element |
| TaDUF966-8D | + | *Pisum sativum* | cis-acting regulatory element involved in light responsiveness |
| TaDUF966-8D | + | *Arabidopsis thaliana* | cis-acting regulatory element involved in light responsiveness |
| TaDUF966-8D | - | *Zea mays* | cis-acting regulatory element involved in light responsiveness |
| TaDUF966-8D | - | *Arabidopsis thaliana* | cis-acting regulatory element involved in light responsiveness |
| TaDUF966-8D | + | *Hordeum vulgare* | cis-acting element involved in low-temperature responsiveness |
| TaDUF966-8D | - | *Zea mays* | cis-acting regulatory element involved in zein metabolism regulation |
| TaDUF966-8D | + | *Oryza sativa* | light responsive element |
| TaDUF966-8D | - | *Oryza sativa* | light responsive element |
| TaDUF966-8D | - | *Oryza sativa* | light responsive element |
| TaDUF966-8D | + | *Oryza sativa* | light responsive element |
| TaDUF966-8D | - | *Arabidopsis thaliana* | core promoter element around -30 of transcription start |
| TaDUF966-8D | + | *Brassica oleracea* | core promoter element around -30 of transcription start |
| TaDUF966-8D | - | *Arabidopsis thaliana* | core promoter element around -30 of transcription start |
| TaDUF966-8D | + | *Hordeum vulgare* | cis-acting regulatory element involved in the MeJA-responsiveness |
| TaDUF966-8D | + | *Hordeum vulgare* | cis-acting regulatory element involved in the MeJA-responsiveness |
| TaDUF966-8D | + | *Hordeum vulgare* | cis-acting regulatory element involved in the MeJA-responsiveness |
| TaDUF966-9A | + | *Petroselinum crispum* | cis-acting regulatory element |
| TaDUF966-9A | + | *Petroselinum crispum* | cis-acting regulatory element |
| TaDUF966-9A | + | *Petroselinum crispum* | cis-acting regulatory element |
| TaDUF966-9A | + | *Petroselinum crispum* | cis-acting regulatory element |
| TaDUF966-9A | + | *Petroselinum crispum* | cis-acting regulatory element |
| TaDUF966-9A | - | *Petroselinum crispum* | cis-acting regulatory element |
| TaDUF966-9A | + | *Petroselinum crispum* | cis-acting regulatory element |
| TaDUF966-9A | - | *Petroselinum crispum* | cis-acting regulatory element |
| TaDUF966-9A | - | *Arabidopsis thaliana* | cis-acting element involved in the abscisic acid responsiveness |
| TaDUF966-9A | + | *Petroselinum crispum* | part of a conserved DNA module involved in light responsiveness |
| TaDUF966-9A | + | *Arabidopsis thaliana* | common cis-acting element in promoter and enhancer regions |
| TaDUF966-9A | + | *Nicotiana glutinosa* |  |
| TaDUF966-9A | + | *Arabidopsis thaliana* | common cis-acting element in promoter and enhancer regions |
| TaDUF966-9A | + | *Nicotiana glutinosa* |  |
| TaDUF966-9A | - | *Nicotiana glutinosa* |  |
| TaDUF966-9A | + | *Nicotiana glutinosa* |  |
| TaDUF966-9A | + | *Nicotiana glutinosa* |  |
| TaDUF966-9A | - | *Nicotiana glutinosa* |  |
| TaDUF966-9A | + | *Arabidopsis thaliana* | common cis-acting element in promoter and enhancer regions |
| TaDUF966-9A | + | *Nicotiana glutinosa* |  |
| TaDUF966-9A | - | *Nicotiana glutinosa* |  |
| TaDUF966-9A | - | *Nicotiana glutinosa* |  |
| TaDUF966-9A | + | *Arabidopsis thaliana* | common cis-acting element in promoter and enhancer regions |
| TaDUF966-9A | + | *Nicotiana glutinosa* |  |
| TaDUF966-9A | - | *Nicotiana glutinosa* |  |
| TaDUF966-9A | - | *Nicotiana glutinosa* |  |
| TaDUF966-9A | + | *Hordeum vulgare* | cis-acting regulatory element involved in the MeJA-responsiveness |
| TaDUF966-9A | + | *Hordeum vulgare* | cis-acting regulatory element involved in the MeJA-responsiveness |
| TaDUF966-9A | - | *Hordeum vulgare* | cis-acting regulatory element involved in the MeJA-responsiveness |
| TaDUF966-9A | - | *Hordeum vulgare* | cis-acting regulatory element involved in the MeJA-responsiveness |
| TaDUF966-9A | - | *Arabidopsis thaliana* | cis-acting regulatory element involved in light responsiveness |
| TaDUF966-9A | + | *Zea mays* | enhancer-like element involved in anoxic specific inducibility |
| TaDUF966-9A | - | *Zea mays* | enhancer-like element involved in anoxic specific inducibility |
| TaDUF966-9A | + | *Hordeum vulgare* | cis-acting element involved in low-temperature responsiveness |
| TaDUF966-9A | - | *Oryza sativa* | light responsive element |
| TaDUF966-9A | - | *Oryza sativa* | light responsive element |
| TaDUF966-9A | - | *Oryza sativa* | light responsive element |
| TaDUF966-9A | + | *Nicotiana tabacum* | cis-acting element involved in defense and stress responsiveness |
| TaDUF966-9A | - | *Hordeum vulgare* | cis-acting regulatory element involved in the MeJA-responsiveness |
| TaDUF966-9A | - | *Hordeum vulgare* | cis-acting regulatory element involved in the MeJA-responsiveness |
| TaDUF966-9A | + | *Hordeum vulgare* | cis-acting regulatory element involved in the MeJA-responsiveness |
| TaDUF966-9A | + | *Hordeum vulgare* | cis-acting regulatory element involved in the MeJA-responsiveness |
| TaDUF966-9B | - | *Petroselinum crispum* | cis-acting regulatory element |
| TaDUF966-9B | + | *Arabidopsis thaliana* | cis-acting element involved in the abscisic acid responsiveness |
| TaDUF966-9B | + | *Arabidopsis thaliana* | cis-acting element involved in the abscisic acid responsiveness |
| TaDUF966-9B | + | *Arabidopsis thaliana* | cis-acting element involved in the abscisic acid responsiveness |
| TaDUF966-9B | + | *Triticum aestivum* | cis-acting element involved in the abscisic acid responsiveness |
| TaDUF966-9B | - | *Triticum aestivum* | cis-acting element involved in the abscisic acid responsiveness |
| TaDUF966-9B | - | *Arabidopsis thaliana* | cis-acting element involved in the abscisic acid responsiveness |
| TaDUF966-9B | + | *Arabidopsis thaliana* | cis-acting element involved in the abscisic acid responsiveness |
| TaDUF966-9B | - | *Zea mays* | cis-acting regulatory element essential for the anaerobic induction |
| TaDUF966-9B | + | *Zea mays* | cis-acting regulatory element essential for the anaerobic induction |
| TaDUF966-9B | + | *Zea mays* | cis-acting regulatory element essential for the anaerobic induction |
| TaDUF966-9B | - | *Pisum sativum* | common cis-acting element in promoter and enhancer regions |
| TaDUF966-9B | - | *Nicotiana glutinosa* |  |
| TaDUF966-9B | + | *Arabidopsis thaliana* | common cis-acting element in promoter and enhancer regions |
| TaDUF966-9B | + | *Nicotiana glutinosa* |  |
| TaDUF966-9B | + | *Nicotiana glutinosa* |  |
| TaDUF966-9B | - | *Arabidopsis thaliana* | cis-acting regulatory element related to meristem expression |
| TaDUF966-9B | + | *Arabidopsis thaliana* | cis-acting regulatory element related to meristem expression |
| TaDUF966-9B | + | *Hordeum vulgare* | MYBHv1 binding site |
| TaDUF966-9B | + | *Hordeum vulgare* | cis-acting regulatory element involved in the MeJA-responsiveness |
| TaDUF966-9B | - | *Pisum sativum* | cis-acting regulatory element involved in light responsiveness |
| TaDUF966-9B | - | *Pisum sativum* | cis-acting regulatory element involved in light responsiveness |
| TaDUF966-9B | + | *Pisum sativum* | cis-acting regulatory element involved in light responsiveness |
| TaDUF966-9B | - | *Pisum sativum* | cis-acting regulatory element involved in light responsiveness |
| TaDUF966-9B | - | *Zea mays* | cis-acting regulatory element involved in light responsiveness |
| TaDUF966-9B | - | *Hordeum vulgare* | cis-acting element involved in low-temperature responsiveness |
| TaDUF966-9B | - | *Hordeum vulgare* | cis-acting element involved in low-temperature responsiveness |
| TaDUF966-9B | - | *Hordeum vulgare* | cis-acting element involved in low-temperature responsiveness |
| TaDUF966-9B | - | *Arabidopsis thaliana* | MYB binding site involved in drought-inducibility |
| TaDUF966-9B | + | *Arabidopsis thaliana* | MYB binding site involved in drought-inducibility |
| TaDUF966-9B | + | *Arabidopsis thaliana* | MYB binding site involved in drought-inducibility |
| TaDUF966-9B | - | *Arabidopsis thaliana* | core promoter element around -30 of transcription start |
| TaDUF966-9B | - | *Arabidopsis thaliana* | core promoter element around -30 of transcription start |
| TaDUF966-9B | - | *Brassica napus* | core promoter element around -30 of transcription start |
| TaDUF966-9B | - | *Arabidopsis thaliana* | core promoter element around -30 of transcription start |
| TaDUF966-9B | - | *Arabidopsis thaliana* | core promoter element around -30 of transcription start |
| TaDUF966-9B | - | *Hordeum vulgare* | cis-acting regulatory element involved in the MeJA-responsiveness |
| TaDUF966-9D | + | *Hordeum vulgare* | cis-acting element involved in the abscisic acid responsiveness |
| TaDUF966-9D | + | *Arabidopsis thaliana* | cis-acting element involved in the abscisic acid responsiveness |
| TaDUF966-9D | + | *Arabidopsis thaliana* | cis-acting element involved in the abscisic acid responsiveness |
| TaDUF966-9D | + | *Arabidopsis thaliana* | cis-acting element involved in the abscisic acid responsiveness |
| TaDUF966-9D | - | *Arabidopsis thaliana* | cis-acting element involved in the abscisic acid responsiveness |
| TaDUF966-9D | + | *Arabidopsis thaliana* | cis-acting element involved in the abscisic acid responsiveness |
| TaDUF966-9D | - | *Arabidopsis thaliana* | cis-acting element involved in the abscisic acid responsiveness |
| TaDUF966-9D | - | *Zea mays* | cis-acting regulatory element essential for the anaerobic induction |
| TaDUF966-9D | + | *Pisum sativum* | common cis-acting element in promoter and enhancer regions |
| TaDUF966-9D | + | *Nicotiana glutinosa* |  |
| TaDUF966-9D | - | *Nicotiana glutinosa* |  |
| TaDUF966-9D | - | *Pisum sativum* | common cis-acting element in promoter and enhancer regions |
| TaDUF966-9D | - | *Nicotiana glutinosa* |  |
| TaDUF966-9D | - | *Nicotiana glutinosa* |  |
| TaDUF966-9D | - | *Nicotiana glutinosa* |  |
| TaDUF966-9D | + | *Nicotiana glutinosa* |  |
| TaDUF966-9D | - | *Nicotiana glutinosa* |  |
| TaDUF966-9D | - | *Pisum sativum* | common cis-acting element in promoter and enhancer regions |
| TaDUF966-9D | + | *Pisum sativum* | common cis-acting element in promoter and enhancer regions |
| TaDUF966-9D | + | *Pisum sativum* | common cis-acting element in promoter and enhancer regions |
| TaDUF966-9D | - | *Nicotiana glutinosa* |  |
| TaDUF966-9D | + | *Nicotiana glutinosa* |  |
| TaDUF966-9D | + | *Hordeum vulgare* | MYBHv1 binding site |
| TaDUF966-9D | - | *Hordeum vulgare* | cis-acting regulatory element involved in the MeJA-responsiveness |
| TaDUF966-9D | + | *Pisum sativum* | cis-acting regulatory element involved in light responsiveness |
| TaDUF966-9D | - | *Pisum sativum* | cis-acting regulatory element involved in light responsiveness |
| TaDUF966-9D | - | *Pisum sativum* | cis-acting regulatory element involved in light responsiveness |
| TaDUF966-9D | + | *Brassica napus* | cis-acting regulatory element involved in light responsiveness |
| TaDUF966-9D | + | *Arabidopsis thaliana* | cis-acting regulatory element involved in light responsiveness |
| TaDUF966-9D | - | *Arabidopsis thaliana* | cis-acting regulatory element involved in light responsiveness |
| TaDUF966-9D | - | *Arabidopsis thaliana* | cis-acting regulatory element involved in light responsiveness |
| TaDUF966-9D | - | *Arabidopsis thaliana* | light responsive element |
| TaDUF966-9D | + | *Arabidopsis thaliana* | MYB binding site involved in drought-inducibility |
| TaDUF966-9D | + | *Arabidopsis thaliana* | MYB binding site involved in drought-inducibility |
| TaDUF966-9D | - | *Pisum sativum* | core promoter element around -30 of transcription start |
| TaDUF966-9D | - | *Helianthus annuus* | core promoter element around -30 of transcription start |
| TaDUF966-9D | - | *Arabidopsis thaliana* | core promoter element around -30 of transcription start |
| TaDUF966-9D | + | *Arabidopsis thaliana* | core promoter element around -30 of transcription start |
| TaDUF966-9D | + | *Brassica napus* | core promoter element around -30 of transcription start |
| TaDUF966-9D | - | *Arabidopsis thaliana* | core promoter element around -30 of transcription start |
| TaDUF966-9D | + | *Arabidopsis thaliana* | core promoter element around -30 of transcription start |
| TaDUF966-9D | + | *Brassica napus* | core promoter element around -30 of transcription start |
| TaDUF966-9D | - | *Arabidopsis thaliana* | core promoter element around -30 of transcription start |
| TaDUF966-9D | + | *Arabidopsis thaliana* | core promoter element around -30 of transcription start |
| TaDUF966-9D | + | *Brassica napus* | core promoter element around -30 of transcription start |
| TaDUF966-9D | - | *Arabidopsis thaliana* | core promoter element around -30 of transcription start |
| TaDUF966-9D | + | *Arabidopsis thaliana* | core promoter element around -30 of transcription start |
| TaDUF966-9D | + | *Brassica napus* | core promoter element around -30 of transcription start |
| TaDUF966-9D | - | *Arabidopsis thaliana* | core promoter element around -30 of transcription start |
| TaDUF966-9D | - | *Arabidopsis thaliana* | core promoter element around -30 of transcription start |
| TaDUF966-9D | + | *Oryza sativa* | core promoter element around -30 of transcription start |
| TaDUF966-9D | + | *Brassica oleracea* | core promoter element around -30 of transcription start |
| TaDUF966-9D | - | *Arabidopsis thaliana* | core promoter element around -30 of transcription start |
| TaDUF966-9D | - | *Arabidopsis thaliana* | core promoter element around -30 of transcription start |
| TaDUF966-9D | + | *Arabidopsis thaliana* | core promoter element around -30 of transcription start |
| TaDUF966-9D | + | *Brassica napus* | core promoter element around -30 of transcription start |
| TaDUF966-9D | - | *Arabidopsis thaliana* | core promoter element around -30 of transcription start |
| TaDUF966-9D | - | *Arabidopsis thaliana* | core promoter element around -30 of transcription start |
| TaDUF966-9D | - | *Arabidopsis thaliana* | core promoter element around -30 of transcription start |
| TaDUF966-9D | - | *Brassica napus* | core promoter element around -30 of transcription start |
| TaDUF966-9D | - | *Arabidopsis thaliana* | core promoter element around -30 of transcription start |
| TaDUF966-9D | + | *Brassica oleracea* | core promoter element around -30 of transcription start |
| TaDUF966-9D | - | *Arabidopsis thaliana* | core promoter element around -30 of transcription start |
| TaDUF966-9D | + | *Hordeum vulgare* | cis-acting regulatory element involved in the MeJA-responsiveness |
| TaDUF966-9D | + | *Brassica oleracea* | auxin-responsive element |
| TaDUF966-10A | + | *Arabidopsis thaliana* | cis-acting element involved in the abscisic acid responsiveness |
| TaDUF966-10A | + | *Arabidopsis thaliana* | cis-acting element involved in the abscisic acid responsiveness |
| TaDUF966-10A | + | *Arabidopsis thaliana* | cis-acting element involved in the abscisic acid responsiveness |
| TaDUF966-10A | - | *Hordeum vulgare* | cis-acting element involved in the abscisic acid responsiveness |
| TaDUF966-10A | - | *Arabidopsis thaliana* | cis-acting element involved in the abscisic acid responsiveness |
| TaDUF966-10A | - | *Nicotiana glutinosa* |  |
| TaDUF966-10A | + | *Nicotiana glutinosa* |  |
| TaDUF966-10A | - | *Pisum sativum* | common cis-acting element in promoter and enhancer regions |
| TaDUF966-10A | + | *Pisum sativum* | common cis-acting element in promoter and enhancer regions |
| TaDUF966-10A | + | *Pisum sativum* | common cis-acting element in promoter and enhancer regions |
| TaDUF966-10A | - | *Nicotiana glutinosa* |  |
| TaDUF966-10A | + | *Nicotiana glutinosa* |  |
| TaDUF966-10A | - | *Nicotiana glutinosa* |  |
| TaDUF966-10A | + | *Nicotiana glutinosa* |  |
| TaDUF966-10A | + | *Arabidopsis thaliana* | common cis-acting element in promoter and enhancer regions |
| TaDUF966-10A | + | *Nicotiana glutinosa* |  |
| TaDUF966-10A | + | *Arabidopsis thaliana* | common cis-acting element in promoter and enhancer regions |
| TaDUF966-10A | + | *Nicotiana glutinosa* |  |
| TaDUF966-10A | + | *Arabidopsis thaliana* | common cis-acting element in promoter and enhancer regions |
| TaDUF966-10A | - | *Pisum sativum* | common cis-acting element in promoter and enhancer regions |
| TaDUF966-10A | - | *Nicotiana glutinosa* |  |
| TaDUF966-10A | + | *Nicotiana glutinosa* |  |
| TaDUF966-10A | + | *Nicotiana glutinosa* |  |
| TaDUF966-10A | - | *Pisum sativum* | common cis-acting element in promoter and enhancer regions |
| TaDUF966-10A | + | *Pisum sativum* | common cis-acting element in promoter and enhancer regions |
| TaDUF966-10A | - | *Arabidopsis thaliana* | cis-acting regulatory element related to meristem expression |
| TaDUF966-10A | - | *Arabidopsis thaliana* | cis-acting regulatory element related to meristem expression |
| TaDUF966-10A | - | *Hordeum vulgare* | MYBHv1 binding site |
| TaDUF966-10A | - | *Hordeum vulgare* | MYBHv1 binding site |
| TaDUF966-10A | + | *Hordeum vulgare* | cis-acting regulatory element involved in the MeJA-responsiveness |
| TaDUF966-10A | + | *Arabidopsis thaliana* | part of a light responsive element |
| TaDUF966-10A | + | *Pisum sativum* | cis-acting regulatory element involved in light responsiveness |
| TaDUF966-10A | + | *Arabidopsis thaliana* | cis-acting regulatory element involved in light responsiveness |
| TaDUF966-10A | + | *Arabidopsis thaliana* | cis-acting regulatory element involved in light responsiveness |
| TaDUF966-10A | - | *Arabidopsis thaliana* | cis-acting regulatory element involved in light responsiveness |
| TaDUF966-10A | - | *Gossypium hirsutum* | part of a light responsive element |
| TaDUF966-10A | - | *Triticum aestivum* | part of a light responsive element |
| TaDUF966-10A | + | *Zea mays* | part of a light responsive element |
| TaDUF966-10A | - | *Hordeum vulgare* | cis-acting element involved in low-temperature responsiveness |
| TaDUF966-10A | + | *Oryza sativa* | gibberellin-responsive element |
| TaDUF966-10A | - | *Arabidopsis thaliana* | core promoter element around -30 of transcription start |
| TaDUF966-10A | - | *Hordeum vulgare* | cis-acting regulatory element involved in the MeJA-responsiveness |
| TaDUF966-10B | - | *Petroselinum crispum* | cis-acting regulatory element |
| TaDUF966-10B | + | *Arabidopsis thaliana* | cis-acting element involved in the abscisic acid responsiveness |
| TaDUF966-10B | + | *Arabidopsis thaliana* | cis-acting element involved in the abscisic acid responsiveness |
| TaDUF966-10B | - | *Arabidopsis thaliana* | part of a module for light response |
| TaDUF966-10B | + | *Zea mays* | cis-acting regulatory element essential for the anaerobic induction |
| TaDUF966-10B | - | *Zea mays* | cis-acting regulatory element essential for the anaerobic induction |
| TaDUF966-10B | - | *Nicotiana glutinosa* |  |
| TaDUF966-10B | - | *Arabidopsis thaliana* | common cis-acting element in promoter and enhancer regions |
| TaDUF966-10B | - | *Arabidopsis thaliana* | common cis-acting element in promoter and enhancer regions |
| TaDUF966-10B | - | *Arabidopsis thaliana* | common cis-acting element in promoter and enhancer regions |
| TaDUF966-10B | + | *Arabidopsis thaliana* | common cis-acting element in promoter and enhancer regions |
| TaDUF966-10B | + | *Nicotiana glutinosa* |  |
| TaDUF966-10B | + | *Arabidopsis thaliana* | common cis-acting element in promoter and enhancer regions |
| TaDUF966-10B | - | *Nicotiana glutinosa* |  |
| TaDUF966-10B | - | *Pisum sativum* | common cis-acting element in promoter and enhancer regions |
| TaDUF966-10B | - | *Nicotiana glutinosa* |  |
| TaDUF966-10B | + | *Nicotiana glutinosa* |  |
| TaDUF966-10B | + | *Arabidopsis thaliana* | common cis-acting element in promoter and enhancer regions |
| TaDUF966-10B | + | *Nicotiana glutinosa* |  |
| TaDUF966-10B | + | *Nicotiana glutinosa* |  |
| TaDUF966-10B | + | *Pisum sativum* | common cis-acting element in promoter and enhancer regions |
| TaDUF966-10B | - | *Nicotiana glutinosa* |  |
| TaDUF966-10B | + | *Nicotiana glutinosa* |  |
| TaDUF966-10B | - | *Nicotiana glutinosa* |  |
| TaDUF966-10B | - | *Nicotiana glutinosa* |  |
| TaDUF966-10B | + | *Pisum sativum* | common cis-acting element in promoter and enhancer regions |
| TaDUF966-10B | - | *Nicotiana glutinosa* |  |
| TaDUF966-10B | + | *Pisum sativum* | common cis-acting element in promoter and enhancer regions |
| TaDUF966-10B | + | *Arabidopsis thaliana* | cis-acting regulatory element related to meristem expression |
| TaDUF966-10B | - | *Hordeum vulgare* | cis-acting regulatory element involved in the MeJA-responsiveness |
| TaDUF966-10B | + | *Hordeum vulgare* | cis-acting regulatory element involved in the MeJA-responsiveness |
| TaDUF966-10B | - | *Hordeum vulgare* | cis-acting regulatory element involved in the MeJA-responsiveness |
| TaDUF966-10B | + | *Arabidopsis thaliana* | part of a light responsive element |
| TaDUF966-10B | + | *Arabidopsis thaliana* | part of a light responsive element |
| TaDUF966-10B | + | *Arabidopsis thaliana* | cis-acting regulatory element involved in light responsiveness |
| TaDUF966-10B | + | *Pisum sativum* | cis-acting regulatory element involved in light responsiveness |
| TaDUF966-10B | + | *Triticum aestivum* | cis-acting regulatory element involved in light responsiveness |
| TaDUF966-10B | - | *Avena sativa* | light responsive element |
| TaDUF966-10B | - | *Arabidopsis thaliana* | light responsive element |
| TaDUF966-10B | - | *Oryza sativa* | light responsive element |
| TaDUF966-10B | + | *Oryza sativa* | light responsive element |
| TaDUF966-10B | + | *Brassica napus* | core promoter element around -30 of transcription start |
| TaDUF966-10B | + | *Arabidopsis thaliana* | core promoter element around -30 of transcription start |
| TaDUF966-10B | + | *Oryza sativa* | core promoter element around -30 of transcription start |
| TaDUF966-10B | - | *Arabidopsis thaliana* | core promoter element around -30 of transcription start |
| TaDUF966-10B | + | *Arabidopsis thaliana* | core promoter element around -30 of transcription start |
| TaDUF966-10B | + | *Arabidopsis thaliana* | core promoter element around -30 of transcription start |
| TaDUF966-10B | + | *Oryza sativa* | core promoter element around -30 of transcription start |
| TaDUF966-10B | - | *Brassica napus* | core promoter element around -30 of transcription start |
| TaDUF966-10B | - | *Arabidopsis thaliana* | core promoter element around -30 of transcription start |
| TaDUF966-10B | + | *Brassica oleracea* | cis-acting element involved in salicylic acid responsiveness |
| TaDUF966-10B | + | *Hordeum vulgare* | cis-acting regulatory element involved in the MeJA-responsiveness |
| TaDUF966-10B | - | *Hordeum vulgare* | cis-acting regulatory element involved in the MeJA-responsiveness |
| TaDUF966-10B | + | *Hordeum vulgare* | cis-acting regulatory element involved in the MeJA-responsiveness |
| TaDUF966-10D | + | *Hordeum vulgare* | cis-acting element involved in the abscisic acid responsiveness |
| TaDUF966-10D | + | *Arabidopsis thaliana* | cis-acting element involved in the abscisic acid responsiveness |
| TaDUF966-10D | + | *Arabidopsis thaliana* | cis-acting element involved in the abscisic acid responsiveness |
| TaDUF966-10D | + | *Zea mays* | cis-acting regulatory element essential for the anaerobic induction |
| TaDUF966-10D | - | *Petroselinum crispum* | part of a conserved DNA module involved in light responsiveness |
| TaDUF966-10D | + | *Nicotiana glutinosa* |  |
| TaDUF966-10D | + | *Pisum sativum* | common cis-acting element in promoter and enhancer regions |
| TaDUF966-10D | - | *Nicotiana glutinosa* |  |
| TaDUF966-10D | + | *Nicotiana glutinosa* |  |
| TaDUF966-10D | + | *Pisum sativum* | common cis-acting element in promoter and enhancer regions |
| TaDUF966-10D | - | *Nicotiana glutinosa* |  |
| TaDUF966-10D | + | *Nicotiana glutinosa* |  |
| TaDUF966-10D | - | *Nicotiana glutinosa* |  |
| TaDUF966-10D | - | *Pisum sativum* | common cis-acting element in promoter and enhancer regions |
| TaDUF966-10D | + | *Nicotiana glutinosa* |  |
| TaDUF966-10D | - | *Nicotiana glutinosa* |  |
| TaDUF966-10D | - | *Nicotiana glutinosa* |  |
| TaDUF966-10D | - | *Arabidopsis thaliana* | common cis-acting element in promoter and enhancer regions |
| TaDUF966-10D | - | *Arabidopsis thaliana* | common cis-acting element in promoter and enhancer regions |
| TaDUF966-10D | - | *Pisum sativum* | common cis-acting element in promoter and enhancer regions |
| TaDUF966-10D | - | *Arabidopsis thaliana* | common cis-acting element in promoter and enhancer regions |
| TaDUF966-10D | - | *Nicotiana glutinosa* |  |
| TaDUF966-10D | + | *Pisum sativum* | common cis-acting element in promoter and enhancer regions |
| TaDUF966-10D | + | *Pisum sativum* | common cis-acting element in promoter and enhancer regions |
| TaDUF966-10D | - | *Arabidopsis thaliana* | cis-acting regulatory element related to meristem expression |
| TaDUF966-10D | + | *Arabidopsis thaliana* | cis-acting regulatory element related to meristem expression |
| TaDUF966-10D | + | *Hordeum vulgare* | cis-acting regulatory element involved in the MeJA-responsiveness |
| TaDUF966-10D | + | *Zea mays* | cis-acting regulatory element involved in light responsiveness |
| TaDUF966-10D | + | *Arabidopsis thaliana* | cis-acting regulatory element involved in light responsiveness |
| TaDUF966-10D | - | *Zea mays* | cis-acting regulatory element involved in light responsiveness |
| TaDUF966-10D | - | *Arabidopsis thaliana* | light responsive element |
| TaDUF966-10D | + | *Solanum tuberosum* | light responsive element |
| TaDUF966-10D | + | *Zea mays* | part of a light responsive element |
| TaDUF966-10D | + | *Arabidopsis thaliana* | MYB binding site involved in drought-inducibility |
| TaDUF966-10D | + | *Oryza sativa* | gibberellin-responsive element |
| TaDUF966-10D | - | *Oryza sativa* | gibberellin-responsive element |
| TaDUF966-10D | - | *Helianthus annuus* | core promoter element around -30 of transcription start |
| TaDUF966-10D | + | *Arabidopsis thaliana* | core promoter element around -30 of transcription start |
| TaDUF966-10D | + | *Arabidopsis thaliana* | core promoter element around -30 of transcription start |
| TaDUF966-10D | - | *Helianthus annuus* | core promoter element around -30 of transcription start |
| TaDUF966-10D | + | *Arabidopsis thaliana* | core promoter element around -30 of transcription start |
| TaDUF966-10D | - | *Arabidopsis thaliana* | core promoter element around -30 of transcription start |
| TaDUF966-10D | - | *Arabidopsis thaliana* | core promoter element around -30 of transcription start |
| TaDUF966-10D | + | *Arabidopsis thaliana* | core promoter element around -30 of transcription start |
| TaDUF966-10D | - | *Nicotiana tabacum* | cis-acting element involved in salicylic acid responsiveness |
| TaDUF966-10D | - | *Hordeum vulgare* | cis-acting regulatory element involved in the MeJA-responsiveness |
| TaDUF966-11B | + | *Arabidopsis thaliana* | part of a module for light response |
| TaDUF966-11B | + | *Zea mays* | cis-acting regulatory element essential for the anaerobic induction |
| TaDUF966-11B | + | *Nicotiana glutinosa* |  |
| TaDUF966-11B | - | *Pisum sativum* | common cis-acting element in promoter and enhancer regions |
| TaDUF966-11B | - | *Nicotiana glutinosa* |  |
| TaDUF966-11B | - | *Nicotiana glutinosa* |  |
| TaDUF966-11B | + | *Arabidopsis thaliana* | common cis-acting element in promoter and enhancer regions |
| TaDUF966-11B | + | *Nicotiana glutinosa* |  |
| TaDUF966-11B | + | *Pisum sativum* | common cis-acting element in promoter and enhancer regions |
| TaDUF966-11B | + | *Arabidopsis thaliana* | common cis-acting element in promoter and enhancer regions |
| TaDUF966-11B | + | *Nicotiana glutinosa* |  |
| TaDUF966-11B | + | *Pisum sativum* | common cis-acting element in promoter and enhancer regions |
| TaDUF966-11B | + | *Arabidopsis thaliana* | common cis-acting element in promoter and enhancer regions |
| TaDUF966-11B | + | *Nicotiana glutinosa* |  |
| TaDUF966-11B | + | *Nicotiana glutinosa* |  |
| TaDUF966-11B | + | *Pisum sativum* | common cis-acting element in promoter and enhancer regions |
| TaDUF966-11B | - | *Nicotiana glutinosa* |  |
| TaDUF966-11B | - | *Nicotiana glutinosa* |  |
| TaDUF966-11B | + | *Arabidopsis thaliana* | common cis-acting element in promoter and enhancer regions |
| TaDUF966-11B | + | *Nicotiana glutinosa* |  |
| TaDUF966-11B | - | *Pisum sativum* | common cis-acting element in promoter and enhancer regions |
| TaDUF966-11B | + | *Nicotiana glutinosa* |  |
| TaDUF966-11B | + | *Nicotiana glutinosa* |  |
| TaDUF966-11B | - | *Nicotiana glutinosa* |  |
| TaDUF966-11B | + | *Arabidopsis thaliana* | common cis-acting element in promoter and enhancer regions |
| TaDUF966-11B | + | *Nicotiana glutinosa* |  |
| TaDUF966-11B | - | *Nicotiana glutinosa* |  |
| TaDUF966-11B | + | *Nicotiana glutinosa* |  |
| TaDUF966-11B | + | *Nicotiana glutinosa* |  |
| TaDUF966-11B | + | *Nicotiana glutinosa* |  |
| TaDUF966-11B | - | *Nicotiana glutinosa* |  |
| TaDUF966-11B | + | *Nicotiana glutinosa* |  |
| TaDUF966-11B | - | *Arabidopsis thaliana* | common cis-acting element in promoter and enhancer regions |
| TaDUF966-11B | + | *Arabidopsis thaliana* | common cis-acting element in promoter and enhancer regions |
| TaDUF966-11B | + | *Nicotiana glutinosa* |  |
| TaDUF966-11B | - | *Arabidopsis thaliana* | cis-acting regulatory element related to meristem expression |
| TaDUF966-11B | - | *Hordeum vulgare* | cis-acting regulatory element involved in the MeJA-responsiveness |
| TaDUF966-11B | - | *Hordeum vulgare* | cis-acting regulatory element involved in the MeJA-responsiveness |
| TaDUF966-11B | + | *Arabidopsis thaliana* | part of a light responsive element |
| TaDUF966-11B | + | *Arabidopsis thaliana* | part of a light responsive element |
| TaDUF966-11B | + | *Pisum sativum* | part of a light responsive element |
| TaDUF966-11B | + | *Zea mays* | cis-acting regulatory element involved in light responsiveness |
| TaDUF966-11B | - | *Arabidopsis thaliana* | light responsive element |
| TaDUF966-11B | + | *Arabidopsis thaliana* | MYB binding site involved in drought-inducibility |
| TaDUF966-11B | + | *Arabidopsis thaliana* | MYB binding site involved in drought-inducibility |
| TaDUF966-11B | - | *Oryza sativa* | gibberellin-responsive element |
| TaDUF966-11B | + | *Arabidopsis thaliana* | core promoter element around -30 of transcription start |
| TaDUF966-11B | - | *Helianthus annuus* | core promoter element around -30 of transcription start |
| TaDUF966-11B | + | *Arabidopsis thaliana* | core promoter element around -30 of transcription start |
| TaDUF966-11B | + | *Arabidopsis thaliana* | core promoter element around -30 of transcription start |
| TaDUF966-11B | - | *Helianthus annuus* | core promoter element around -30 of transcription start |
| TaDUF966-11B | + | *Arabidopsis thaliana* | core promoter element around -30 of transcription start |
| TaDUF966-11B | - | *Helianthus annuus* | core promoter element around -30 of transcription start |
| TaDUF966-11B | + | *Arabidopsis thaliana* | core promoter element around -30 of transcription start |
| TaDUF966-11B | + | *Brassica napus* | core promoter element around -30 of transcription start |
| TaDUF966-11B | - | *Arabidopsis thaliana* | core promoter element around -30 of transcription start |
| TaDUF966-11B | + | *Arabidopsis thaliana* | core promoter element around -30 of transcription start |
| TaDUF966-11B | + | *Arabidopsis thaliana* | core promoter element around -30 of transcription start |
| TaDUF966-11B | - | *Arabidopsis thaliana* | core promoter element around -30 of transcription start |
| TaDUF966-11B | + | *Arabidopsis thaliana* | core promoter element around -30 of transcription start |
| TaDUF966-11B | + | *Oryza sativa* | core promoter element around -30 of transcription start |
| TaDUF966-11B | - | *Helianthus annuus* | core promoter element around -30 of transcription start |
| TaDUF966-11B | + | *Arabidopsis thaliana* | core promoter element around -30 of transcription start |
| TaDUF966-11B | + | *Arabidopsis thaliana* | core promoter element around -30 of transcription start |
| TaDUF966-11B | + | *Arabidopsis thaliana* | core promoter element around -30 of transcription start |
| TaDUF966-11B | + | *Arabidopsis thaliana* | core promoter element around -30 of transcription start |
| TaDUF966-11B | + | *Brassica napus* | core promoter element around -30 of transcription start |
| TaDUF966-11B | - | *Arabidopsis thaliana* | core promoter element around -30 of transcription start |
| TaDUF966-11B | - | *Arabidopsis thaliana* | core promoter element around -30 of transcription start |
| TaDUF966-11B | - | *Helianthus annuus* | core promoter element around -30 of transcription start |
| TaDUF966-11B | - | *Arabidopsis thaliana* | core promoter element around -30 of transcription start |
| TaDUF966-11B | - | *Arabidopsis thaliana* | core promoter element around -30 of transcription start |
| TaDUF966-11B | - | *Arabidopsis thaliana* | core promoter element around -30 of transcription start |
| TaDUF966-11B | + | *Hordeum vulgare* | cis-acting regulatory element involved in the MeJA-responsiveness |
| TaDUF966-11B | + | *Hordeum vulgare* | cis-acting regulatory element involved in the MeJA-responsiveness |
| TaDUF966-12B | + | *Arabidopsis thaliana* | part of a module for light response |
| TaDUF966-12B | + | *Zea mays* | cis-acting regulatory element essential for the anaerobic induction |
| TaDUF966-12B | + | *Nicotiana glutinosa* |  |
| TaDUF966-12B | - | *Pisum sativum* | common cis-acting element in promoter and enhancer regions |
| TaDUF966-12B | - | *Nicotiana glutinosa* |  |
| TaDUF966-12B | - | *Nicotiana glutinosa* |  |
| TaDUF966-12B | + | *Arabidopsis thaliana* | common cis-acting element in promoter and enhancer regions |
| TaDUF966-12B | + | *Nicotiana glutinosa* |  |
| TaDUF966-12B | + | *Pisum sativum* | common cis-acting element in promoter and enhancer regions |
| TaDUF966-12B | + | *Arabidopsis thaliana* | common cis-acting element in promoter and enhancer regions |
| TaDUF966-12B | + | *Nicotiana glutinosa* |  |
| TaDUF966-12B | + | *Pisum sativum* | common cis-acting element in promoter and enhancer regions |
| TaDUF966-12B | + | *Nicotiana glutinosa* |  |
| TaDUF966-12B | + | *Pisum sativum* | common cis-acting element in promoter and enhancer regions |
| TaDUF966-12B | - | *Nicotiana glutinosa* |  |
| TaDUF966-12B | - | *Nicotiana glutinosa* |  |
| TaDUF966-12B | + | *Arabidopsis thaliana* | common cis-acting element in promoter and enhancer regions |
| TaDUF966-12B | + | *Nicotiana glutinosa* |  |
| TaDUF966-12B | - | *Pisum sativum* | common cis-acting element in promoter and enhancer regions |
| TaDUF966-12B | - | *Nicotiana glutinosa* |  |
| TaDUF966-12B | + | *Nicotiana glutinosa* |  |
| TaDUF966-12B | + | *Nicotiana glutinosa* |  |
| TaDUF966-12B | - | *Nicotiana glutinosa* |  |
| TaDUF966-12B | + | *Arabidopsis thaliana* | common cis-acting element in promoter and enhancer regions |
| TaDUF966-12B | + | *Nicotiana glutinosa* |  |
| TaDUF966-12B | - | *Nicotiana glutinosa* |  |
| TaDUF966-12B | + | *Nicotiana glutinosa* |  |
| TaDUF966-12B | + | *Nicotiana glutinosa* |  |
| TaDUF966-12B | + | *Nicotiana glutinosa* |  |
| TaDUF966-12B | - | *Nicotiana glutinosa* |  |
| TaDUF966-12B | + | *Nicotiana glutinosa* |  |
| TaDUF966-12B | - | *Arabidopsis thaliana* | common cis-acting element in promoter and enhancer regions |
| TaDUF966-12B | + | *Arabidopsis thaliana* | common cis-acting element in promoter and enhancer regions |
| TaDUF966-12B | + | *Nicotiana glutinosa* |  |
| TaDUF966-12B | - | *Arabidopsis thaliana* | cis-acting regulatory element related to meristem expression |
| TaDUF966-12B | - | *Hordeum vulgare* | cis-acting regulatory element involved in the MeJA-responsiveness |
| TaDUF966-12B | + | *Arabidopsis thaliana* | part of a light responsive element |
| TaDUF966-12B | + | *Arabidopsis thaliana* | part of a light responsive element |
| TaDUF966-12B | + | *Pisum sativum* | part of a light responsive element |
| TaDUF966-12B | + | *Zea mays* | cis-acting regulatory element involved in light responsiveness |
| TaDUF966-12B | - | *Arabidopsis thaliana* | light responsive element |
| TaDUF966-12B | + | *Arabidopsis thaliana* | MYB binding site involved in drought-inducibility |
| TaDUF966-12B | - | *Oryza sativa* | gibberellin-responsive element |
| TaDUF966-12B | + | *Brassica oleracea* | core promoter element around -30 of transcription start |
| TaDUF966-12B | + | *Arabidopsis thaliana* | core promoter element around -30 of transcription start |
| TaDUF966-12B | + | *Arabidopsis thaliana* | core promoter element around -30 of transcription start |
| TaDUF966-12B | - | *Helianthus annuus* | core promoter element around -30 of transcription start |
| TaDUF966-12B | + | *Arabidopsis thaliana* | core promoter element around -30 of transcription start |
| TaDUF966-12B | + | *Arabidopsis thaliana* | core promoter element around -30 of transcription start |
| TaDUF966-12B | - | *Helianthus annuus* | core promoter element around -30 of transcription start |
| TaDUF966-12B | + | *Arabidopsis thaliana* | core promoter element around -30 of transcription start |
| TaDUF966-12B | + | *Arabidopsis thaliana* | core promoter element around -30 of transcription start |
| TaDUF966-12B | + | *Arabidopsis thaliana* | core promoter element around -30 of transcription start |
| TaDUF966-12B | + | *Brassica napus* | core promoter element around -30 of transcription start |
| TaDUF966-12B | - | *Arabidopsis thaliana* | core promoter element around -30 of transcription start |
| TaDUF966-12B | + | *Arabidopsis thaliana* | core promoter element around -30 of transcription start |
| TaDUF966-12B | + | *Arabidopsis thaliana* | core promoter element around -30 of transcription start |
| TaDUF966-12B | - | *Arabidopsis thaliana* | core promoter element around -30 of transcription start |
| TaDUF966-12B | + | *Arabidopsis thaliana* | core promoter element around -30 of transcription start |
| TaDUF966-12B | + | *Oryza sativa* | core promoter element around -30 of transcription start |
| TaDUF966-12B | - | *Helianthus annuus* | core promoter element around -30 of transcription start |
| TaDUF966-12B | + | *Arabidopsis thaliana* | core promoter element around -30 of transcription start |
| TaDUF966-12B | + | *Arabidopsis thaliana* | core promoter element around -30 of transcription start |
| TaDUF966-12B | + | *Arabidopsis thaliana* | core promoter element around -30 of transcription start |
| TaDUF966-12B | + | *Arabidopsis thaliana* | core promoter element around -30 of transcription start |
| TaDUF966-12B | + | *Brassica napus* | core promoter element around -30 of transcription start |
| TaDUF966-12B | - | *Arabidopsis thaliana* | core promoter element around -30 of transcription start |
| TaDUF966-12B | - | *Arabidopsis thaliana* | core promoter element around -30 of transcription start |
| TaDUF966-12B | - | *Helianthus annuus* | core promoter element around -30 of transcription start |
| TaDUF966-12B | - | *Arabidopsis thaliana* | core promoter element around -30 of transcription start |
| TaDUF966-12B | - | *Arabidopsis thaliana* | core promoter element around -30 of transcription start |
| TaDUF966-12B | - | *Arabidopsis thaliana* | core promoter element around -30 of transcription start |
| TaDUF966-12B | + | *Hordeum vulgare* | cis-acting regulatory element involved in the MeJA-responsiveness |
| TaDUF966-13A | + | *Arabidopsis thaliana* | part of a module for light response |
| TaDUF966-13A | + | *Zea mays* | cis-acting regulatory element essential for the anaerobic induction |
| TaDUF966-13A | + | *Pisum sativum* | common cis-acting element in promoter and enhancer regions |
| TaDUF966-13A | + | *Nicotiana glutinosa* |  |
| TaDUF966-13A | - | *Pisum sativum* | common cis-acting element in promoter and enhancer regions |
| TaDUF966-13A | - | *Nicotiana glutinosa* |  |
| TaDUF966-13A | - | *Nicotiana glutinosa* |  |
| TaDUF966-13A | + | *Arabidopsis thaliana* | common cis-acting element in promoter and enhancer regions |
| TaDUF966-13A | + | *Nicotiana glutinosa* |  |
| TaDUF966-13A | + | *Pisum sativum* | common cis-acting element in promoter and enhancer regions |
| TaDUF966-13A | + | *Arabidopsis thaliana* | common cis-acting element in promoter and enhancer regions |
| TaDUF966-13A | + | *Nicotiana glutinosa* |  |
| TaDUF966-13A | + | *Pisum sativum* | common cis-acting element in promoter and enhancer regions |
| TaDUF966-13A | + | *Arabidopsis thaliana* | common cis-acting element in promoter and enhancer regions |
| TaDUF966-13A | + | *Nicotiana glutinosa* |  |
| TaDUF966-13A | + | *Nicotiana glutinosa* |  |
| TaDUF966-13A | - | *Nicotiana glutinosa* |  |
| TaDUF966-13A | - | *Nicotiana glutinosa* |  |
| TaDUF966-13A | - | *Arabidopsis thaliana* | common cis-acting element in promoter and enhancer regions |
| TaDUF966-13A | + | *Arabidopsis thaliana* | common cis-acting element in promoter and enhancer regions |
| TaDUF966-13A | + | *Nicotiana glutinosa* |  |
| TaDUF966-13A | - | *Pisum sativum* | common cis-acting element in promoter and enhancer regions |
| TaDUF966-13A | - | *Nicotiana glutinosa* |  |
| TaDUF966-13A | + | *Nicotiana glutinosa* |  |
| TaDUF966-13A | + | *Nicotiana glutinosa* |  |
| TaDUF966-13A | - | *Nicotiana glutinosa* |  |
| TaDUF966-13A | + | *Arabidopsis thaliana* | common cis-acting element in promoter and enhancer regions |
| TaDUF966-13A | + | *Nicotiana glutinosa* |  |
| TaDUF966-13A | - | *Nicotiana glutinosa* |  |
| TaDUF966-13A | + | *Nicotiana glutinosa* |  |
| TaDUF966-13A | + | *Nicotiana glutinosa* |  |
| TaDUF966-13A | + | *Nicotiana glutinosa* |  |
| TaDUF966-13A | - | *Nicotiana glutinosa* |  |
| TaDUF966-13A | + | *Nicotiana glutinosa* |  |
| TaDUF966-13A | - | *Arabidopsis thaliana* | common cis-acting element in promoter and enhancer regions |
| TaDUF966-13A | - | *Arabidopsis thaliana* | cis-acting regulatory element related to meristem expression |
| TaDUF966-13A | - | *Hordeum vulgare* | cis-acting regulatory element involved in the MeJA-responsiveness |
| TaDUF966-13A | + | *Arabidopsis thaliana* | part of a light responsive element |
| TaDUF966-13A | + | *Arabidopsis thaliana* | part of a light responsive element |
| TaDUF966-13A | + | *Pisum sativum* | part of a light responsive element |
| TaDUF966-13A | + | *Zea mays* | cis-acting regulatory element involved in light responsiveness |
| TaDUF966-13A | - | *Arabidopsis thaliana* | light responsive element |
| TaDUF966-13A | + | *Arabidopsis thaliana* | MYB binding site involved in drought-inducibility |
| TaDUF966-13A | + | *Arabidopsis thaliana* | MYB binding site involved in drought-inducibility |
| TaDUF966-13A | + | *Petroselinum crispum* | MYB binding site involved in light responsiveness |
| TaDUF966-13A | - | *Oryza sativa* | gibberellin-responsive element |
| TaDUF966-13A | + | *Brassica oleracea* | core promoter element around -30 of transcription start |
| TaDUF966-13A | + | *Arabidopsis thaliana* | core promoter element around -30 of transcription start |
| TaDUF966-13A | + | *Arabidopsis thaliana* | core promoter element around -30 of transcription start |
| TaDUF966-13A | - | *Helianthus annuus* | core promoter element around -30 of transcription start |
| TaDUF966-13A | + | *Arabidopsis thaliana* | core promoter element around -30 of transcription start |
| TaDUF966-13A | + | *Arabidopsis thaliana* | core promoter element around -30 of transcription start |
| TaDUF966-13A | - | *Helianthus annuus* | core promoter element around -30 of transcription start |
| TaDUF966-13A | + | *Arabidopsis thaliana* | core promoter element around -30 of transcription start |
| TaDUF966-13A | - | *Helianthus annuus* | core promoter element around -30 of transcription start |
| TaDUF966-13A | + | *Arabidopsis thaliana* | core promoter element around -30 of transcription start |
| TaDUF966-13A | + | *Brassica napus* | core promoter element around -30 of transcription start |
| TaDUF966-13A | - | *Arabidopsis thaliana* | core promoter element around -30 of transcription start |
| TaDUF966-13A | + | *Arabidopsis thaliana* | core promoter element around -30 of transcription start |
| TaDUF966-13A | + | *Arabidopsis thaliana* | core promoter element around -30 of transcription start |
| TaDUF966-13A | - | *Arabidopsis thaliana* | core promoter element around -30 of transcription start |
| TaDUF966-13A | + | *Arabidopsis thaliana* | core promoter element around -30 of transcription start |
| TaDUF966-13A | + | *Oryza sativa* | core promoter element around -30 of transcription start |
| TaDUF966-13A | - | *Helianthus annuus* | core promoter element around -30 of transcription start |
| TaDUF966-13A | + | *Arabidopsis thaliana* | core promoter element around -30 of transcription start |
| TaDUF966-13A | + | *Brassica oleracea* | core promoter element around -30 of transcription start |
| TaDUF966-13A | + | *Arabidopsis thaliana* | core promoter element around -30 of transcription start |
| TaDUF966-13A | + | *Arabidopsis thaliana* | core promoter element around -30 of transcription start |
| TaDUF966-13A | + | *Arabidopsis thaliana* | core promoter element around -30 of transcription start |
| TaDUF966-13A | - | *Helianthus annuus* | core promoter element around -30 of transcription start |
| TaDUF966-13A | - | *Arabidopsis thaliana* | core promoter element around -30 of transcription start |
| TaDUF966-13A | - | *Arabidopsis thaliana* | core promoter element around -30 of transcription start |
| TaDUF966-13A | - | *Arabidopsis thaliana* | core promoter element around -30 of transcription start |
| TaDUF966-13A | - | *Arabidopsis thaliana* | core promoter element around -30 of transcription start |
| TaDUF966-13A | - | *Arabidopsis thaliana* | part of a light responsive element |
| TaDUF966-13A | + | *Hordeum vulgare* | cis-acting regulatory element involved in the MeJA-responsiveness |
| TaDUF966-13A | - | *Brassica oleracea* | auxin-responsive element |
| TaDUF966-14A | - | *Petroselinum crispum* | cis-acting regulatory element |
| TaDUF966-14A | - | *Arabidopsis thaliana* | cis-acting element involved in the abscisic acid responsiveness |
| TaDUF966-14A | + | *Arabidopsis thaliana* | cis-acting element involved in the abscisic acid responsiveness |
| TaDUF966-14A | + | *Arabidopsis thaliana* | cis-acting element involved in the abscisic acid responsiveness |
| TaDUF966-14A | - | *Zea mays* | cis-acting regulatory element essential for the anaerobic induction |
| TaDUF966-14A | - | *Petroselinum crispum* | part of a conserved DNA module involved in light responsiveness |
| TaDUF966-14A | + | *Nicotiana glutinosa* |  |
| TaDUF966-14A | - | *Nicotiana glutinosa* |  |
| TaDUF966-14A | + | *Pisum sativum* | common cis-acting element in promoter and enhancer regions |
| TaDUF966-14A | - | *Nicotiana glutinosa* |  |
| TaDUF966-14A | - | *Arabidopsis thaliana* | common cis-acting element in promoter and enhancer regions |
| TaDUF966-14A | + | *Pisum sativum* | common cis-acting element in promoter and enhancer regions |
| TaDUF966-14A | - | *Pisum sativum* | common cis-acting element in promoter and enhancer regions |
| TaDUF966-14A | - | *Pisum sativum* | common cis-acting element in promoter and enhancer regions |
| TaDUF966-14A | + | *Nicotiana glutinosa* |  |
| TaDUF966-14A | - | *Nicotiana glutinosa* |  |
| TaDUF966-14A | + | *Nicotiana glutinosa* |  |
| TaDUF966-14A | + | *Nicotiana glutinosa* |  |
| TaDUF966-14A | - | *Pisum sativum* | common cis-acting element in promoter and enhancer regions |
| TaDUF966-14A | + | *Pisum sativum* | common cis-acting element in promoter and enhancer regions |
| TaDUF966-14A | + | *Nicotiana glutinosa* |  |
| TaDUF966-14A | - | *Nicotiana glutinosa* |  |
| TaDUF966-14A | - | *Nicotiana glutinosa* |  |
| TaDUF966-14A | + | *Pisum sativum* | common cis-acting element in promoter and enhancer regions |
| TaDUF966-14A | + | *Nicotiana glutinosa* |  |
| TaDUF966-14A | + | *Nicotiana glutinosa* |  |
| TaDUF966-14A | + | *Nicotiana glutinosa* |  |
| TaDUF966-14A | + | *Arabidopsis thaliana* | cis-acting regulatory element related to meristem expression |
| TaDUF966-14A | - | *Hordeum vulgare* | MYBHv1 binding site |
| TaDUF966-14A | + | *Hordeum vulgare* | MYBHv1 binding site |
| TaDUF966-14A | + | *Hordeum vulgare* | cis-acting regulatory element involved in the MeJA-responsiveness |
| TaDUF966-14A | - | *Arabidopsis thaliana* | part of a light responsive element |
| TaDUF966-14A | + | *Zea mays* | cis-acting regulatory element involved in light responsiveness |
| TaDUF966-14A | - | *Zea mays* | cis-acting regulatory element involved in light responsiveness |
| TaDUF966-14A | + | *Arabidopsis thaliana* | cis-acting regulatory element involved in light responsiveness |
| TaDUF966-14A | + | *Arabidopsis thaliana* | light responsive element |
| TaDUF966-14A | + | *Hordeum vulgare* | cis-acting element involved in low-temperature responsiveness |
| TaDUF966-14A | - | *Arabidopsis thaliana* | MYB binding site involved in drought-inducibility |
| TaDUF966-14A | + | *Arabidopsis thaliana* | MYB binding site involved in drought-inducibility |
| TaDUF966-14A | - | *Arabidopsis thaliana* | MYB binding site involved in drought-inducibility |
| TaDUF966-14A | - | *Arabidopsis thaliana* | MYB binding site involved in drought-inducibility |
| TaDUF966-14A | - | *Helianthus annuus* | core promoter element around -30 of transcription start |
| TaDUF966-14A | - | *Arabidopsis thaliana* | core promoter element around -30 of transcription start |
| TaDUF966-14A | + | *Arabidopsis thaliana* | core promoter element around -30 of transcription start |
| TaDUF966-14A | - | *Helianthus annuus* | core promoter element around -30 of transcription start |
| TaDUF966-14A | - | *Arabidopsis thaliana* | core promoter element around -30 of transcription start |
| TaDUF966-14A | + | *Arabidopsis thaliana* | core promoter element around -30 of transcription start |
| TaDUF966-14A | + | *Oryza sativa* | core promoter element around -30 of transcription start |
| TaDUF966-14A | + | *Brassica napus* | core promoter element around -30 of transcription start |
| TaDUF966-14A | - | *Arabidopsis thaliana* | core promoter element around -30 of transcription start |
| TaDUF966-14A | + | *Arabidopsis thaliana* | core promoter element around -30 of transcription start |
| TaDUF966-14A | - | *Arabidopsis thaliana* | core promoter element around -30 of transcription start |
| TaDUF966-14A | - | *Helianthus annuus* | core promoter element around -30 of transcription start |
| TaDUF966-14A | - | *Arabidopsis thaliana* | core promoter element around -30 of transcription start |
| TaDUF966-14A | - | *Nicotiana tabacum* | cis-acting element involved in salicylic acid responsiveness |
| TaDUF966-14A | - | *Hordeum vulgare* | cis-acting regulatory element involved in the MeJA-responsiveness |
| TaDUF966-14A | - | *Brassica oleracea* | auxin-responsive element |
| TaDUF966-14B | - | *Arabidopsis thaliana* | cis-acting element involved in the abscisic acid responsiveness |
| TaDUF966-14B | - | *Zea mays* | cis-acting regulatory element essential for the anaerobic induction |
| TaDUF966-14B | - | *Petroselinum crispum* | part of a conserved DNA module involved in light responsiveness |
| TaDUF966-14B | - | *Nicotiana glutinosa* |  |
| TaDUF966-14B | + | *Nicotiana glutinosa* |  |
| TaDUF966-14B | + | *Nicotiana glutinosa* |  |
| TaDUF966-14B | + | *Arabidopsis thaliana* | common cis-acting element in promoter and enhancer regions |
| TaDUF966-14B | + | *Nicotiana glutinosa* |  |
| TaDUF966-14B | - | *Nicotiana glutinosa* |  |
| TaDUF966-14B | - | *Pisum sativum* | common cis-acting element in promoter and enhancer regions |
| TaDUF966-14B | - | *Pisum sativum* | common cis-acting element in promoter and enhancer regions |
| TaDUF966-14B | + | *Pisum sativum* | common cis-acting element in promoter and enhancer regions |
| TaDUF966-14B | - | *Arabidopsis thaliana* | common cis-acting element in promoter and enhancer regions |
| TaDUF966-14B | - | *Nicotiana glutinosa* |  |
| TaDUF966-14B | + | *Nicotiana glutinosa* |  |
| TaDUF966-14B | - | *Pisum sativum* | common cis-acting element in promoter and enhancer regions |
| TaDUF966-14B | - | *Nicotiana glutinosa* |  |
| TaDUF966-14B | - | *Nicotiana glutinosa* |  |
| TaDUF966-14B | - | *Pisum sativum* | common cis-acting element in promoter and enhancer regions |
| TaDUF966-14B | - | *Pisum sativum* | common cis-acting element in promoter and enhancer regions |
| TaDUF966-14B | + | *Hordeum vulgare* | cis-acting regulatory element involved in the MeJA-responsiveness |
| TaDUF966-14B | + | *Hordeum vulgare* | cis-acting regulatory element involved in the MeJA-responsiveness |
| TaDUF966-14B | + | *Pisum sativum* | cis-acting regulatory element involved in light responsiveness |
| TaDUF966-14B | - | *Zea mays* | cis-acting regulatory element involved in light responsiveness |
| TaDUF966-14B | + | *Arabidopsis thaliana* | light responsive element |
| TaDUF966-14B | + | *Arabidopsis thaliana* | MYB binding site involved in drought-inducibility |
| TaDUF966-14B | - | *Hordeum vulgare* | cis-acting element involved in low-temperature responsiveness |
| TaDUF966-14B | + | *Petroselinum crispum* | MYB binding site involved in light responsiveness |
| TaDUF966-14B | + | *Petroselinum crispum* | MYB binding site involved in light responsiveness |
| TaDUF966-14B | + | *Oryza sativa* | light responsive element |
| TaDUF966-14B | + | *Oryza sativa* | light responsive element |
| TaDUF966-14B | + | *Arabidopsis thaliana* | core promoter element around -30 of transcription start |
| TaDUF966-14B | - | *Helianthus annuus* | core promoter element around -30 of transcription start |
| TaDUF966-14B | + | *Arabidopsis thaliana* | core promoter element around -30 of transcription start |
| TaDUF966-14B | + | *Oryza sativa* | core promoter element around -30 of transcription start |
| TaDUF966-14B | - | *Arabidopsis thaliana* | core promoter element around -30 of transcription start |
| TaDUF966-14B | - | *Arabidopsis thaliana* | core promoter element around -30 of transcription start |
| TaDUF966-14B | - | *Arabidopsis thaliana* | core promoter element around -30 of transcription start |
| TaDUF966-14B | - | *Arabidopsis thaliana* | core promoter element around -30 of transcription start |
| TaDUF966-14B | - | *Nicotiana tabacum* | cis-acting element involved in defense and stress responsiveness |
| TaDUF966-14B | - | *Hordeum vulgare* | cis-acting regulatory element involved in the MeJA-responsiveness |
| TaDUF966-14B | - | *Hordeum vulgare* | cis-acting regulatory element involved in the MeJA-responsiveness |
| TaDUF966-14D | - | *Arabidopsis thaliana* | cis-acting element involved in the abscisic acid responsiveness |
| TaDUF966-14D | - | *Arabidopsis thaliana* | cis-acting element involved in the abscisic acid responsiveness |
| TaDUF966-14D | + | *Arabidopsis thaliana* | cis-acting element involved in the abscisic acid responsiveness |
| TaDUF966-14D | - | *Hordeum vulgare* | cis-acting element involved in the abscisic acid responsiveness |
| TaDUF966-14D | + | *Arabidopsis thaliana* | cis-acting element involved in the abscisic acid responsiveness |
| TaDUF966-14D | + | *Zea mays* | cis-acting regulatory element essential for the anaerobic induction |
| TaDUF966-14D | + | *Zea mays* | cis-acting regulatory element essential for the anaerobic induction |
| TaDUF966-14D | - | *Nicotiana glutinosa* |  |
| TaDUF966-14D | + | *Nicotiana glutinosa* |  |
| TaDUF966-14D | - | *Arabidopsis thaliana* | common cis-acting element in promoter and enhancer regions |
| TaDUF966-14D | + | *Nicotiana glutinosa* |  |
| TaDUF966-14D | + | *Nicotiana glutinosa* |  |
| TaDUF966-14D | - | *Nicotiana glutinosa* |  |
| TaDUF966-14D | + | *Nicotiana glutinosa* |  |
| TaDUF966-14D | + | *Nicotiana glutinosa* |  |
| TaDUF966-14D | + | *Nicotiana glutinosa* |  |
| TaDUF966-14D | + | *Nicotiana glutinosa* |  |
| TaDUF966-14D | + | *Nicotiana glutinosa* |  |
| TaDUF966-14D | - | *Arabidopsis thaliana* | common cis-acting element in promoter and enhancer regions |
| TaDUF966-14D | - | *Nicotiana glutinosa* |  |
| TaDUF966-14D | + | *Nicotiana glutinosa* |  |
| TaDUF966-14D | + | *Pisum sativum* | common cis-acting element in promoter and enhancer regions |
| TaDUF966-14D | + | *Pisum sativum* | common cis-acting element in promoter and enhancer regions |
| TaDUF966-14D | + | *Nicotiana glutinosa* |  |
| TaDUF966-14D | + | *Nicotiana glutinosa* |  |
| TaDUF966-14D | + | *Pisum sativum* | common cis-acting element in promoter and enhancer regions |
| TaDUF966-14D | + | *Nicotiana glutinosa* |  |
| TaDUF966-14D | - | *Nicotiana glutinosa* |  |
| TaDUF966-14D | + | *Pisum sativum* | cis-acting regulatory element involved in light responsiveness |
| TaDUF966-14D | + | *Pisum sativum* | cis-acting regulatory element involved in light responsiveness |
| TaDUF966-14D | - | *Zea mays* | cis-acting regulatory element involved in light responsiveness |
| TaDUF966-14D | - | *Zea mays* | cis-acting regulatory element involved in light responsiveness |
| TaDUF966-14D | - | *Zea mays* | cis-acting regulatory element involved in light responsiveness |
| TaDUF966-14D | + | *Arabidopsis thaliana* | MYB binding site involved in drought-inducibility |
| TaDUF966-14D | - | *Zea mays* | cis-acting regulatory element involved in zein metabolism regulation |
| TaDUF966-14D | + | *Brassica napus* | core promoter element around -30 of transcription start |
| TaDUF966-14D | - | *Arabidopsis thaliana* | core promoter element around -30 of transcription start |
| TaDUF966-14D | + | *Arabidopsis thaliana* | core promoter element around -30 of transcription start |
| TaDUF966-14D | + | *Arabidopsis thaliana* | core promoter element around -30 of transcription start |
| TaDUF966-14D | + | *Arabidopsis thaliana* | core promoter element around -30 of transcription start |
| TaDUF966-14D | + | *Brassica napus* | core promoter element around -30 of transcription start |
| TaDUF966-14D | + | *Arabidopsis thaliana* | core promoter element around -30 of transcription start |
| TaDUF966-14D | + | *Brassica napus* | core promoter element around -30 of transcription start |
| TaDUF966-14D | + | *Arabidopsis thaliana* | core promoter element around -30 of transcription start |
| TaDUF966-14D | - | *Arabidopsis thaliana* | part of a light responsive element |
| TaDUF966-14D | + | *Nicotiana tabacum* | cis-acting element involved in defense and stress responsiveness |

Supplementary Table 11. | Metadata for RNA-Seq samples. Details for each sample including variety, tissue, age, stress conditions and original publication

| **Treatment Type** | **Provider** | **SRA ID** | **Scientific Name** | **Variety** | **Age** | **Stress** | **Tissue** | **DOI** |
| --- | --- | --- | --- | --- | --- | --- | --- | --- |
| CK 6h root of 1 week QM6 | SRR2306553 | SRP062745 | *Triticum aestivum* | Qing Mai 6 | 1 week | 6 hour of ck | root | doi: 10.1038/srep21476 |
| salt 6h root of 1 week QM6 | SRR2306554 | SRP062745 | *Triticum aestivum* | Qing Mai 6 | 1 week | 6 hour of salt | root | doi: 10.1038/srep21476 |
| CK 12h root of 1 week QM6 | SRR2306555 | SRP062745 | *Triticum aestivum* | Qing Mai 6 | 1 week | 12 hour of ck | root | doi: 10.1038/srep21476 |
| salt 12h root of 1 week QM6 | SRR2306557 | SRP062745 | *Triticum aestivum* | Qing Mai 6 | 1 week | 12 hour of salt | root | doi: 10.1038/srep21476 |
| CK 24h root of 1 week QM6 | SRR2306556 | SRP062745 | *Triticum aestivum* | Qing Mai 6 | 1 week | 24 hour of ck | root | doi: 10.1038/srep21476 |
| salt 24h root of 1 week QM6 | SRR2306558 | SRP062745 | *Triticum aestivum* | Qing Mai 6 | 1 week | 24 hour of salt | root | doi: 10.1038/srep21476 |
| CK 48h root of 1 week QM6 | SRR2306559 | SRP062745 | *Triticum aestivum* | Qing Mai 6 | 1 week | 48 hour of ck | root | doi: 10.1038/srep21476 |
| salt 48h root of 1 week QM6 | SRR2306560 | SRP062745 | *Triticum aestivum* | Qing Mai 6 | 1 week | 48 hour of salt | root | doi: 10.1038/srep21476 |
| CK 6h root of CS | SRR2306545 | SRP062745 | *Triticum aestivum* | ChineseSpring | 1 week | 6 hour of ck | root | [doi: 10.1038/srep21476](https://dx.doi.org/10.1038%2Fsrep21476) |
| salt 6h root of 1 week CS | SRR2306546 | SRP062745 | *Triticum aestivum* | ChineseSpring | 1 week | 6 hour of salt | root | doi: 10.1038/srep21476 |
| CK 12h root of 1 week CS | SRR2306547 | SRP062745 | *Triticum aestivum* | ChineseSpring | 1 week | 12 hour of ck | root | doi: 10.1038/srep21476 |
| salt 12h root of 1 week CS | SRR2306549 | SRP062745 | *Triticum aestivum* | ChineseSpring | 1 week | 12 hour of salt | root | doi: 10.1038/srep21476 |
| CK 24h root of 1 week CS | SRR2306548 | SRP062745 | *Triticum aestivum* | ChineseSpring | 1 week | 24 hour of ck | root | doi: 10.1038/srep21476 |
| salt 24h root of 1 week CS | SRR2306550 | SRP062745 | *Triticum aestivum* | ChineseSpring | 1 week | 24 hour of salt | root | doi: 10.1038/srep21476 |
| CK 48h root of 1 week CS | SRR2306551 | SRP062745 | *Triticum aestivum* | ChineseSpring | 1 week | 48 hour of ck | root | doi: 10.1038/srep21476 |
| salt 48h root of 1 week CS | SRR2306552 | SRP062745 | *Triticum aestivum* | ChineseSpring | 1 week | 48 hour of salt | root | doi: 10.1038/srep21476 |

Continue to the previous **Supplementary Table 11.**

| **Treatment Type** | **RNA expression profile data（TPM)** | | | | | | |
| --- | --- | --- | --- | --- | --- | --- | --- |
|  | **TaDUF966-5A** | **TaDUF966-5D** | **TaDUF966-5B** | **TaDUF966-1A** | **TaDUF966-7D** | **TaDUF966-4B** | **TaDUF966-4D** |
| CK 6h root of 1 week QM6 | 0.8 | 1.1 | 0.2 | 0.6 | 0 | 0.3 | 0.1 |
| salt 6h root of 1 week QM6 | 9.7 | 10.8 | 3.7 | 0.4 | 0 | 0.5 | 0.4 |
| CK 12h root of 1 week QM6 | 0.5 | 1 | 0 | 0.8 | 0 | 0.3 | 0.2 |
| salt 12h root of 1 week QM6 | 7.8 | 5.6 | 2 | 0.3 | 0 | 0.2 | 0.4 |
| CK 24h root of 1 week QM6 | 0.7 | 0.9 | 0 | 0.7 | 0 | 0.4 | 0.1 |
| salt 24h root of 1 week QM6 | 6.2 | 3.5 | 0.5 | 0.3 | 0.1 | 0.1 | 0.1 |
| CK 48h root of 1 week QM6 | 0.6 | 1.3 | 0.1 | 0.7 | 0 | 0.3 | 0.1 |
| salt 48h root of 1 week QM6 | 3.4 | 3.3 | 0.2 | 0.3 | 0 | 0.1 | 0.1 |
| CK 6h root of CS | 0.5 | 1.3 | 0.2 | 0.4 | 0 | 0.5 | 0.2 |
| salt 6h root of 1 week CS | 4.9 | 15.4 | 2.5 | 0.2 | 0 | 0.3 | 0.1 |
| CK 12h root of 1 week CS | 0.5 | 0.7 | 0.1 | 0.7 | 0 | 0.2 | 0.1 |
| salt 12h root of 1 week CS | 4.2 | 9.1 | 1.3 | 0.3 | 0 | 0.4 | 0.2 |
| CK 24h root of 1 week CS | 0.3 | 1 | 0.1 | 0.3 | 0 | 0.2 | 0.2 |
| salt 24h root of 1 week CS | 4.1 | 7 | 1.2 | 0.4 | 0 | 0.5 | 0.3 |
| CK 48h root of 1 week CS | 0.2 | 0.8 | 0.2 | 0.5 | 0 | 0.2 | 0.1 |
| salt 48h root of 1 week CS | 2.1 | 3.5 | 0.6 | 0.4 | 0 | 0 | 0 |

Continue to the previous **Supplementary Table 11.**

| **Treatment Type** | **RNA expression profile data（TPM)** | | | | | | |
| --- | --- | --- | --- | --- | --- | --- | --- |
|  | **TaDUF966-4A** | **TaDUF966-3B** | **TaDUF966-3D** | **TaDUF966-3A** | **TaDUF966-8A** | **TaDUF966-8D** | **TaDUF966-8B** |
| CK 6h root of 1 week QM6 | 0.4 | 0.1 | 0.1 | 0 | 0 | 0 | 0 |
| salt 6h root of 1 week QM6 | 0.3 | 0.2 | 0 | 0 | 0 | 0 | 0 |
| CK 12h root of 1 week QM6 | 0.3 | 0.2 | 0.1 | 0.3 | 0 | 0 | 0 |
| salt 12h root of 1 week QM6 | 0.2 | 0 | 0.1 | 0 | 0 | 0 | 0 |
| CK 24h root of 1 week QM6 | 0.1 | 0.4 | 0.1 | 0.4 | 0 | 0 | 0 |
| salt 24h root of 1 week QM6 | 0.2 | 0.1 | 0.2 | 0 | 0 | 0 | 0 |
| CK 48h root of 1 week QM6 | 0.2 | 0.4 | 0.1 | 0.1 | 0 | 0 | 0 |
| salt 48h root of 1 week QM6 | 0.1 | 0 | 0.4 | 0 | 0 | 0 | 0 |
| CK 6h root of CS | 0.1 | 0 | 0 | 0 | 0 | 0 | 0 |
| salt 6h root of 1 week CS | 0.4 | 0.1 | 0.1 | 0 | 0 | 0 | 0 |
| CK 12h root of 1 week CS | 0.5 | 0.1 | 0 | 0.3 | 0 | 0 | 0 |
| salt 12h root of 1 week CS | 0.3 | 0 | 0.3 | 0 | 0 | 0 | 0 |
| CK 24h root of 1 week CS | 0.3 | 0.2 | 0.2 | 0.4 | 0 | 0 | 0 |
| salt 24h root of 1 week CS | 0.2 | 0.1 | 0.4 | 0.1 | 0 | 0 | 0 |
| CK 48h root of 1 week CS | 0.1 | 0.3 | 0.1 | 0.4 | 0 | 0 | 0 |
| salt 48h root of 1 week CS | 0.1 | 0.1 | 1 | 0 | 0 | 0 | 0 |

Continue to the previous **Supplementary Table 11.**

| **Treatment Type** | **RNA expression profile data（TPM)** | | | | | | |
| --- | --- | --- | --- | --- | --- | --- | --- |
|  | **TaDUF966-11B** | **TaDUF966-13A** | **TaDUF966-6B** | **TaDUF966-12B** | **TaDUF966-9B** | **TaDUF966-9D** | **TaDUF966-9A** |
| CK 6h root of 1 week QM6 | 0 | 0 | 0 | 0 | 1.6 | 2.5 | 0.7 |
| salt 6h root of 1 week QM6 | 0 | 0 | 0 | 0 | 10.2 | 2.3 | 2.2 |
| CK 12h root of 1 week QM6 | 0 | 0 | 0 | 0 | 1.7 | 1.8 | 0.6 |
| salt 12h root of 1 week QM6 | 0 | 0 | 0 | 0 | 6.4 | 1.2 | 1.4 |
| CK 24h root of 1 week QM6 | 0 | 0 | 0 | 0 | 1.8 | 1.9 | 1 |
| salt 24h root of 1 week QM6 | 0 | 0 | 0 | 0 | 3.1 | 0.5 | 0.9 |
| CK 48h root of 1 week QM6 | 0 | 0 | 0 | 0 | 1.9 | 2.5 | 1.1 |
| salt 48h root of 1 week QM6 | 0 | 0 | 0 | 0 | 1 | 0.2 | 0.3 |
| CK 6h root of CS | 0 | 0 | 0 | 0 | 1 | 2.5 | 0.5 |
| salt 6h root of 1 week CS | 0 | 0 | 0 | 0 | 5 | 3.3 | 1 |
| CK 12h root of 1 week CS | 0 | 0 | 0 | 0 | 1.1 | 1.8 | 0.5 |
| salt 12h root of 1 week CS | 0 | 0 | 0 | 0 | 2.6 | 1.6 | 0.7 |
| CK 24h root of 1 week CS | 0 | 0 | 0 | 0 | 1 | 2.1 | 0.3 |
| salt 24h root of 1 week CS | 0 | 0 | 0 | 0 | 0.4 | 0.9 | 0.6 |
| CK 48h root of 1 week CS | 0 | 0 | 0 | 0 | 1.5 | 2.1 | 0.4 |
| salt 48h root of 1 week CS | 0 | 0 | 0 | 0 | 0.1 | 0.2 | 0.1 |

Continue to the previous **Supplementary Table 11.**

| **Treatment Type** | **RNA expression profile data（TPM)** | | | | | | |
| --- | --- | --- | --- | --- | --- | --- | --- |
|  | **TaDUF966-2B** | **TaDUF966-14A** | **TaDUF966-14B** | **TaDUF966-14D** | **TaDUF966-10A** | **TaDUF966-10D** | **TaDUF966-10B** |
| CK 6h root of 1 week QM6 | 0.2 | 1.3 | 1.9 | 1.5 | 0.2 | 0.3 | 0.5 |
| salt 6h root of 1 week QM6 | 0.1 | 2.1 | 2.5 | 2.6 | 0.5 | 1.5 | 1.1 |
| CK 12h root of 1 week QM6 | 0.2 | 1 | 1.8 | 0.9 | 0.2 | 0.5 | 0.5 |
| salt 12h root of 1 week QM6 | 0.1 | 1.9 | 1.9 | 2 | 0.2 | 0.9 | 0.9 |
| CK 24h root of 1 week QM6 | 0.3 | 1.2 | 1.9 | 1.6 | 0.1 | 0.4 | 0.5 |
| salt 24h root of 1 week QM6 | 0.1 | 1 | 1 | 1 | 0.3 | 1.1 | 0.4 |
| CK 48h root of 1 week QM6 | 0.2 | 1 | 1.8 | 0.9 | 0.2 | 0.4 | 0.4 |
| salt 48h root of 1 week QM6 | 0 | 0.7 | 0.4 | 0.2 | 0.2 | 0.6 | 0.1 |
| CK 6h root of CS | 0.7 | 1.1 | 2.2 | 1.2 | 0.2 | 0.4 | 0.3 |
| salt 6h root of 1 week CS | 0.7 | 3.1 | 3.3 | 3 | 0.5 | 1.1 | 1.2 |
| CK 12h root of 1 week CS | 0.6 | 1 | 1.5 | 1 | 0.2 | 0.2 | 0.3 |
| salt 12h root of 1 week CS | 0.3 | 1.9 | 1.9 | 2.1 | 0.3 | 0.8 | 0.9 |
| CK 24h root of 1 week CS | 0.9 | 1.1 | 2.2 | 1.5 | 0.4 | 0.3 | 0.4 |
| salt 24h root of 1 week CS | 0.2 | 1.1 | 0.8 | 1 | 0.2 | 0.4 | 0.2 |
| CK 48h root of 1 week CS | 1.1 | 0.9 | 2.3 | 1.2 | 0.2 | 0.3 | 0.3 |
| salt 48h root of 1 week CS | 0.1 | 0.5 | 0.3 | 0.5 | 0.1 | 0.1 | 0 |
